# Supplementary material for: Yellow Twig (Nauclea orientalis) from Thailand: Strictosamide as the Key Alkaloid of This Plant Species
Source: Molecules. 2022 Aug 14;27(16):5176. doi: 10.3390/molecules27165176 (PMC9414341; doi:10.3390/molecules27165176)
Supplement: Supplementary file 1 [file molecules-27-05176-s001.zip › molecules-1839275-supplementary.pdf]

SUPPLEMENTARY MATERIAL

Yellow Twig (*Nauclea orientalis*) from Thailand: Strictosamide as the key alkaloid of this plant species

Weerasak Songoen [a,b], Julia Brunmair [c], Florian Traxler [b], Viktoria Chiara Wieser [d], Witthawat Phanchai [e], Wanchai Pluempanupat [a,*], Lothar Brecker [b,*], Johann Schinnerl [d,*]

[a] *Department of Chemistry and Center of Excellence for Innovation in Chemistry, Special Research Unit for Advanced Magnetic Resonance, Faculty of Science, Kasetsart University, Bangkok 10900, Thailand.*

[b] *Department of Organic Chemistry, University of Vienna, Währinger Strasse 38, A-1090 Vienna, Austria.*

[c] *Department of Analytical Chemistry, University of Vienna, Währinger Strasse 38, A-1090 Vienna, Austria.*

[d] *Department of Botany and Biodiversity Research, University of Vienna, Rennweg 14, A-1030 Vienna, Austria.*

[e] *Department of Physics, Faculty of Science, Khon Kaen University, Khon Kaen 40002, Thailand.*

* Correspondence:
Johann Schinnerl (johann.schinnerl@univie.ac.at)
Lothar Brecker (lothar.brecker@univie.ac.at)
Wanchai Pluempanupat (fsciwcp@ku.ac.th)

Table of Contents

S1.0. Detailed isolation procedure 5

S1.1. Enzymatic deglucosylation of strictosamide (**1**) 6

S1.2. Quantification of strictosamide (**1**) 6

S1.3. Gustatory experiment 7

S1.4. Non-choice feeding assay 7

S2.1. Isolated compounds 8

2.1.1. Strictosamide (**1**) 8

2.1.2. 10-Hydroxystrictosamide (**2**) 8

2.1.3. 10-Hydroxyvincoside lactam (**3**) 8

2.1.4. 10-Hydroxy-3-*epi*-pumiloside (**4**) 8

2.1.5. 3-*Epi*-pumiloside (**5**) 8

2.1.6. Nauclealomide B (**6**) 8

2.1.7. Nauclealomide C (**7**) 9

2.1.8. Paratunamide C (**8**) 9

2.1.9. Angustine (**9**) 9

2.1.10. 3,14-Dihydroangustine (**10**) 9

2.1.11. 18,19-Dihydroangustine (**11**) 9

2.1.12. Angustoline (**12**) 9

2.1.13. 3,14-Dihydroangustoline (**13**) 9

2.1.14. (Z)-2-((2*S*,12b*S*,*Z*)-3-(hydroxymethylene)-4-oxo-1,2,3,4,6,7,12,12b-octahydroindolo[2,3-a]quinolizin-2-yl)but-2-enal (**1a**) 10

S3.1. NMR data of the isolated compounds 11

Table S1. ^{1}H and ^{13}C NMR spectroscopic data [ppm] of strictosamide (**1**), 10-hydroxy strictosamide (**2**) and 10-hydroxyvincoside lactam (**3**) recorded in CD_3OD. 11

Table S2. ^{1}H and ^{13}C NMR spectroscopic data [ppm] of 10-hydroxy-3-*epi*-pumiloside (**4**) and 3-*epi*-pumiloside (**5**) recorded in CD_3OD. 12

Table S3. ^{1}H and ^{13}C NMR spectroscopic data [ppm] of nauclealomide B (**6**), nauclealomide C (**7**) and paratunamide C (**8**) recorded in CD_3OD. 13

Table S4. ^{1}H and ^{13}C NMR spectroscopic data [ppm] of angustine (**9**), 3,14-dihydroangustine (**10**), and 18,19-dihydroangustine (**11**), recorded in CD_3OD. 14

Table S5. ^{1}H and ^{13}C NMR spectroscopic data [ppm] of angustoline (**12**), 3,14-dihydro angustoline (**13**) and **1a** recorded in CD_3OD. 15

Figure S1. ^{1}H NMR of strictosamide (**1**) in CD_3OD. 16
Figure S2. ^{13}C NMR of strictosamide (**1**) in CD_3OD. 17
Figure S3. Mass spectrum of strictosamide (**1**). 18
Figure S4. ^{1}H NMR of **1a** in CD_3OD. 19
Figure S5. ^{13}C NMR of **1a** in CD_3OD. 20
Figure S6. Mass spectrum of **1a**. 21
Figure S7. ^{1}H NMR of 10-hydroxy strictosamide (**2**) in CD_3OD. 22
Figure S8. ^{13}C NMR of 10-hydroxy strictosamide (**2**) in CD_3OD. 23
Figure S9. Mass spectrum of 10-hydroxy strictosamide (**2**). 24
Figure S10. ^{1}H NMR of 10-hydroxyvincoside lactam (**3**) in CD_3OD. 25
Figure S11. ^{13}C NMR of 10-hydroxyvincoside lactam (**3**) in CD_3OD. 25
Figure S12. Mass spectrum of 10-hydroxyvincoside lactam (**3**). 26
Figure S13. ^{1}H NMR of 10-hydroxy-3-*epi*-pumiloside (**4**) in CD_3OD. 27
Figure S14. ^{13}C NMR of 10-hydroxy-3-*epi*-pumiloside (**4**) in CD_3OD. 28
Figure S15. COSY of 10-hydroxy-3-*epi*-pumiloside (**4**) in CD_3OD. 30
Figure S16. Zoom COSY of 10-hydroxy-3-*epi*-pumiloside (**4**) in CD_3OD. 30
Figure S17. TOCSY of 10-hydroxy-3-*epi*-pumiloside (**4**) in CD_3OD. 31
Figure S18. Zoom TOCSY of 10-hydroxy-3-*epi*-pumiloside (**4**) in CD_3OD. 31
Figure S19. HSQC of 10-hydroxy-3-*epi*-pumiloside (**4**) in CD_3OD. 32
Figure S20. HMBC of 10-hydroxy-3-*epi*-pumiloside (**4**) in CD_3OD. 32
Figure S21. NOESY of 10-hydroxy-3-*epi*-pumiloside (**4**) in CD_3OD. 33
Figure S22. Mass spectrum of 10-hydroxy-3-*epi*-pumiloside (**4**). 34
Figure S23. ^{1}H NMR of 3-*epi*-pumiloside (**5**) in CD_3OD. 35
Figure S24. ^{13}C NMR of 3-*epi*-pumiloside (**5**) in CD_3OD. 36
Figure S25. Mass spectrum of 3-*epi*-pumiloside (**5**). 37
Figure S26. ^{1}H NMR of nauclealomide B (**6**) in CD_3OD. 38
Figure S27. ^{13}C NMR of nauclealomide B (**6**) in CD_3OD. 39
Figure S28. Mass spectrum of nauclealomide B (**6**). 40
Figure S29. ^{1}H NMR of nauclealomide C (**7**) in CD_3OD. 41
Figure S30. ^{13}C NMR of nauclealomide C (**7**) in CD_3OD. 42
Figure S31. Mass spectrum of nauclealomide C (**7**). 43
Figure S32. ^{1}H NMR of paratunamide C (**8**) in CD_3OD. 44

Figure S33. ^{13}C NMR of paratunamide C (**8**) in CD_3OD. 45

Figure S34. Mass spectrum of paratunamide C (**8**). 46

Figure S35. ^{1}H NMR of angustine (**9**) in CD_3OD. 47

Figure S36. ^{13}C NMR of angustine (**9**) in CD_3OD. 48

Figure S37. Mass spectrum of angustine (**9**). 49

Figure S38. ^{1}H NMR of 3,14-dihydroangustine (**10**) in CD_3OD. 50

Figure S39. ^{13}C NMR of 3,14-dihydroangustine (**10**) in CD_3OD. 51

Figure S40. Mass spectrum of 3,14-dihydroangustine (**10**). 52

Figure S41. ^{1}H NMR of 18,19-dihydroangustine (**11**) in CD_3OD. 53

Figure S42. ^{13}C NMR of 18,19-dihydroangustine (**11**) in CD_3OD. 54

Figure S43. Mass spectrum of 18,19-dihydroangustine (**11**). 55

Figure S44. ^{1}H NMR of angustoline (**12**) in CD_3OD. 56

Figure S45. ^{13}C NMR of angustoline (**12**) in CD3OD. 57

Figure S46. Mass spectrum of angustoline (**12**). 58

Figure S47. ^{1}H NMR of 3,14-dihydro angustoline (**13**) in CD_3OD. 59

Figure S48. ^{13}C NMR of 3,14-dihydro angustoline (**13**) in CD_3OD. 60

Figure S49. Mass spectrum of 3,14-dihydro angustoline (**13**). 61

Figure S50. LC-MS screening of the crude methanolic extracts from leaves and stem bark of *Nauclea orientalis*. 62

Figure S51. HPLC profile of methanolic extracted of leaves (**A**), bark (**B**) and wood (**C**). 62

Figure S52. Above: With methanol rinsed cutting surfaces of *N. orientalis*. 63

Figure S53. HPLC profiles of the rinsed cutting surfaces of the twigs of *N. orientalis*. 64

References 65

S1.0. Detailed isolation procedure

Isolation from leaves

The air-dried ground leaves of *N. orientalis* (2.4 kg) were sequentially extracted (3 × 7 d) with PE, CH_2Cl_2, EtOAc and MeOH. Each crude extract was filtered and evaporated under reduced pressure using a rotary evaporator. The methanolic extract (162.6 g) was further partitioned between distilled water, 5% EtOAc in PE, $CHCl_3$ (3.0 g) and EtOAc (14.7 g). The obtained fractions were further purified by appropriate chromatographic techniques and each separation step was monitored by TLC and HPLC. The chloroform fraction (3.0 g) was initially chromatographed by medium pressure liquid chromatography (MPLC) over silica gel. The elution system started with PE, EtOAc and MeOH with increasing polarity which afforded 32 subfractions named NO-M01-F1 to F32.

The combined fractions of NO-M01-F30 and NO-M01-F31 (48.2 mg) were chromatographed over Sephadex LH20 eluted with MeOH, yielding 4.6 mg of impure **4**. The fraction impure **4** was further purified by pTLC eluted with 50% of MeOH in $CHCl_3$. This step afforded 1.9 mg of **4**. Separation of fraction NO-M01-F24 (145.3 mg) by Sephadex LH20/MeOH afforded 25.3 mg of **1**. From fraction NO-M01-F25, 64.7 mg was subjected to SEC over Sephadex LH20/MeOH. This step furnished 11.4 mg of **1**. The 1.1 mg of **2** has been isolated from fraction NO-M01-F26 (68.6 mg) by Sephadex LH20 eluted with MeOH.

Isolation from stembark

Air-dried ground stembark of *N. orientalis* (2.4 kg) were subsequently extracted with PE, CH_2Cl_2, EtOAc and MeOH, respectively (3 × 7 d). All extracts were filtered and evaporated under reduced pressure in a rotary evaporator. The EtOAc extract (7.1 g) was chromatographed over silica gel column chromatography gradually eluted from 60% EtOAc in PE to 60% MeOH in EtOAc. The separation afforded ten subfractions (NO-EC01-F01 to F10). The fraction NO-EC01-F10 (750 mg) was chromatographed over Sephadex LH20/MeOH. This step afforded six fractions named NO-ES01-F01 to 06. The 117.1 mg of NO-ES01-F03 was subjected to MPLC over silica gel eluted gradually from 30% EtOAc in PE to 80% MeOH. This afforded 15.5 mg of impure **5**. Purification of **5** by MPLC yielded 5.1 mg.

The fraction NO-EC01-F09 (744 mg) was fractioned over silica gel column chromatography eluted isoratic with 10% MeOH in EtOAc. This step yielded seven fractions named NO-EC02-F01 to F07. The selected fraction NO-EC02-F05 (102.0 mg) was further chromatographed using preparative HPLC. This separation afforded 1.5 mg of **6**, 2.6 mg of **7** and 1.5 mg of **8**.

The CH_2Cl_2 extract (7.6 g) was fractionated over silica gel column chromatography gradually eluted with mixtures consisting of PE, EtOAc and MeOH with increasing polarity. This separation step gave 17 sub-fractions named NO-DC01-F01 to F17. Fraction NO-DC01-F13 (168.2 mg was further purified by Sephadex LH20/MeOH. This furnished 2.3 mg of **9**, 90.7 mg of impure **10** and 10.4 mg of impure **11**. Compound **10** (1.2 mg) was purified by Sephadex LH20/ MeOH, while compound **11** (2.1 mg) has been obtained from pTLC elution with 5% MeOH in EtOAc. The separation of fraction NO-

DC01-F15 (451.3 mg) by Sephadex LH20/MeOH afforded 8.5 mg of **12** and 14.8 mg of **13** in a 1:2 diastereomeric ratio.

Isolation from wood

Air-dried ground wood of *N. orientalis* (400 g) was extracted with MeOH (3 × 7 d). The MeOH extract (12.2 g) was filtered and evaporated under reduced pressure in a rotary evaporator and subsequently partitioned between distilled water and *n*-butanol. This afforded 4.9 g of crude *n*-butanolic extract. The *n*-butanol phase was further chromatographed by MPLC over silica gel using a steps-wise gradient elution started with 60% EtOAc in PE to 80% MeOH in EtOAc. This step afforded 188 mg of impure strictosamide (**1**). Final purification over Sephadex LH20/MeOH yielded 95.6 mg of strictosamide (**1**).

S1.1. Enzymatic deglucosylation of strictosamide (**1**)

The enzymatic deglucosylation of strictosamide (**1**; 0.14 mmol) was carried out in 10% DMSO in phosphate buffer (20 mM, pH 6.0) at RT for 6 d. The β-glucosidase CelB (50 U final concentration) [S1, S2] was used for this hydrolysis. The final concentration of strictosamide (**1**) was 3.33 mM. After performing the reaction, the mixture was evaporated and purified by CC over Sephadex LH20 eluted with MeOH. This step afforded ten fractions named SE02-F01 to F10. Fraction SE08 was further partitioned between distilled water and $CHCl_3$. The $CHCl_3$ residue (53.7 mg) was further purified by pTLC eluted with 25% of EtOAc in PE. This afforded 5.0 mg of **1a**. For further investigation of compound **1a**, the CelB catalyzed hydrolyses of strictosamide (**1**) was repeated in larger scale in non-deuterated solvent (10% DMSO/H_2O (pH 6.0) 1:9). After a reaction time of 6 d, the reaction mixture was chromatographically separated and afforded compound **1a** as a mixture of different isomeric forms. This enzymatic deglucosylation was also performed for an *in situ* ^{1}H NMR monitoring following the work of Brecker & Ribbons [S3]. Therefore, the reaction was carried out in an NMR tube in 10% DMSO-d_6 in phosphate buffer (20 mM, pD 6.04) in D_2O at RT. ^{1}H NMR spectra were recorded in regular intervals.

S1.2. Quantification of strictosamide (**1**)

The quantification of **1** was carried out by using purified strictosamide (**1**) as external standard. The stock solution of strictosamide (**1**) (1.2 mg mL^{-1}) was prepared in MeOH. From this stock solution a dilution series comprising five standards in the concentration of 75, 150, 300, 600 and 1200 μg mL^{-1} was prepared. Each standard solution was analyzed by HPLC three times. The calibration curve was obtained by plotting between the mean of the peak areas *versus* concentration of strictosamide (**1**). For quantification, dried leaves, bark and wood of *N. orientalis* were ground and sieved (0.20 mm) prior extraction. The sieved residues were accurately weighed (100.1 mg) and extracted by MeOH (3 × 1.5 mL) under sonication for 15 min. The MeOH extract was centrifuged at 14,000 rpm for 20 min. Finally,

the supernatants were pooled, the solvent evaporated and the samples adjusted to 1.0 mg mL^{-1} in pure MeOH for HPLC analysis.

S1.3. Gustatory experiment

Briefly, each compound was distributed evenly on weighed leaf discs from commercial lettuce (*Lactuca sativa* L.). For compound **1** we used a concentration similar to its concentration in the leaves of *N. orientalis*. The leaf discs were distributed in 2, 4, 6, 8, 10 and 12 o'clock mode in the Petri dishes, the solutions applied accordingly and one caterpillar at the 3rd larval stage placed in the center. The leaf discs at position 10 was the control treated with the solvent mixture and at position 12 o'clock was an untreated leaf disc. After 24 h the leaf discs were weighed again, and the differences were calculated. We used the differences as a measure of attractiveness and compared them to the reference chemicals used. The leaf discs treated with solvent were used for all the calculations. No differences were observable between the leaf discs treated with the solvent mixture and the untreated leaf discs. Stock solutions of **1**, caffeine, aristolochic acid and D-salicine (5.0 mg mL^{-1} each) in acetone/water (90:10) were prepared and 10, 20 or 30 µL of each solution were distributed evenly on each leaf disc (13 mm in diameter, stamped out from a common lettuce) to reach different amounts of substances on the discs (Table 1). The chosen amount of **1** and the three other compounds were mimicking the conc. of **1** present in the leaves (see 2.3). After evaporation of the solvent each leaf disc was weighed. Humidified filter paper was put into a Petri dish (90 mm in diameter) and four of these leaf discs—one for each compound—were placed in positions 2, 4, 6 and 8 o'clock. In the center of each Petri dish a caterpillar of *S. littoralis* in the 3rd larval stage was placed, which had been starving for 16 h. The caterpillars were allowed to consume the leaf discs for 24 h with a light/darkness/light regime of 8/8/8 at 24°C. After this time the leaf discs were weighed again and the mass differences were used as a measure for the attractiveness of the compounds. The calculated differences were corrected by the loss of water. To assess the average loss of water, we treated 18 leaf discs in the same manner and evaluated their mass losses after 24 h under the same conditions as described above.

S1.4. Non-choice feeding assay

The insect assay was performed in triplicate with slight modifications according to [28]. Briefly, 184 mg of freeze-dried food powder containing ground white beans, yeast, ascorbic acid and ethyl *para*-hydroxy benzoate as preservative was spiked with 0.8, 0.4 and 0.2% of **1**, also mimicking its conc. in the leaves. After evaporation of the solvent (MeOH, 16 h), 363 µL of an aqueous consisting of 352 µL ddH_2O, 11.5 µL vitamin solution and 187.5 µg of the antibiotic chloramphenicol was added and the powder solidified by adding 0.6 mL of a warm (50 °C) agar-agar solution (5 g in 140 mL water). These food pellets were transferred into Petri dishes and a 3rd instar larvae of *S. littoralis* was placed on each food pellet. The Petri dishes were kept in an incubator at 26°C and 90% humidity in darkness for 72 h.

Afterwards the masses of the remaining food pellet assessed. Unspiked food pellets served as controls. All the calculated values were corrected by the loss of water.

S2.0. Isolated compounds

2.1.1. Strictosamide (**1**)

HR-TOF-ESI-MS *m/z* 521.1896 $[M+Na]^+$ (calcd 521.1900 for $C_{26}H_{30}N_2O_8Na^+$); ^1H NMR and ^{13}C NMR data in agreement with previous reported data [S4] and listed in Table S1. The ^1H and ^{13}C NMR spectra as well as the MS spectrum are shown in Figures S1–S3.

2.1.2. 10-Hydroxystrictosamide (**2**)

HR-TOF-ESI-MS *m/z* 537.1856 $[M+Na]^+$ (calcd 537.1849 for $C_{26}H_{30}N_2O_9Na^+$); ^1H NMR and ^{13}C NMR data in agreement with previous reported data [S5] and listed in Table S1. The ^1H and ^{13}C NMR spectra as well as the MS spectrum are shown in Figures S7–S9.

2.1.3 10-Hydroxyvincoside lactam (**3**)

HR-TOF-ESI-MS *m/z* 537.1845 $[M+Na]^+$ (calcd 537.1849 for $C_{26}H_{30}N_2O_9Na^+$); ^1H NMR and ^{13}C NMR data in agreement with previous reported data [S5] and listed in Table S1. The ^1H and ^{13}C NMR spectra as well as the MS spectrum are shown in Figures S10–S12.

2.1.4. 10-Hydroxy-3-*epi*-pumiloside (**4**)

$[\alpha]_D^{20} = -28.94$ (c 0.95 mg·mL^{-1}, MeOH); UV (MeOH) λ nm (log ε): 218 (3.92), 248 (3.96), 332 (3.26), 346 (3.23); HR-TOF-ESI-MS *m/z* 551.1635 $[M+Na]^+$ (calcd 551.1642 for $C_{26}H_{28}N_2O_{10}Na^+$); ^1H NMR and ^{13}C NMR data are listed in Table S2. The ^1H, ^{13}C and 2D NMR spectra as well as the MS spectrum are shown in Figures S13–S22.

2.1.5. 3-*Epi*-pumiloside (**5**)

HR-TOF-ESI-MS *m/z* 513.1846 $[M+H]^+$ (calcd 513.1873 for $C_{26}H_{29}N_2O_9^+$); ^1H NMR and ^{13}C NMR data in agreement with previous reported data [S6] and listed in Table S2. The ^1H and ^{13}C NMR spectra as well as the MS spectrum are shown in Figures S23–S25.

2.1.6. Nauclealomide B (**6**)

HR-TOF-ESI-MS *m/z* 553.1796 $[M+Na]^+$ (calcd 553.1798 for $C_{26}H_{30}N_2O_{10}Na^+$); ^1H NMR and ^{13}C NMR data in agreement with previous reported data [S7] and listed in Table S3. The ^1H and ^{13}C NMR spectra as well as the MS spectrum are shown in Figures S263–S28.

2.1.7. Nauclealomide C (**7**)

HR-TOF-ESI-MS *m/z* 553.1790 $[M+Na]^+$ (calcd 553.1798 for $C_{26}H_{30}N_2O_{10}Na^+$); ^1H NMR and ^{13}C NMR data in agreement with previous reported data [S7] and listed in Table S3. The ^1H and ^{13}C NMR spectra as well as the MS spectrum are shown in Figures S29–S31.

2.1.8. Paratunamide C (**8**)

HR-TOF-ESI-MS *m/z* 569.1729 $[M+Na]^+$ (calcd 569.1747 for $C_{26}H_{30}N_2O_{11}Na^+$); ^1H NMR and ^{13}C NMR data in agreement with previous reported data [S7] and listed in Table S3. The ^1H and ^{13}C NMR spectra as well as the MS spectrum are shown in Figures S32–S34.

2.1.9. Angustine (**9**)

HR-TOF-ESI-MS *m/z* 314.1269 $[M+H]^+$ (calcd 314.1293 for $C_{20}H_{16}N_3O^+$); ^1H NMR and ^{13}C NMR data in agreement with previous reported data [S8] and listed in Table S4. The ^1H and ^{13}C NMR spectra as well as the MS spectrum are shown in Figures S35–S37.

2.1.10. 3,14-Dihydroangustine (**10**)

HR-TOF-ESI-MS *m/z* 315.1378 $[M]^+$ (calcd 315.1372 for $C_{20}H_{17}N_3O^+$); ^1H NMR and ^{13}C NMR data in agreement with previous reported data [S8] and listed in Table S4. The ^1H and ^{13}C NMR spectra as well as the MS spectrum are shown in Figures S38–S40.

2.1.11. 18,19-Dihydroangustine (**11**)

HR-TOF-ESI-MS *m/z* 316.1439 $[M+H]^+$ (calcd 316.1450 for $C_{20}H_{18}N_3O^+$); ^1H NMR and ^{13}C NMR data in agreement with previous reported data [S8] and listed in Table S4. The ^1H and ^{13}C NMR spectra as well as the MS spectrum are shown in Figures S41–S43.

2.1.12. Angustoline (**12**)

HR-TOF-ESI-MS *m/z* 332.1393 $[M+H]^+$ (calcd 332.1399 for $C_{20}H_{18}N_3O_2^+$); ^1H NMR and ^{13}C NMR data in agreement with previous reported data [S8] and listed in Table S5. The ^1H and ^{13}C NMR spectra as well as the MS spectrum are shown in Figures S44–S46.

2.1.13. 3,14-Dihydroangustoline (**13**)

$[\alpha]_D^{25}$ = -19.4° (c 1.133 mg·mL^{-1}, CH_3OH); HR-TOF-ESI-MS *m/z* 356.1359 $[M+Na]^+$ (calcd 356.1369 for $C_{20}H_{19}N_3O_2Na^+$); ^1H NMR and ^{13}C NMR data in agreement with previous reported data [S8] and listed in Table S5. The ^1H and ^{13}C NMR spectra as well as the MS spectrum are shown in Figures S47–S49.

2.1.14. (*Z*)-2-((2*S*,12b*S*,*Z*)-3-(hydroxymethylene)-4-oxo-1,2,3,4,6,7,12,12b-octahydroindolo[2,3-a]quinolizin-2-yl)but-2-enal (**1a**)

HR-TOF-ESI-MS *m/z* 359.1362 $[M+Na]^+$ (calcd 359.1372 for $C_{20}H_{20}N_2O_3Na^+$); ^1H NMR and ^{13}C NMR data in accordance to previous reported data [S9] and listed in Table S1. The 1H and 13C NMR spectra as well as the MS spectrum are shown in Figures S4–S6.

S3.1. NMR data of the isolated compounds

Table S1. NMR spectroscopic data [ppm] of strictosamide (**1**), 10-hydroxy strictosamide (**2**) and 10-hydroxyvincoside lactam (**3**) recorded in CD_3OD. The relative integral, the multiplicity and the coupling constants [Hz] are provided. In addition, the ^{13}C NMR chemical shifts and multiplicities are given. Numbering of positions is accordance to Figure 2.

	strictosamide (**1**)		10-hydroxy strictosamide (**2**)		10-hydroxyvincoside lactam (**3**)	
position	δ_H (*J* in Hz)	δ_C	δ_H (*J* in Hz)	δ_C	δ_H (*J* in Hz)	δ_C
2	-	134.7, s	-	135.5, s	-	135.3, s
3	5.08 (1H, d, 5.6)	55.0, d	5.04 (1H, dq, 5.9, 1.9)	55.1, d	4.91 (1H, m)	54.8, d
5	4.95 (1H, dd, 12.8, 5.7)	44.6, t	4.93 (1H, dd, 13.0, 5.7)	39.7, t	5.05 (1H, dt, 12.5, 3.5)	41.2, t
	3.12 (1H, td, 12.4, 4.7)		3.09 (1H, td, 12.5, 4.8)		2.93 (1H, m)	
6	2.96 (1H, m)	22.0, t	2.89 (1H, dddd, 14.8, 12.1, 6.0, 2.4)	22.1, t	2.71 (2H, m)	22.0, t
	2.69 (1H, m)		2.60 (1H, m)			
7	-	110.2, s	-	109.6, s	-	108.4, s
8	-	128.6, s	-	129.4, s	-	128.6, s
9	7.39 (1H, d, 8.5)	118.6, d	6.76 (1H, d, 2.3)	103.0, d	6.81 (1H, d, 2.3)	103.3, d
10	7.08 (1H, t, 7.6)	120.1, d	-	151.6, d	-	151.4, d
11	7.00 (1H, t, 7.4)	122.4, d	6.65 (1H, dd, 8.6, 2.4)	112.6, d	7.12 (1H, d, 8.6)	112.2, d
12	7.33 (1H, d, 8.1)	112.2, d	7.15 (1H, d, 8.6)	112.2, d	6.64 (1H, dd, 8.6, 2.3)	112.4, d
13	-	137.6, s	-	132.5, s	-	133.1, s
14	2.47 (1H, ddd, 13.7, 4.7, 2.9)	27.2, t	2.43 (1H, ddd, 14.2, 4.5, 2.1)	27.2, t	2.44 (1H, dt, 13.1, 4.0)	32.6, t
	2.05 (1H, td, 13.8, 5.9)		2.02 (1H, td, 13.9, 6.0)		1.45 (1H, td, 13.3, 11.6)	
15	2.79 (1H, dd, 13.3, 6.0)	24.8, d	2.80 (1H, dtd, 13.1, 4.8, 2.4)	24.9, d	3.25 (1H, m)	27.3, d
16	-	109.1, s	-	109.2, s	-	109.3, s
17	7.38 (1H, d, 2.0)	149.1, d	7.37 (1H, d, 2.3)	149.1, d	7.45 (1H, d, 2.4)	148.9, d
18	5.37 (1H, dd, 17.5, 1.9)	120.5, t	5.36 (1H, dd, 17.1, 1.8)	120.4, t	5.30 (1H, dd, 17.2, 2.0)	120.4, t
	5.32 (1H, dd, 10.3, 1.9)		5.32 (1H, dd, 10.2, 1.9)		5.20 (1H, dd, 10.3, 2.0)	
19	5.66 (1H, dt,17.1, 10.0)	134.2, d	5.65 (1H, dt, 17.1, 10.1)	134.3, d	5.54 (1H, dt, 17.1, 10.0)	133.9, d
20	2.69 (1H, m)	44.7, d	2.68 (1H, ddd, 10.0, 5.7, 1.8)	44.7, d	2.71 (1H, m)	44.5, d
21	5.41 (1H, d,1.8)	98.0, d	5.41 (1H, d, 1.9)	98.0, d	5.51 (1H, d, 1.8)	97.3, d
22	-	167.0, s	-	167.0, s	-	166.0, s
1'	4.58 (1H, d, 7.9)	100.4, d	4.58 (1H, d, 7.9)	100.5, d	4.70 (1H, d, 7.9)	99.6, d
2'	2.96 (1H, m)	74.2, d	2.97 (1H, dd, 9.2, 7.9)	74.3, d	3.21 (1H, m)	74.8, d
3'	3.21 (1H, m)	78.1, d	3.26 (1H, m)	78.2, d	3.39 (1H, t, 9.0)	77.9, d
4'	3.25 (1H, m)	71.2, d	3.19 (1H, dd, 9.7, 8.9)	71.3, d	3.27 (1H, m)	71.5, d
5'	3.25 (1H, m)	77.8, d	3.26 (1H, m)	77.9, d	3.33 (1H, m)	78.3, d
6'	3.86 (1H, dd, 11.9, 2.1)	62.5, t	3.86 (1H, dd, 11.8, 2.2)	62.6, t	3.90 (1H, dd, 12.0, 2.2)	62.6, t
	3.63 (1H, dd, 11.9, 5.8)		3.63 (1H, dd, 11.8, 5.9)		3.68 (1H, dd, 11.9, 5.8)	

Table S2. ^{1}H and ^{13}C NMR spectroscopic data [ppm] of 10-hydroxy-3-*epi*-pumiloside (**4**) and 3-*epi*-pumiloside (**5**) recorded in CD_3OD. The ^{1}H NMR chemical shifts, relative integral, the multiplicity and the coupling constants [Hz] are given. In addition, the ^{13}C NMR chemical shifts and multiplicities are provided. Numbering of positions is accordance to Figure 2.

	10-hydroxy-3-*epi*-pumiloside (**4**)		3-*epi*-pumiloside (**5**)	
position	δ_H (*J* in Hz)	δ_C	δ_H (*J* in Hz)	δ_C
2	-	148.7, s	-	152.4, s
3	4.68 (1H, dd, 12.0, 3.8)	62.3, d	4.93 (1H, dt, 12.7, 3.0)	61.3, d
5	4.83 (1H, d, 14.7)	48.8, d	4.75 (1H, dd, 14.4, 2.4)	49.0, t
	4.61 (1H, d, 14.8)		4.57 (1H, dd, 14.4, 1.3)	
6	-	112.0, s	-	114.8, s
7	-	170.3, s	-	176.1, s
8	-	128.7, s	-	126.5, s
9	7.58 (1H, s)	107.1, d	8.28 (1H, dd, 8.2, 1.4)	126.0, d
10	-	154.4, s	7.43 (1H, t, 8.2)	125.3, d
11	7.08 (1H, dd, 8.9, 2.9)	121.6, d	7.72 (1H, t, 8.5)	133.4, d
12	7.59 (1H, d, 6.7)	127.6, d	7.65 (1H, d, 7.4)	119.5, d
13	-	136.7, s	-	142.1, s
14	2.61 (1H, m)	30.3, t	2.58 (1H, dd, 13.4, 3.8)	29.6, t
	1.98 (1H, m)		2.13 (1H, td, 13.0, 10.5)	
15	3.40 (1H, m)	25.3, d	3.42 (1H, m)	25.1, d
16	-	110.8, s	-	109.9, s
17	7.15 (1H, d, 2.7)	147.0, d	7.18 (1H, d, 2.7)	147.7, d
18	5.49 (1H, dd, 17.2, 2.1)	121.0, t	5.55 (1H, dd, 26.6, 1.9)	121.4, t
	5.37 (1H, dd, 10.4, 2.0)		5.41 (1H, dd, 10.3, 2.0)	
19	5.88 (1H, dt, 17.2, 10.0)	133.6, d	5.87 (1H, dt,17.1, 10.0)	133.5, d
20	2.69 (1H, dt, 7.3, 2.4)	45.9, d	2.70 (1H, ddd, 9.8, 5.1, 1.6)	46.0, d
21	5.49 (1H, d, 1.6)	97.4, d	5.51 (1H, d,1.7)	97.4, d
22	-	167.9, s	-	167.5, s
1'	4.73 (1H, d, 7.9)	99.7, d	4.73 (1H, d, 7.9)	99.8, d
2'	3.21 (1H, m)	74.8, d	3.20 (1H, dd, 9.2, 7.9)	74.8, d
3'	3.38 (1H, m)	77.9, d	3.39 (1H, t, 9.0)	77.9, d
4'	3.29 (1H, m)	71.5, d	3.28 (1H, m)	71.5, d
5'	3.33 (1H, m)	78.3, d	3.33 (1H, m)	78.3, d
6'	3.90 (1H, m)	62.6, t	3.91 (1H, dd, 12.0, 2.2)	62.6, t
	3.68 (1H, dd, 12.0, 5.7)		3.78 (1H, dt, 9.5, 2.7)	

Table S3. NMR spectroscopic data [ppm] of nauclealomide B (**6**), nauclealomide C (**7**) and paratunamide C (**8**) recorded in CD_3OD. The relative integral, the multiplicity and the coupling constants [Hz] are provided. In addition, the ^{13}C NMR chemical shifts and multiplicities are given. Numbering of positions is accordance to Figure 2.

	Nauclealomide B (**6**)		Nauclealomide C (**7**)		Paratunamide C (**8**)	
position	δ_H (*J* in Hz)	δ_C	δ_H (*J* in Hz)	δ_C	δ_H (*J* in Hz)	δ_C
2	-	179.5, s	-	177.9, s	-	181.3, s
3	6.09 (1H, dd, 9.5, 4.6)	80.4, d	5.76 (1H, dd, 3.9, 2.0)	81.3, d	-	173.6, s
5	4.47 (1H, dt,13.4, 4.7)	36.0, t	4.75 (1H, ddd, 13.7, 5.6, 2.8)	39.7, t	3.84 (1H, m)	35.8, t
	3.76 (1H, m)		3.59 (1H, td, 13.0, 3.4)		3.73 (1H, m)	
6	2.2 (1H, m)	31.7, t	2.19 (1H, td, 13.0, 5.6)	31.3, t	2.20 (2H, m)	36.0, t
	2.07 (1H, dt, 14.1, 4.1)		1.70 (1H, dt, 13.6, 3.1)			
7	-	76.3, s	-	79.4, s	-	76.3, s
8	-	131.5, s	-	131.6, s	-	132.3, s
9	7.30 (1H, d, 7.5)	125.1, d	7.86 (1H, d, 7.5)	126.6, d	7.34 (1H, d, 7.3)	125.1, d
10	7.04 (1H, td, 7.6, 1.0)	123.8, d	7.09 (1H, td, 7.6, 1.1)	123.7, d	7.06 (1H, t, 7.4)	123.7, d
11	7.27 (1H, m)	131.2, d	7.33 (1H, td, 7.8, 1.1)	131.6, d	7.26 (1H, td, 7.7, 1.2)	130.7, d
12	6.87 (1H, d, 7.7)	111.1, d	6.95 (1H, d, 7.7)	111.9, d	6.87 (1H, d, 7.8)	111.4, d
13	-	142.4, s	-	142.6, s	-	142.9, s
14	1.49 (1H, td, 13.6, 9.6)	32.3, t	1.82 (1H, td, 14.0, 3.9)	29.9, t	2.42 (1H, dd, 15.9, 5.4)	34.7, t
	2.00 (1H, dt,12.9, 4.3)		1.75 (1H, m)		2.24 (1H, d, 5.3)	
15	3.06 (1H, m)	24.4, d	3.36 (1H, m)	23.5, d	3.17 (1H, m)	24.4, d
16	-	109.1, s	-	109.2, s	-	107.8, s
17	7.42 (1H, d, 2.4)	149.1, d	7.44 (1H, d, 2.4)	149.5, d	7.54 (1H, d, 2.4)	152.4, d
18	5.27 (2H, m)	120.7, t	5.26 (2H, m)	120.6, t	5.32 (2H, m)	121.7, t
19	5.58 (1H, dt,17.2, 10.0)	133.8, d	5.58 (1H, dt, 17.2, 10.1)	134.1, d	5.50 (1H, m)	133.2, d
20	2.64 (1H, dd, 9.7, 5.8)	44.4, d	2.60 (1H, m)	44.2, d	2.67 (1H, dd, 9.7, 5.5)	43.9, d
21	5.50 (1H, d, 1.9)	97.3, d	5.48 (1H, d, 1.8)	97.5, d	5.56 (1H, d, 1.9)	97.8, d
22	-	165.7, s	-	166.2, s	-	167.4, s
1'	4.66 (1H, d, 7.9)	99.5, d	4.67 (1H, d, 7.9)	99.9, d	4.66 (1H, d, 7.9)	99.7, d
2'	3.18 (1H, dd,9.2, 7.9)	74.7, d	3.24 (1H, dd, 9.2, 7.8)	74.6, d	3.20 (1H, m)	74.6, d
3'	3.37 (1H, t, 8.8)	77.8, d	3.39 (1H, t, 8.8)	77.7, d	3.36 (1H, m)	77.7, d
4'	3.30 (1H, m)	71.5, d	3.29 (1H, m)	71.5, d	3.27 (1H, m)	71.5, d
5'	3.31 (1H, m)	78.3, d	3.31 (1H, m)	78.3, d	3.31 (1H, m)	78.3, d
6'	3.89 (1H, dd, 12.0, 2.1)	62.6, t	3.89 (1H, dd, 12.0, 2.1)	62.7, t	3.89 (1H, m)	62.6, t
	3.66 (1H, dd, 12.0, 5.6)		3.66 (1H, dd, 12.0, 5.7)		3.65 (1H, m)	

Table S4. NMR spectroscopic data [ppm] of angustine (**9**), 3,14-dihydroangustine (**10**), and 18,19-dihydroangustine (**11**), recorded in CD_3OD. The relative integral, the multiplicity and the coupling constants [Hz] are provided. In addition, the ^{13}C NMR chemical shifts and multiplicities are given. Numbering of positions is accordance to Figure 2.

	Angustine (**9**)		3,14-Dihydroangustine (**10**)		18,19-Dihydroangustine (**11**)	
position	δ_H (*J* in Hz)	δ_C	δ_H (*J* in Hz)	δ_C	δ_H (*J* in Hz)	δ_C
2	-	128.7, s	5.09 (1H, br)	133.0, s	-	127.6, s
3	-	139.1, s	-	52.7, d	-	139.5, s
5	4.50 (2H, t, 6.7)	42.2, t	5.14 (1H, m)<br>3.09 (1H, td, 12.4, 4.3)	40.8, t	4.67 (2H, t, 6.7)	42.6, t
6	3.17 (2H, t, 6.7)	20.5, t	2.93 (2H, m)	21.8, t	3.34 (2H, t, 6.7)	20.7, t
7	-	116.9, s	-	109.4, s	-	118.4, s
8	-	127.0, s	-	126.1, s	-	127.2, s
9	7.59 (1H, d, 8.0)	120.6, d	7.49 (1H, dd, 7.8, 1.2)	119.1, d	7.77 (1H, d, 8.0)	120.6, d
10	7.10 (1H, t, 7.5)	121.3, d	7.02 (1H, m)	120.3, d	7.26 (1H, t, 7.5)	121.0, d
11	7.26 (1H, t, 7.4)	126.0, d	7.12 (1H, m)	122.9, d	7.42 (1H, t, 7.6)	126.1, d
12	7.42 (1H, d, 8.2)	112.9, d	7.35 (1H, dt, 8.2, 1.0)	112.2, d	7.59 (1H, d, 8.3)	112.9, d
13	-	140.6, s	-	138.5, s	-	140.0, s
14	7.20 (1H, s)	95.2, d	3.89 (1H, dd, 16.7, 4.1)<br>2.93 (1H, m)	32.3, t	7.33 (1H, s)	95.6, d
15	-	141.5, s	-	145.1, s	-	143.4, s
16	-	120.8, s	-	127.8, d	-	120.2, s
17	9.26 (1H, s)	150.6, d	9.03 (1H, s)	148.9, d	9.40 (1H, s)	149.7, d
18	5.95 (1H, d, 17.4)<br>5.63 (1H, d, 11.0)	120.1, t	5.91 (1H, dd, 17.5, 0.9)<br>5.63 (1H, dd, 11.2, 0.9)	120.8, t	1.54 (3H, t, 7.6)	15.5, t
19	7.26 (1H, m)	131.3, d	7.07 (1H, m)	131.4, d	3.16 (2H, q, 7.5)	23.9, t
20	-	129.7, s	-	133.3, s	-	130.0, s
21	8.71 (1H, s)	147.9, d	8.79 (1H, s)	150.4, d	8.61 (1H, s)	149.6, d
22	-	163.5, s	-	165.1, s	-	164.6, s

Table S5. NMR spectroscopic data [ppm] of angustoline (**12**), 3,14-dihydro angustoline (**13**, main isomer) and **1a** recorded in CD_3OD. The relative integral, the multiplicity and the coupling constants [Hz] are provided. In addition, the ^{13}C NMR chemical shifts and multiplicities are given. Numbering of positions is accordance to Figure 2.

	Angustoline (**12**)		3,14-Dihydro angustoline (**13**)		Compound **1a**	
position	δ_H (*J* in Hz)	δ_C	δ_H (*J* in Hz)	δ_C	δ_H (*J* in Hz)	δ_C
2	-	128.7, s	-	133.3, s	-	133.7, s
3	-	138.8, s	5.00 (1H, d 12.6)	52.6, d	5.03 (1H, d, 8.0)	55.3, d
5	4.56 (2H, t, 6.7)	42.1, t	3.04 (1H, td, 12.2, 4.1)<br>5.11 (1H, m)	40.7, t	4.95 (1H, m)<br>3.08 (1H, m)	44.6, t
6	3.13 (2H, t, 6.7)	20.5, t	2.90 (2H, m)	21.8, t	2.94 (1H, ddd 12.2, 5.7, 2.9)<br>2.76 (1H, m)	22.6, t
7	-	116.8, s	-	109.4, s	-	109.3, s
8	-	127.0, s	-	127.8, s	-	110.6, s
9	7.57 (1H, d, 8.0)	120.6, d	7.46 (1H, d, 7.8)	119.1, d	7.32 (1H, d, 8.3)	112.2, d
10	7.07 (1H, t, 7.8)	121.3, d	7.00 (1H, t, 7.5)	120.2, d	7.00 (1H, m)	120.1, d
11	7.25 (1H, t, 7.7)	126.0, d	7.10 (1H, t, 8.2)	122.9, d	7.08 (1H, m)	122.4, d
12	7.41 (1H, d, 8.3)	112.8, d	7.34 (1H, d, 9.1)	112.2, d	7. 25 (1H, d, 8.1)	118.6, d
13	-	140.6, s	-	139.7, s	-	136.7, s
14	7.16 (1H, s)	95.3, d	2.79 (1H, m)<br>3.88 (1H, ddd, 16.0, 15.5, 4.0)	31.9, t	2.43 (1H, m)<br>1.83 (1H, m)	35.5, t
15	-	141.2, s	-	145.3, s	3.12 (1H, m)	31.2, d
16	-	120.7, s	-	126.1, s	-	100.0, s
17	9.26 (1H, s)	150.5, d	9.202 (1H, s)	150.5, d	7.39 (1H, m)	118.5, d
18	1.62 (3H, d, 6.5)	24.9, t	1.63 (3H, d, 6.5)	24.4, q	2.05 (3H, dd, 7.3, 0.7)	15.3, q
19	5.41 (1H, q, 6.5)	66.2, d	5.17 (1H, q, 6.5)	66.2, d	6.73 (1H, q, 7.2)	152.2, d
20	-	136.3, s	-	138.5, s	-	145.9, s
21	8.71 (1H, s)	147.6, d	8.74 (1H, s)	149.0, d	9.35 (1H, s)	196.5, d
22	-	163.6, s	-	165.0, s	-	167.7, s

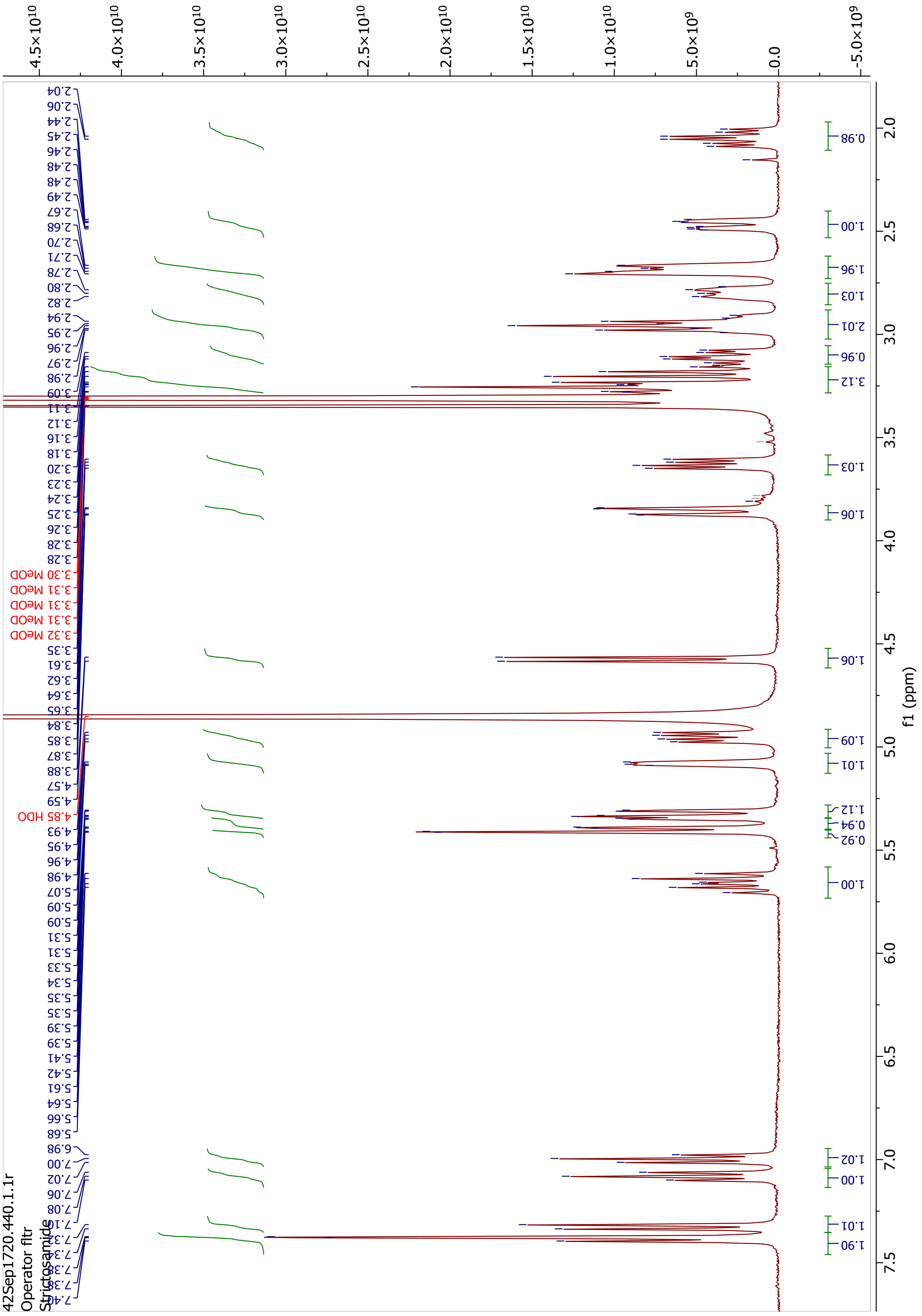

Figure S1. ^{1}H NMR of strictosamide (**1**) in CD_3OD.

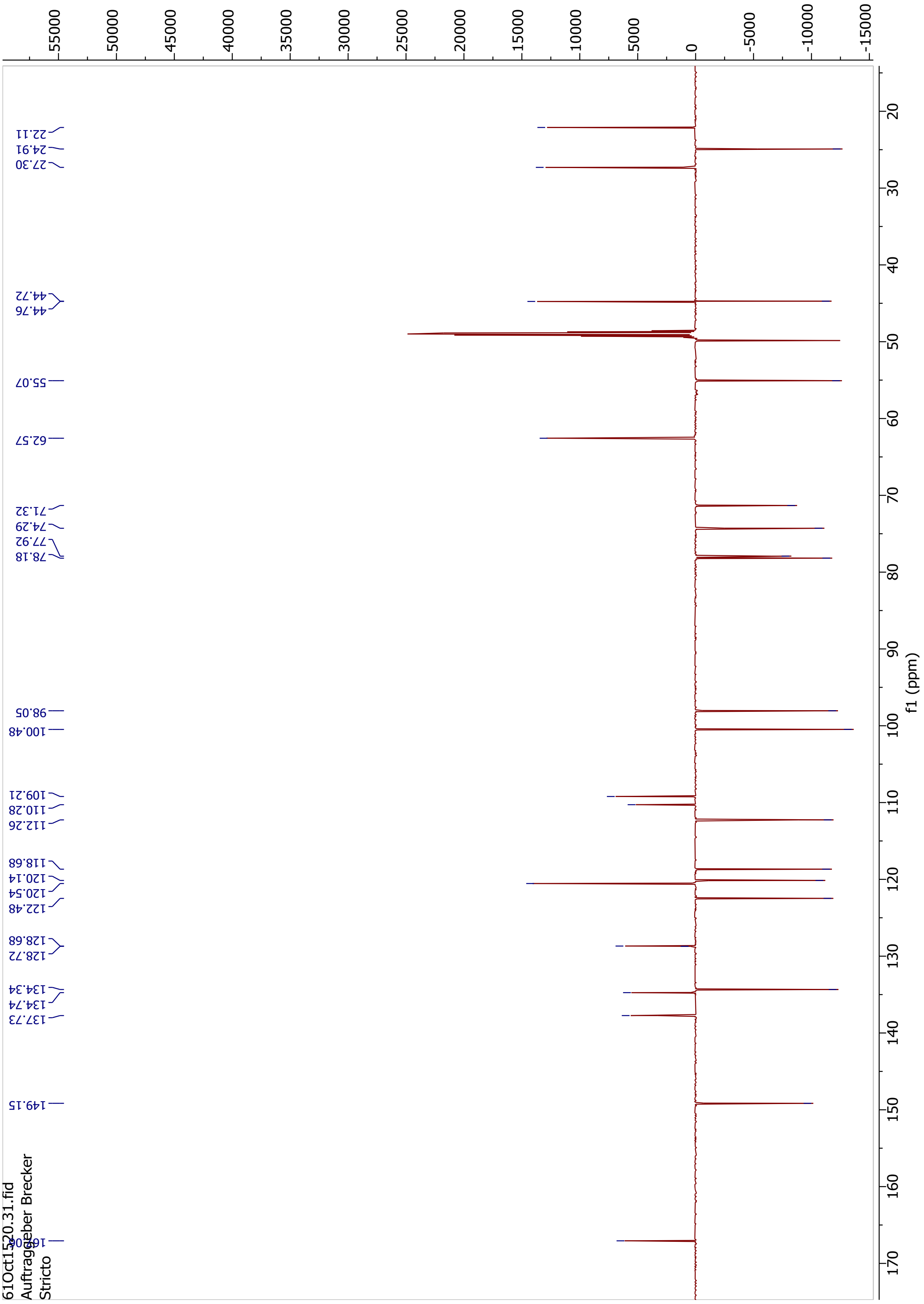

Figure S2. ^{13}C NMR of strictosamide (**1**) in CD_3OD.

Generic Display Report

Analysis Info

Analysis Name	E:\Data\MS_MessService\72381000001.d	Acquisition Date	8/17/2020 12:53:03 PM
Method	tune_low_MS_Service_08_20.m	Operator	msc
Sample Name	Stricto	Instrument	maXis
Comment	Weerasak Songoen/Brecker<br>Ergebnis +/- 5ppm<br>ACN / MeOH + 1% H2O		

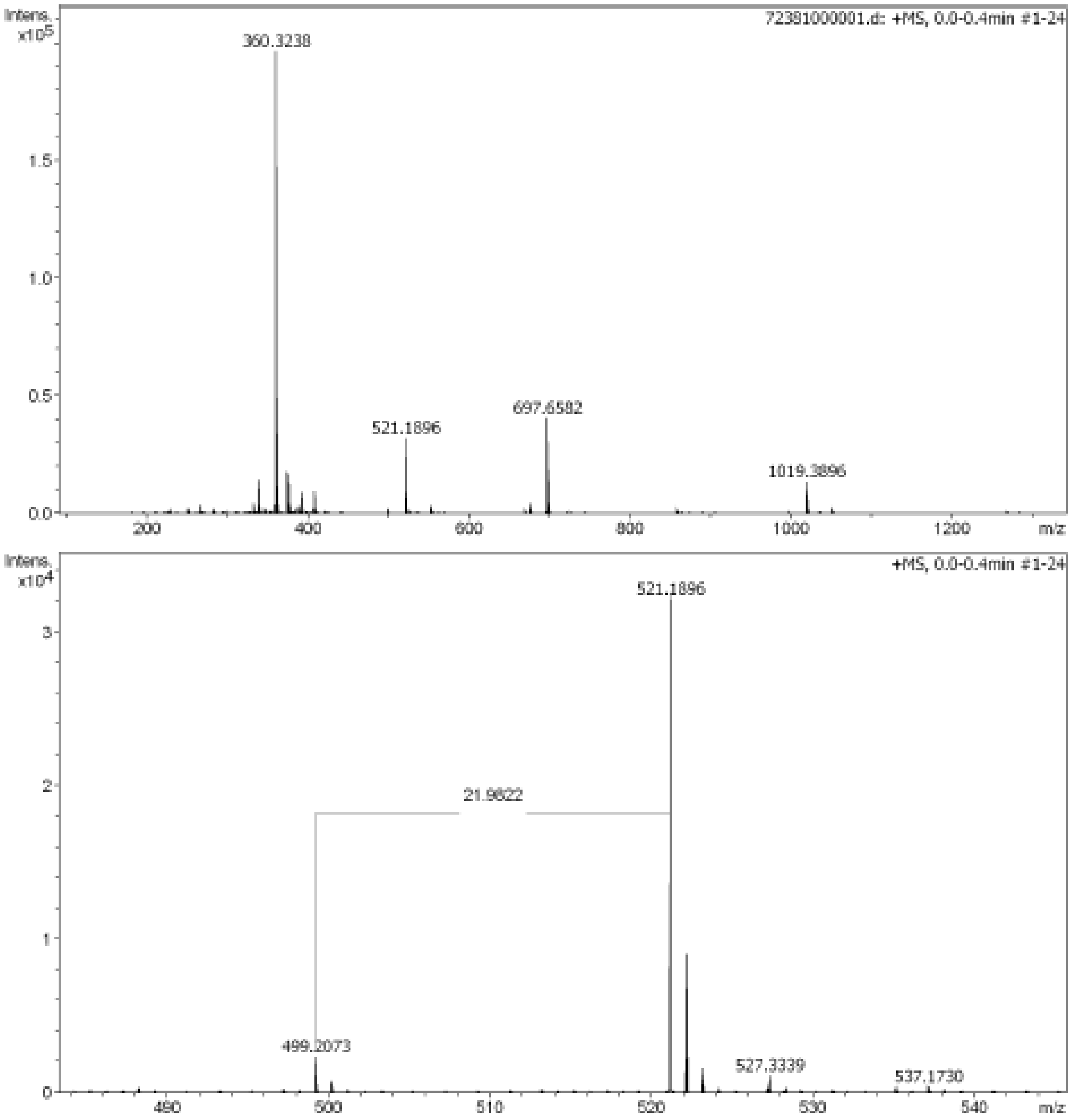

Bruker Compass DataAnalysis 4.1 printed: 8/17/2020 2:11:17 PM by: msc Page 1 of 1

Figure S3. Mass spectrum of strictosamide (**1**).

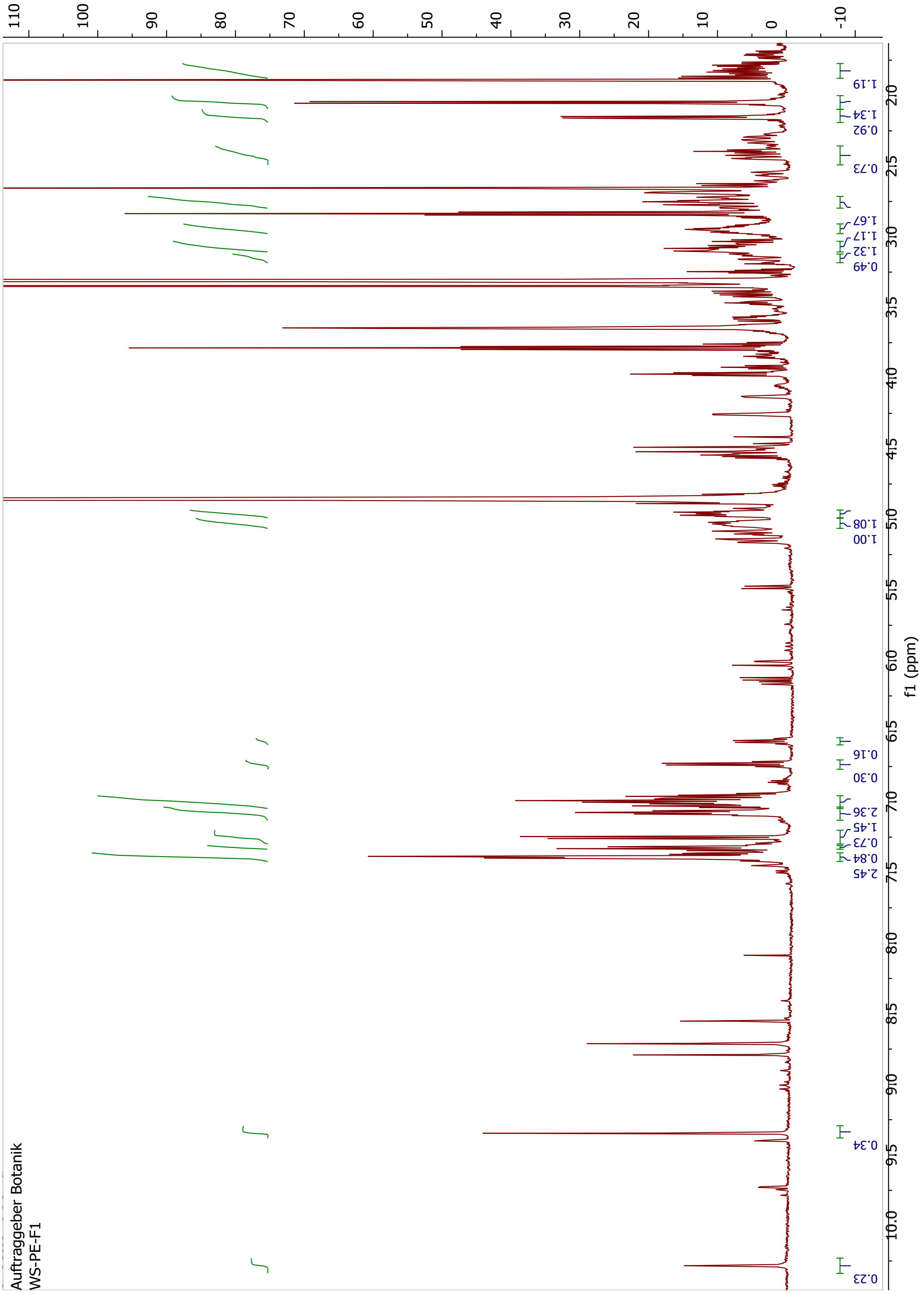

Figure S4. ^{1}H NMR of **1a** in CD_3OD.

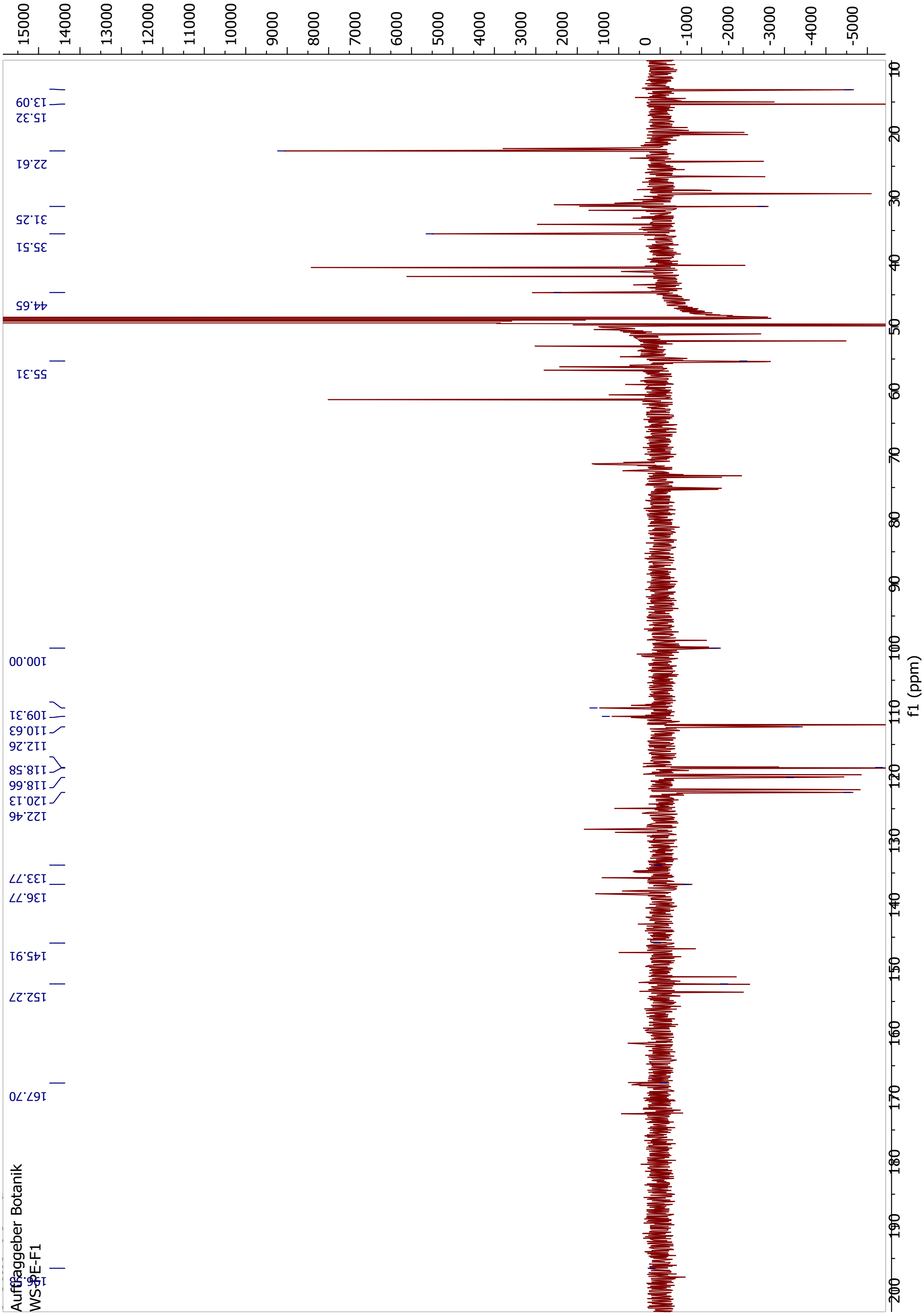

Figure S5. ^{13}C NMR of **1a** in CD_3OD.

Generic Display Report

**Analysis Info**		Acquisition Date	10/9/2020 1:28:23 PM
Analysis Name	E:\Data\MS_MessService\73878000001.d		
Method	tune_low_MS_Service_10_20.m	Operator	msc
Sample Name	WS_PE_F1	Instrument	maXis
Comment	Weerasak / Brecker<br>ACN/MeOH + 1% H2O<br>Ergebnis +/- 5 ppm		

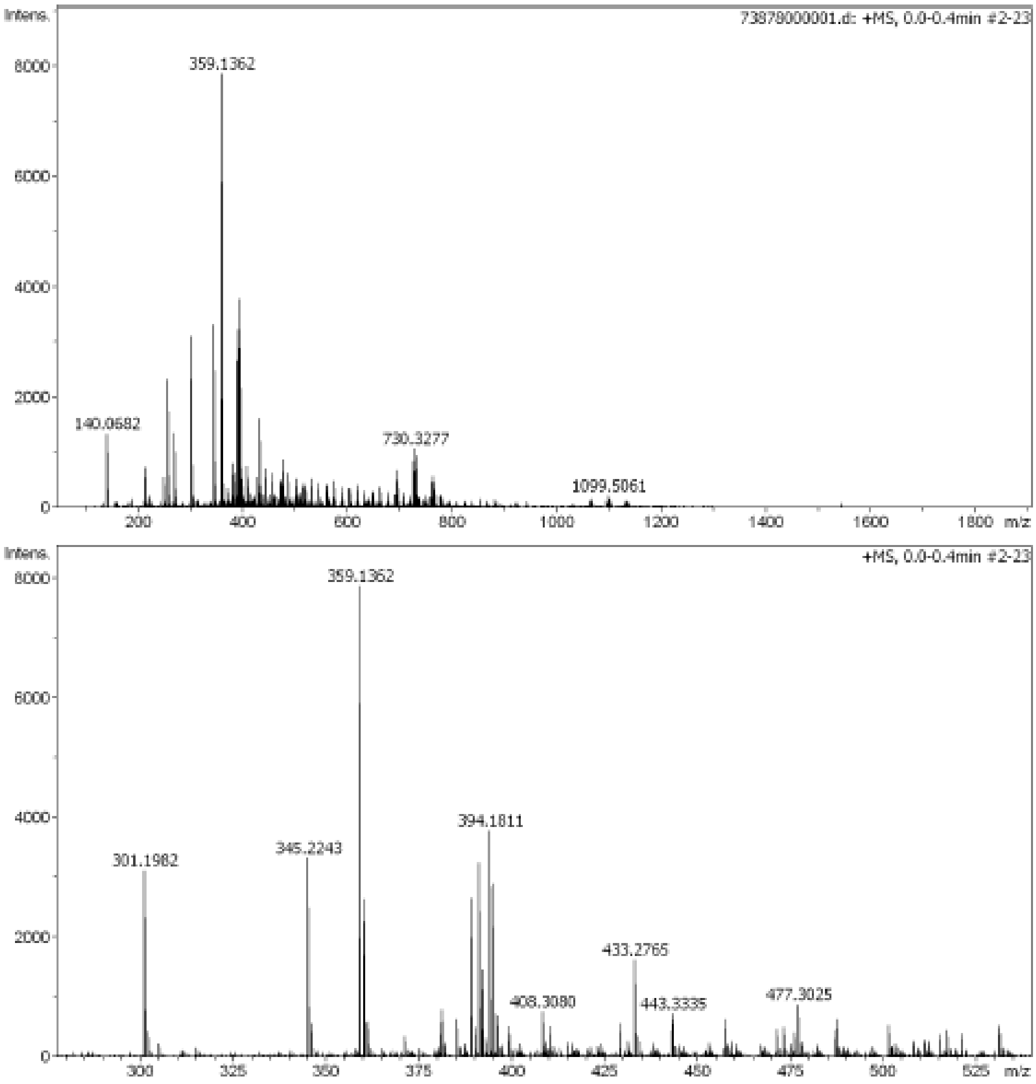

Bruker Compass DataAnalysis 4.1 printed: 10/9/2020 1:30:16 PM by: msc Page 1 of 1

Figure S6. Mass spectrum of **1a**.

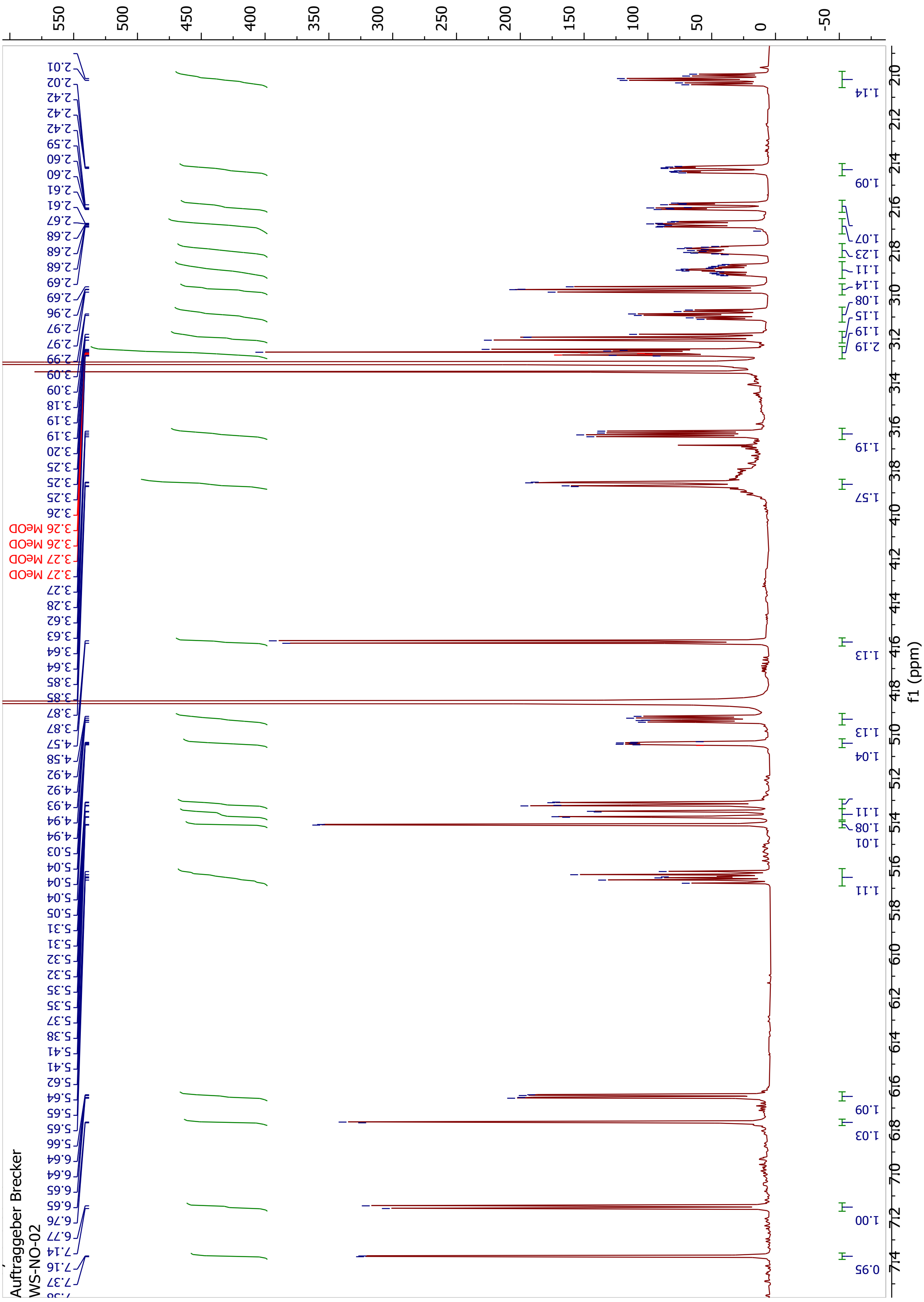

Figure S7. ^{1}H NMR of 10-hydroxy strictosamide (**2**) in CD_3OD.

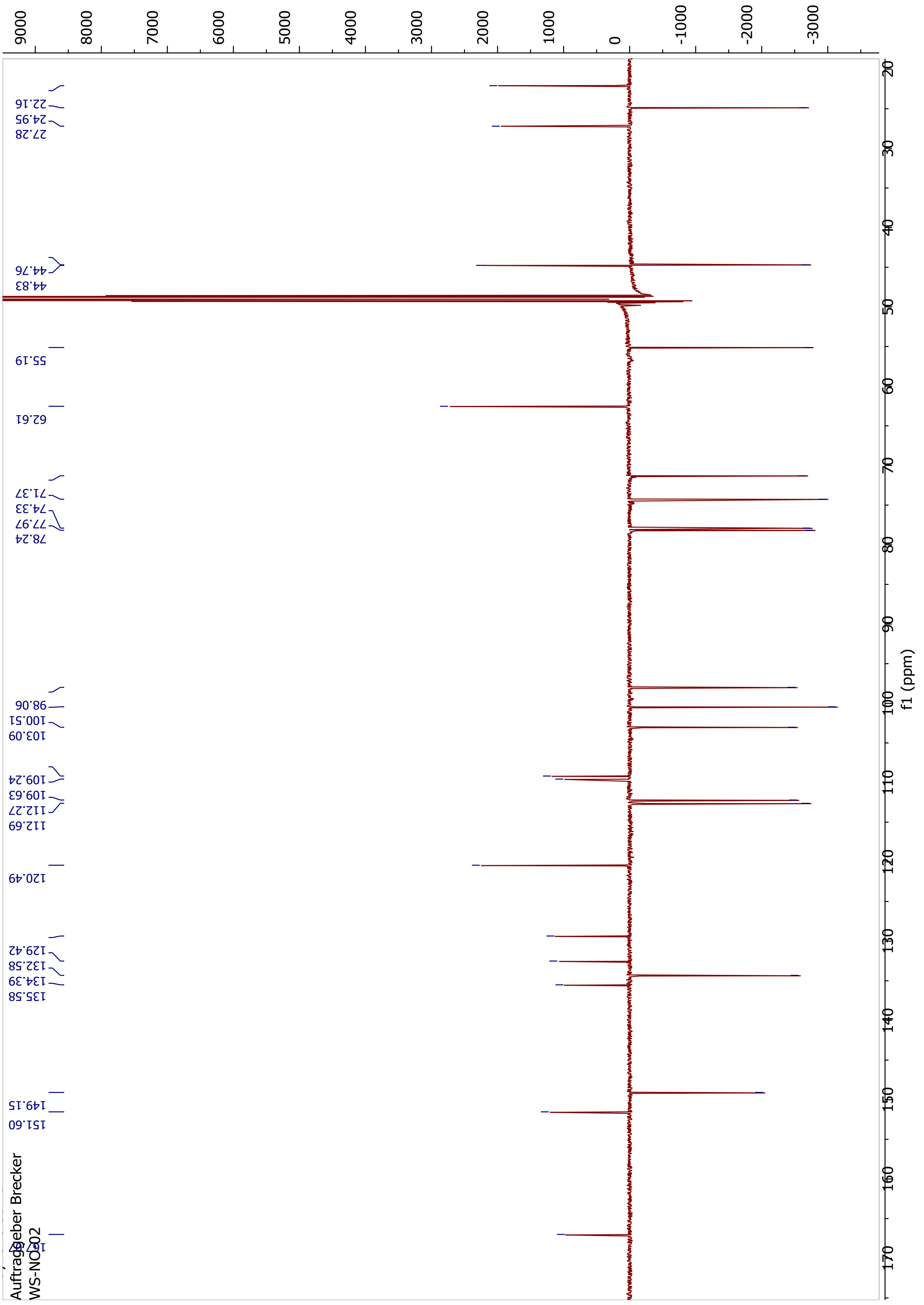

Figure S8. ^{13}C NMR of 10-hydroxy strictosamide (**2**) in CD_3OD.

Generic Display Report

Analysis Info

Analysis Name	E:\Data\MS_MessService\70297000001.d	Acquisition Date	5/19/2020 1:56:54 PM
Method	tune_low_MS_Service_05_20.m	Operator	msc
Sample Name	WS-NO-02	Instrument	maXis
Comment	Fueloep / Brecker<br>Ergebnis +/- 5 ppm<br>ACN + MeOH + 1 %H2O		

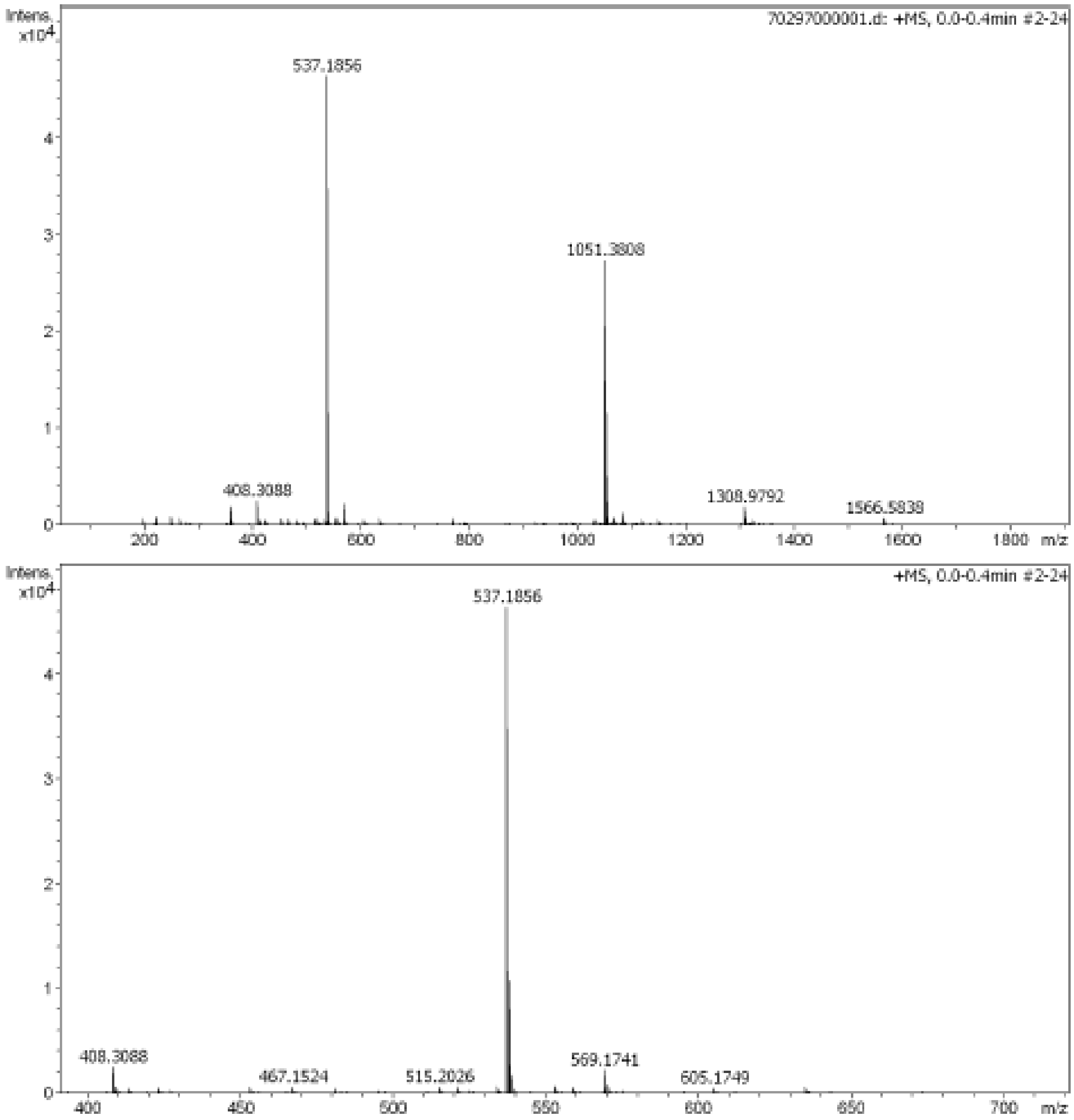

Bruker Compass DataAnalysis 4.1 printed: 5/19/2020 2:10:46 PM by: msc Page 1 of 1

Figure S9. Mass spectrum of 10-hydroxy strictosamide (**2**).

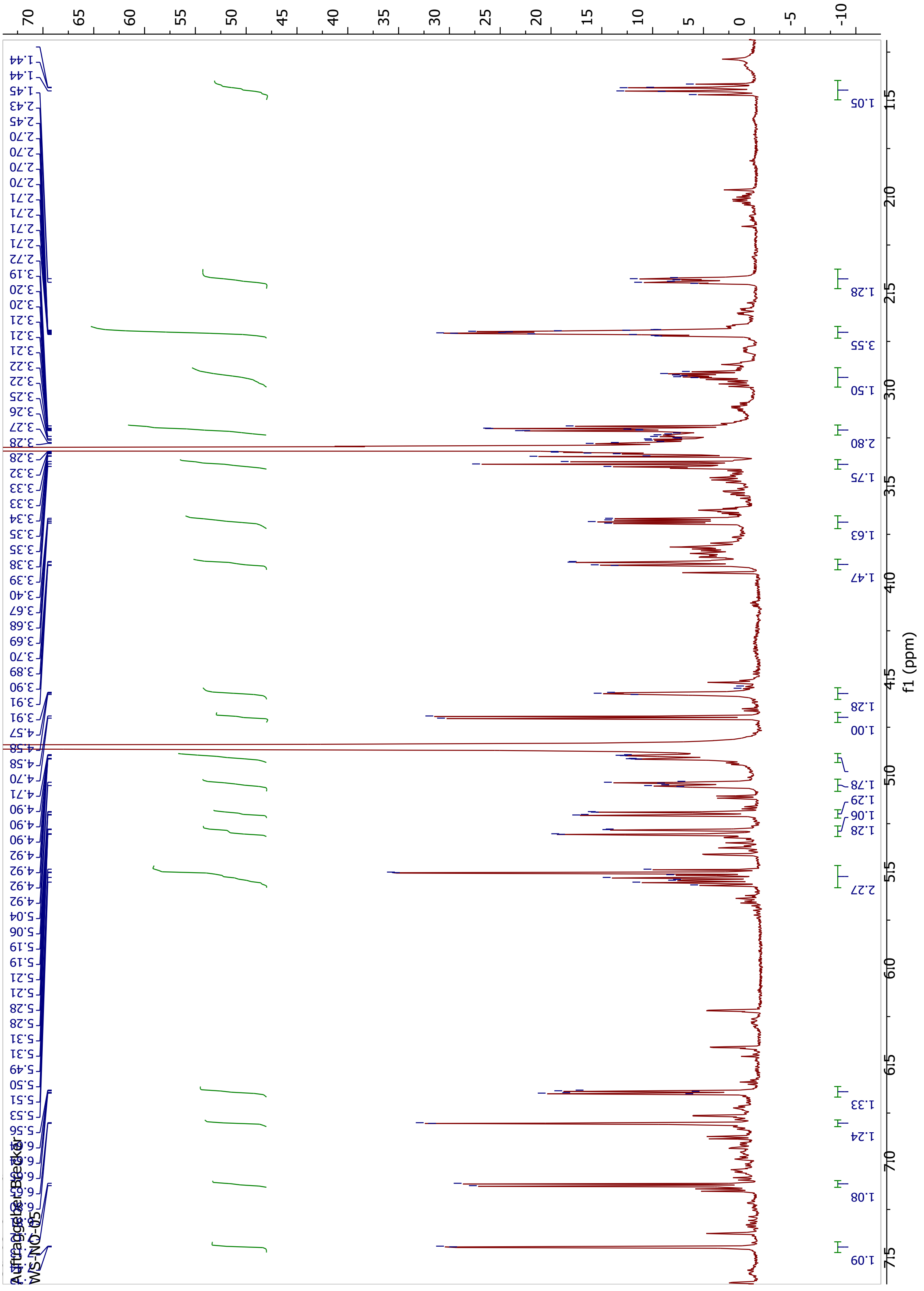

Figure S10. ^{1}H NMR of 10-hydroxyvincoside lactam (**3**) in CD_3OD.

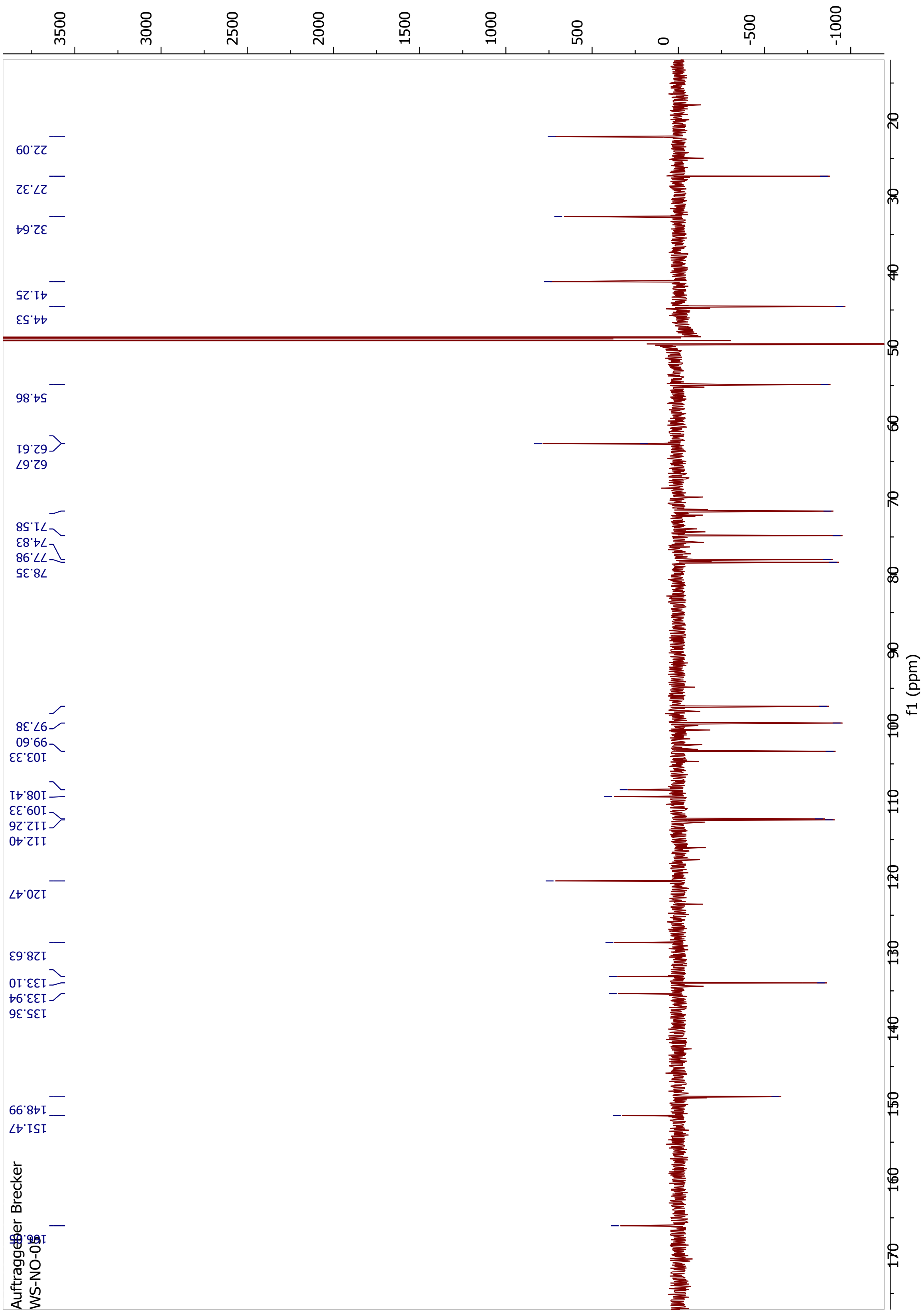

Figure S11. ^{13}C NMR of 10-hydroxyvincoside lactam (**3**) in CD_3OD.

Generic Display Report

**Analysis Info**		Acquisition Date	5/27/2020 12:40:37 PM
Analysis Name	E:\Data\MS_MessService\70469000001.d		
Method	tune_low_MS_Service_05_20.m	Operator	msc
Sample Name	WS-NO-05	Instrument	maXis
Comment	Fülöp / Org.Chem<br>ACN/MeOH + 1 % H2O<br>Ergebnis +/- 5 ppm		

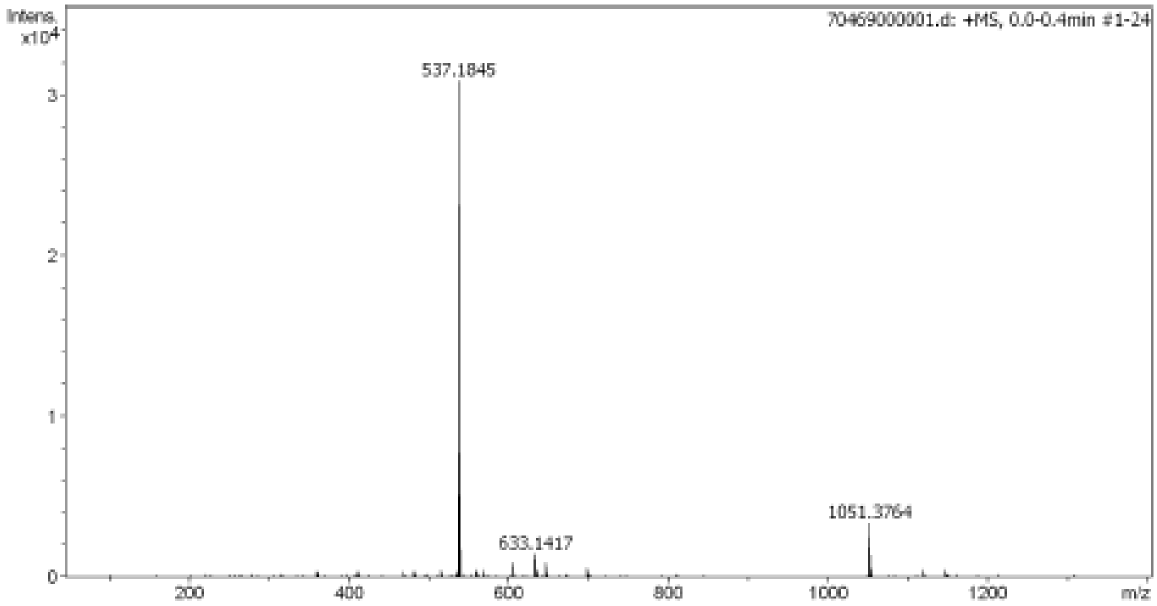

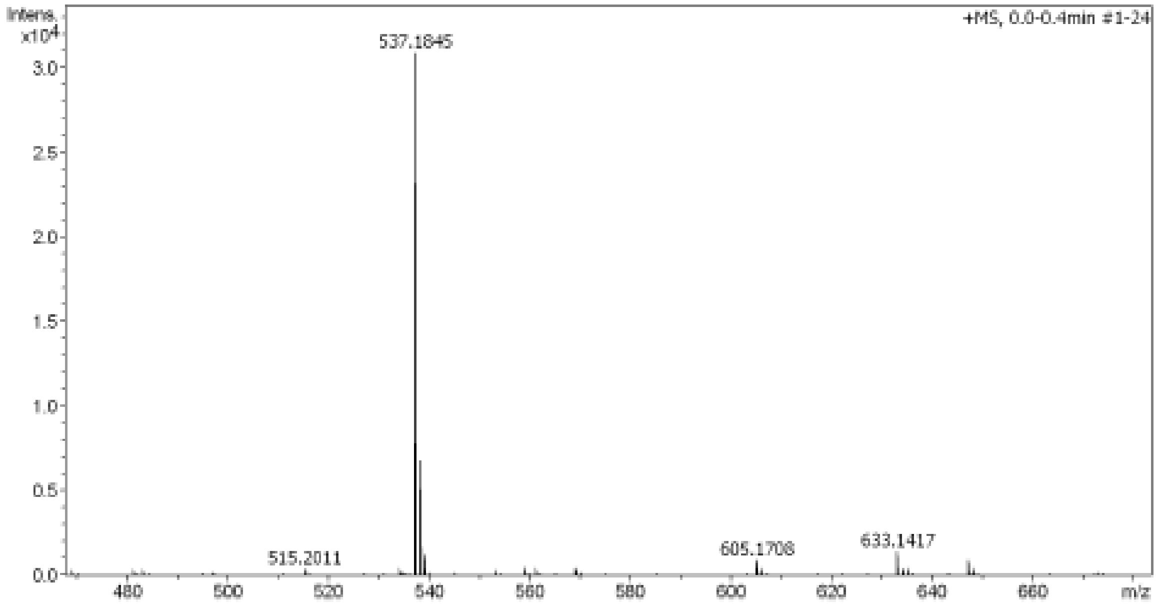

Bruker Compass DataAnalysis 4.1 printed: 5/27/2020 12:47:56 PM by: msc Page 1 of 1

Figure S12. Mass spectrum of 10-hydroxyvincoside lactam (**3**).

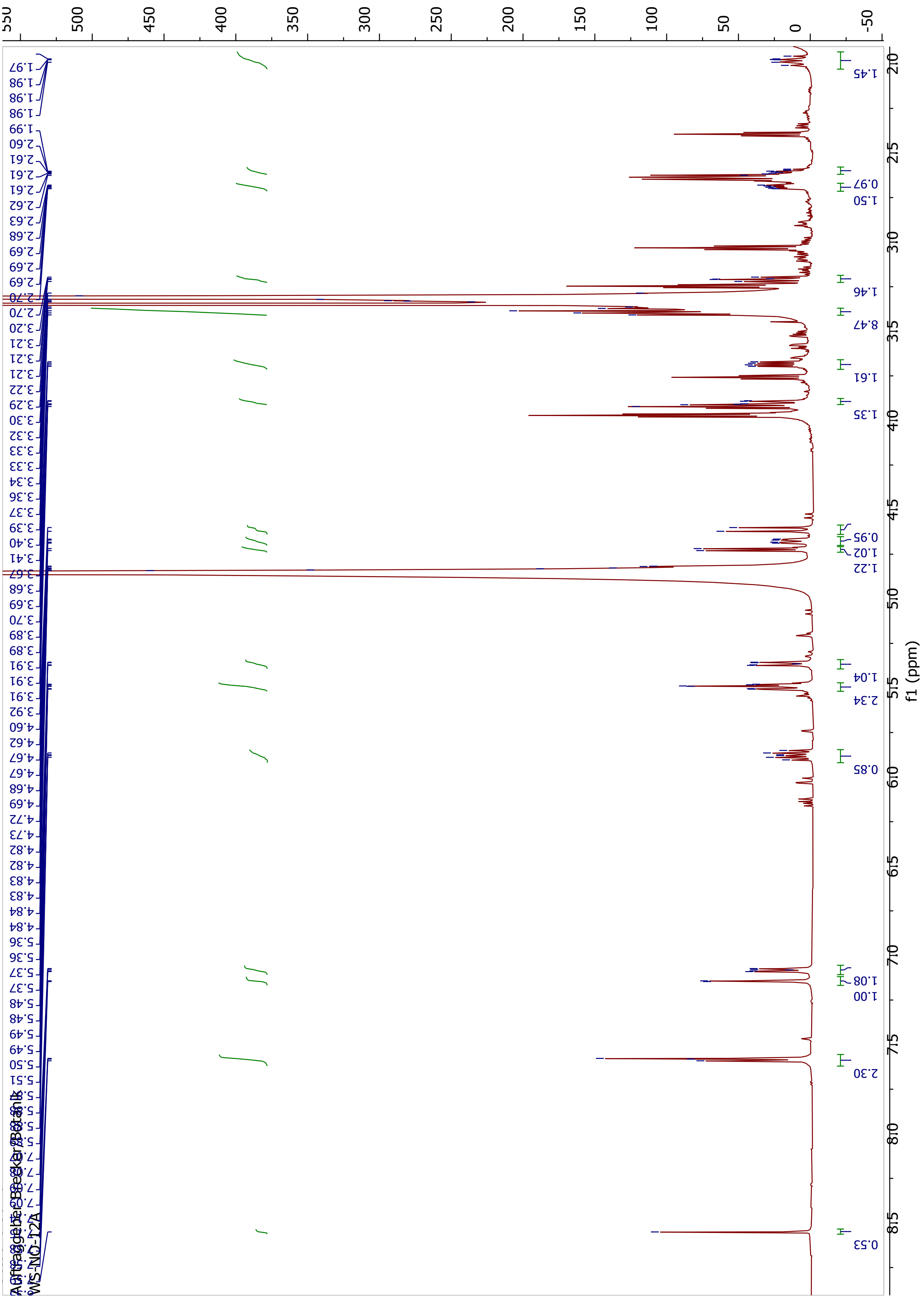

Figure S13. ^{1}H NMR of 10-hydroxy-3-*epi*-pumiloside (**4**) in CD_3OD.

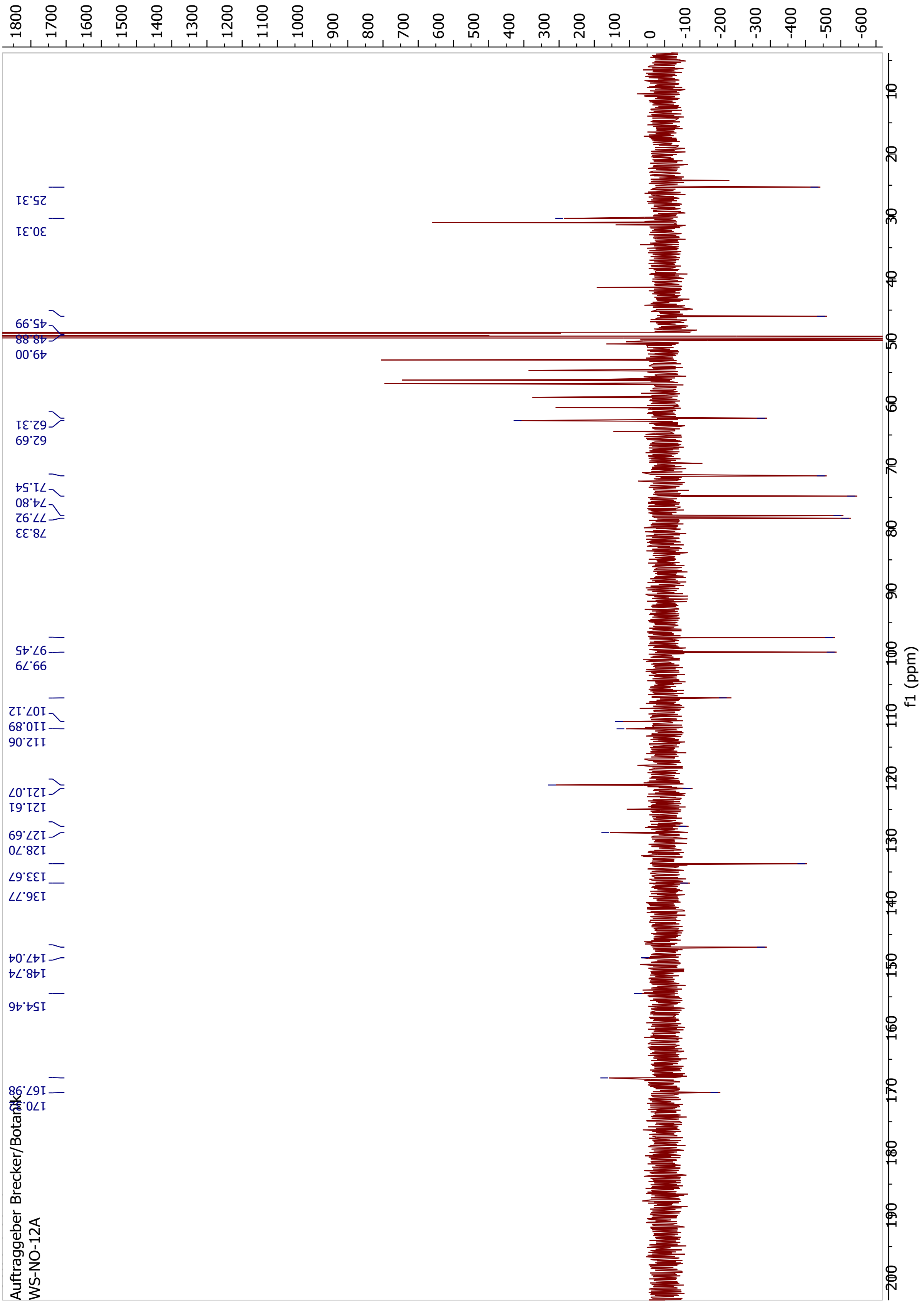

Figure S14. ^{13}C NMR of 10-hydroxy-3-*epi*-pumiloside (**4**) in CD_3OD.

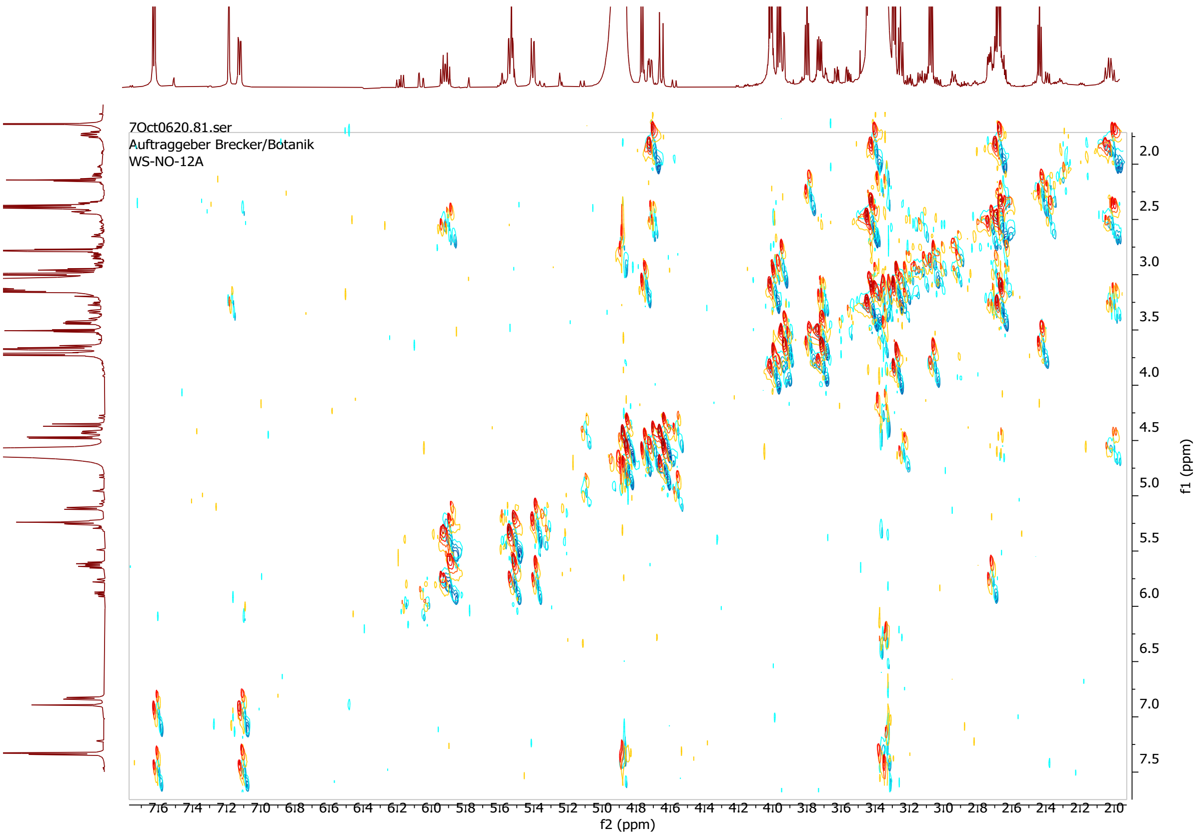

Figure S15. COSY of 10-hydroxy-3-*epi*-pumiloside (**4**) in CD_3OD.

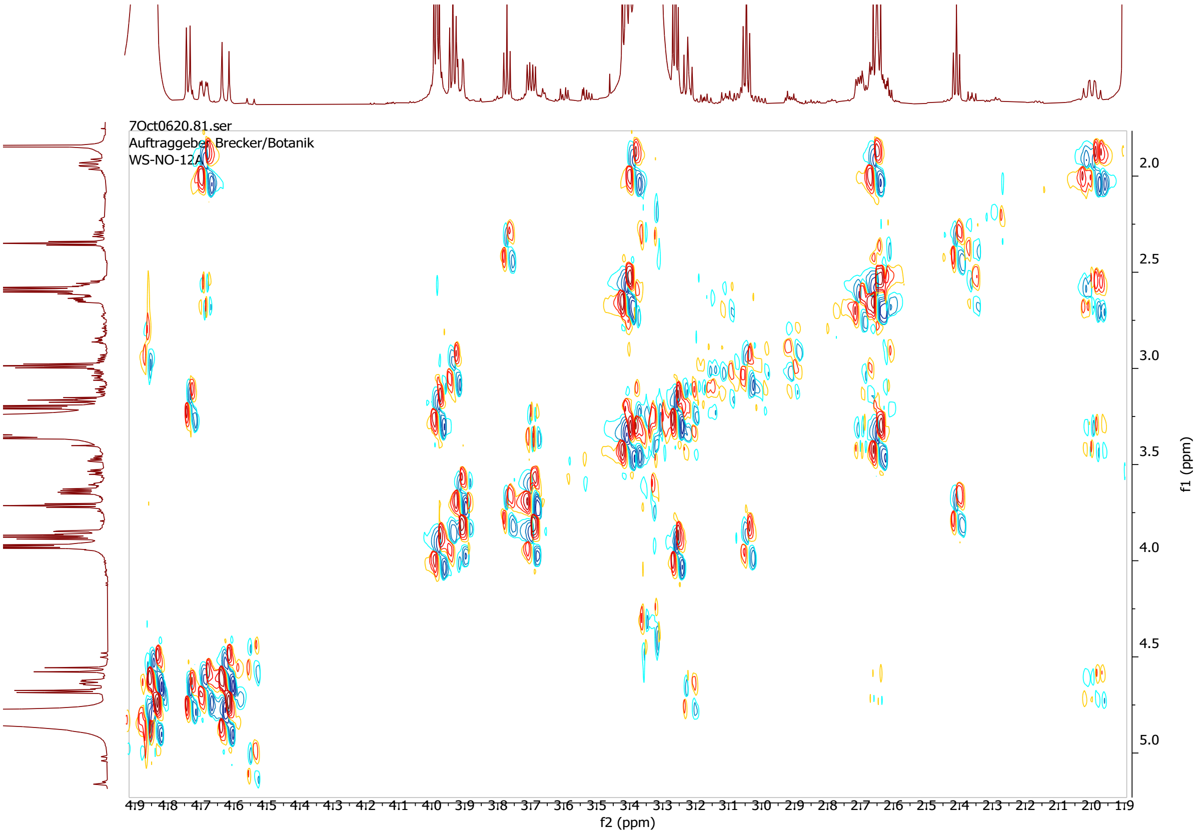

Figure S16. Zoom COSY of 10-hydroxy-3-*epi*-pumiloside (**4**) in CD_3OD.

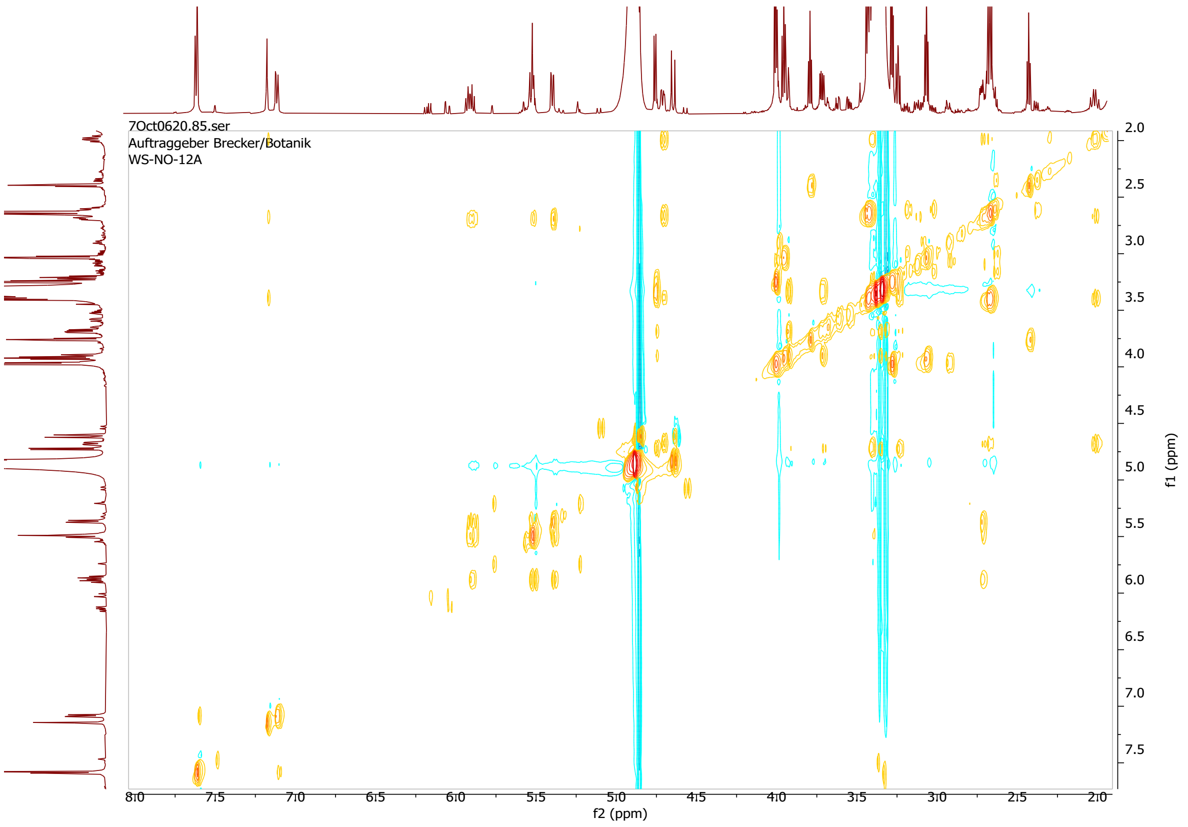

Figure S17. TOCSY of 10-hydroxy-3-*epi*-pumiloside (**4**) in CD3OD.

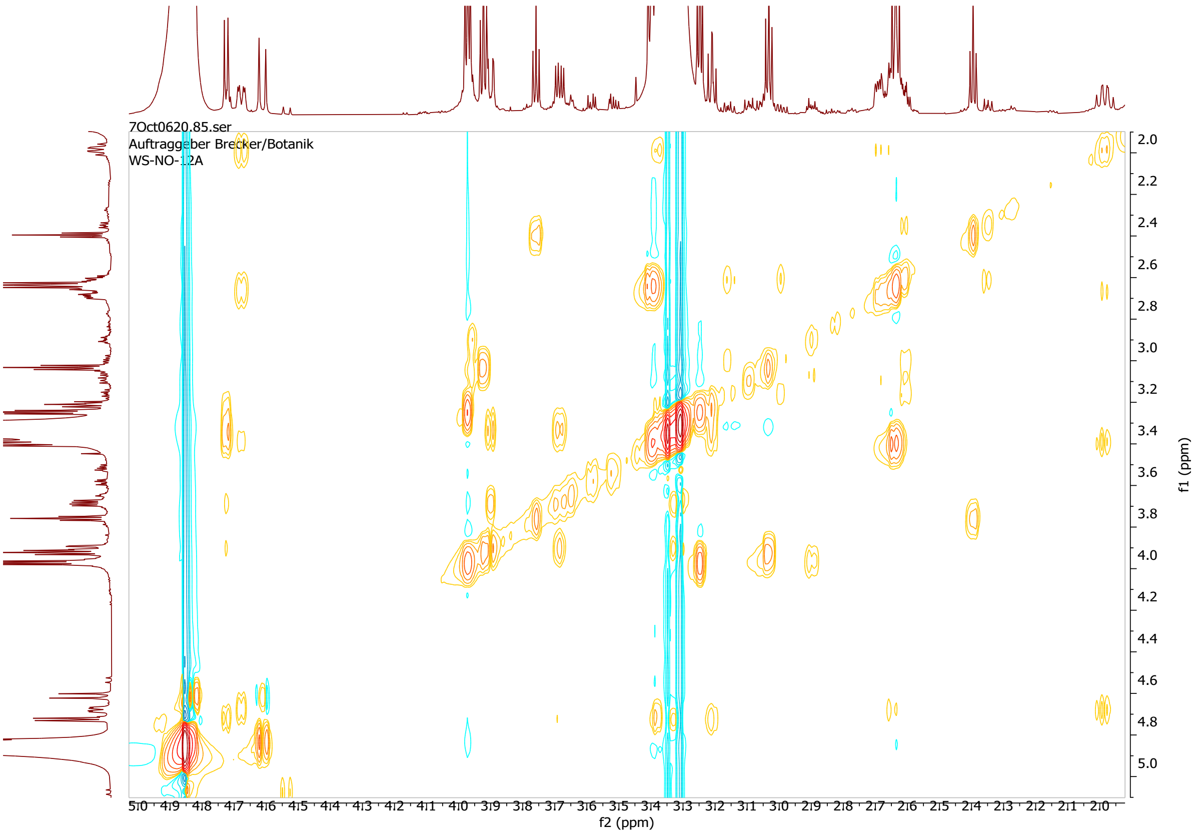

Figure S18. Zoom TOCSY of 10-hydroxy-3-*epi*-pumiloside (**4**) in CD3OD.

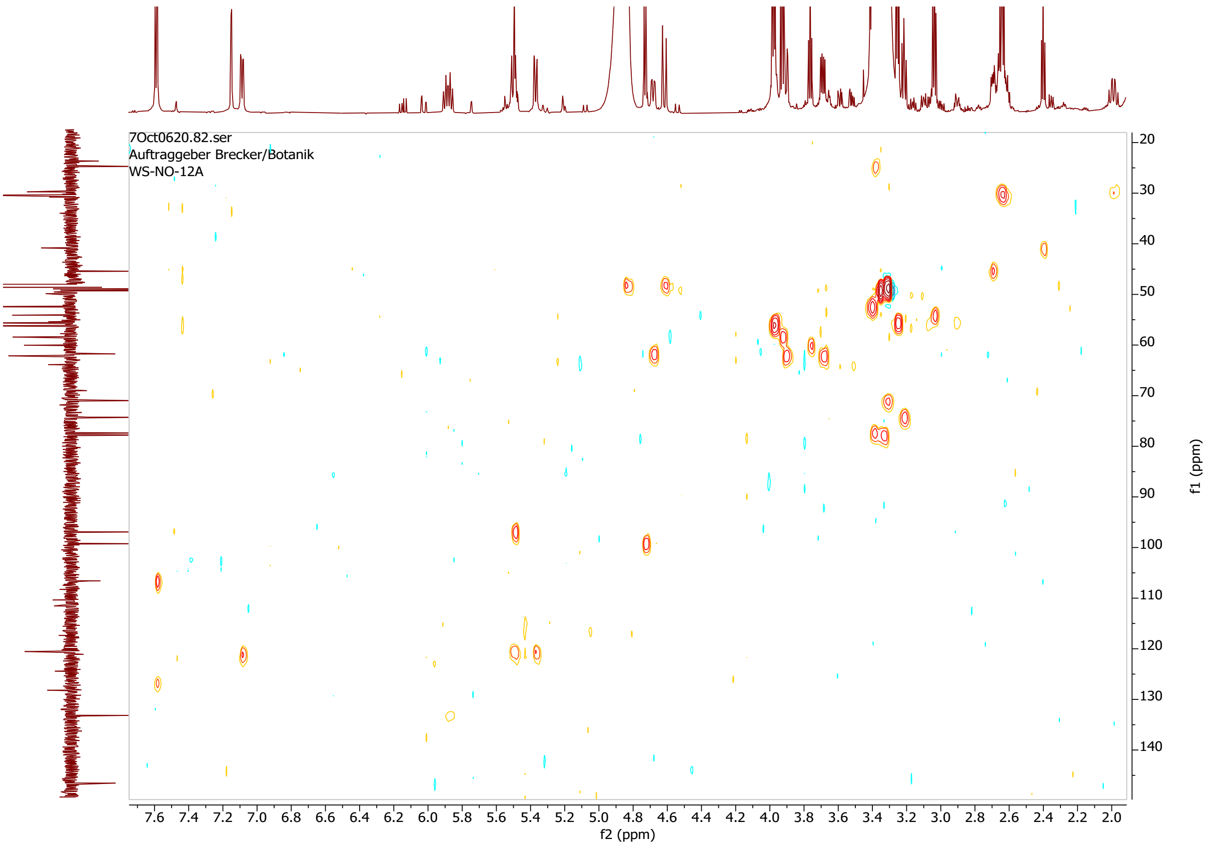

Figure S19. HSQC of 10-hydroxy-3-*epi*-pumiloside (**4**) in CD_3OD.

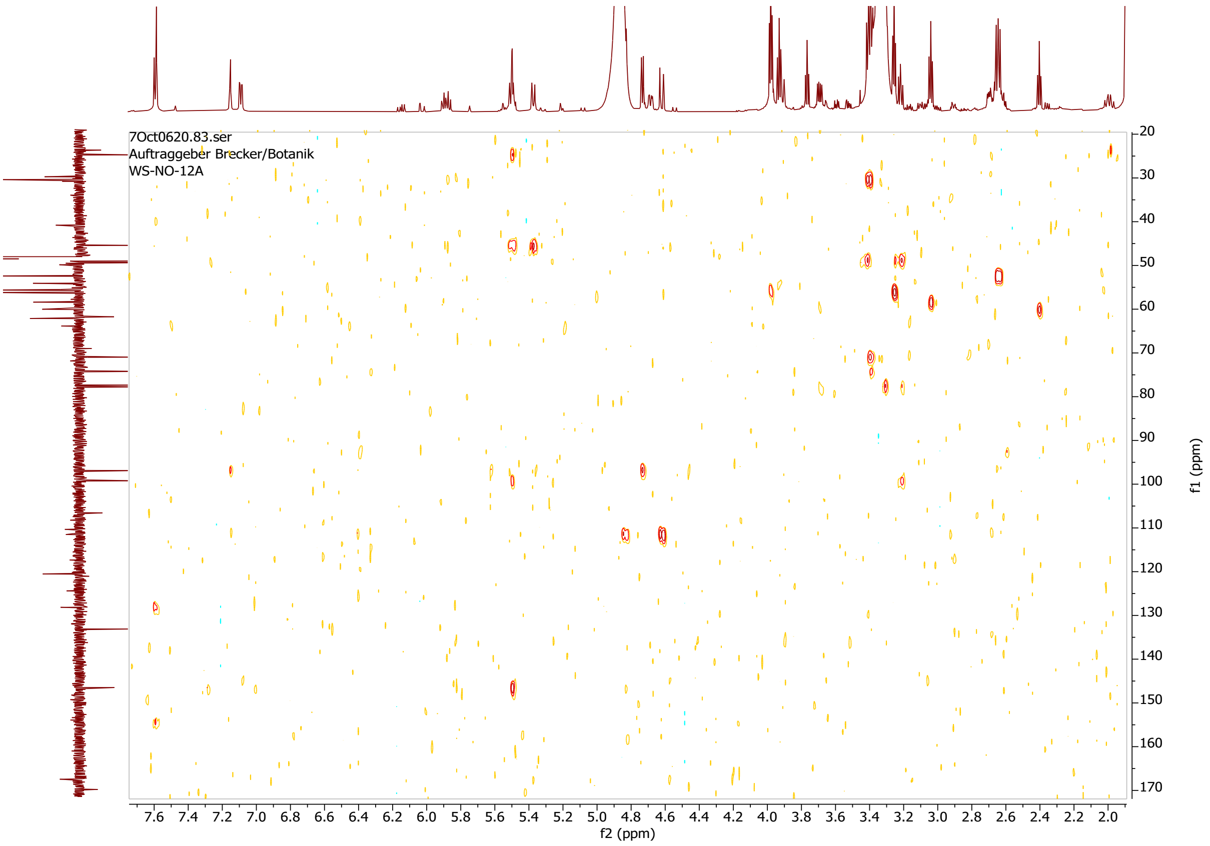

Figure S20. HMBC of 10-hydroxy-3-*epi*-pumiloside (**4**) in CD_3OD.

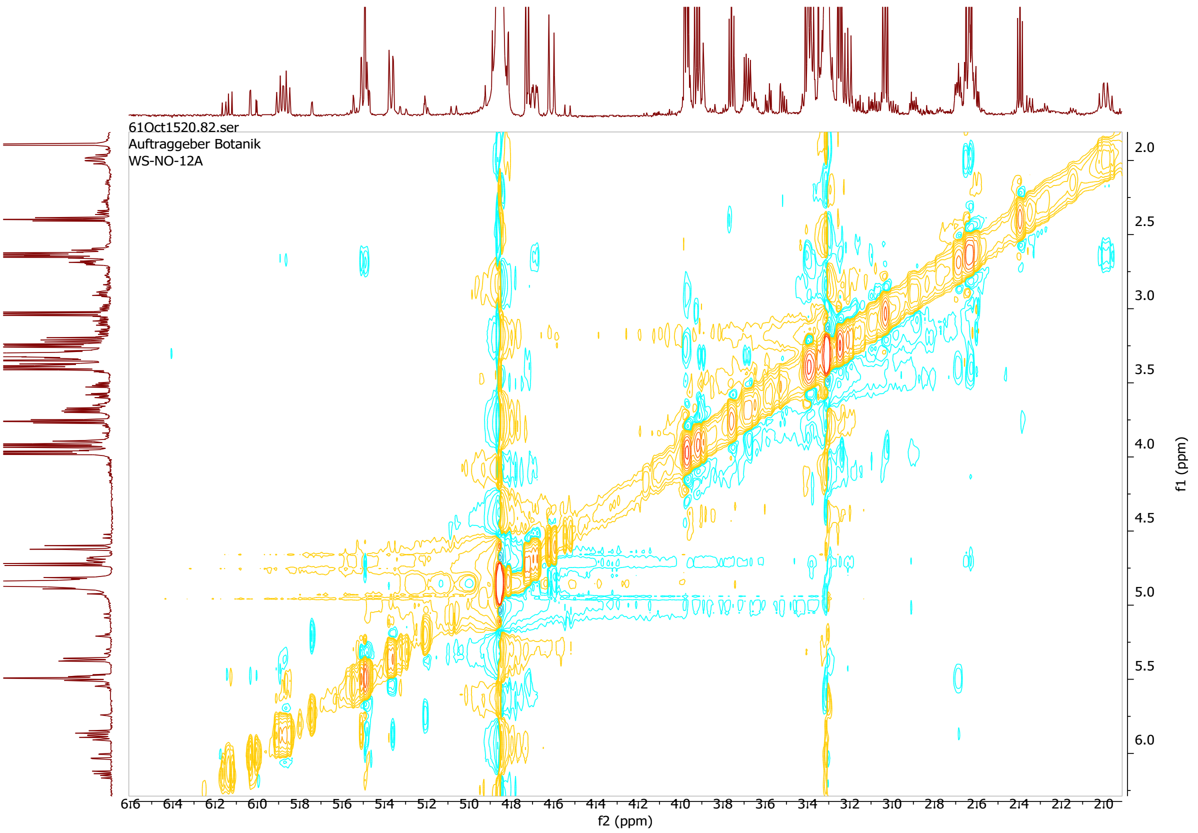

Figure S21. NOESY of 10-hydroxy-3-*epi*-pumiloside (**4**) in CD_3OD.

Generic Display Report

Analysis Info

Analysis Name	E:\Data\MS_MessService\73748000001.d	Acquisition Date	10/8/2020 12:07:49 PM
Method	tune_low_MS_Service_10_20.m	Operator	msc
Sample Name	WS-NO-12A	Instrument	maXis
Comment	Weerasak Songoen/Brecker<br>Ergebnis +/- 5ppm<br>ACN/MeOH + 1% H2O		

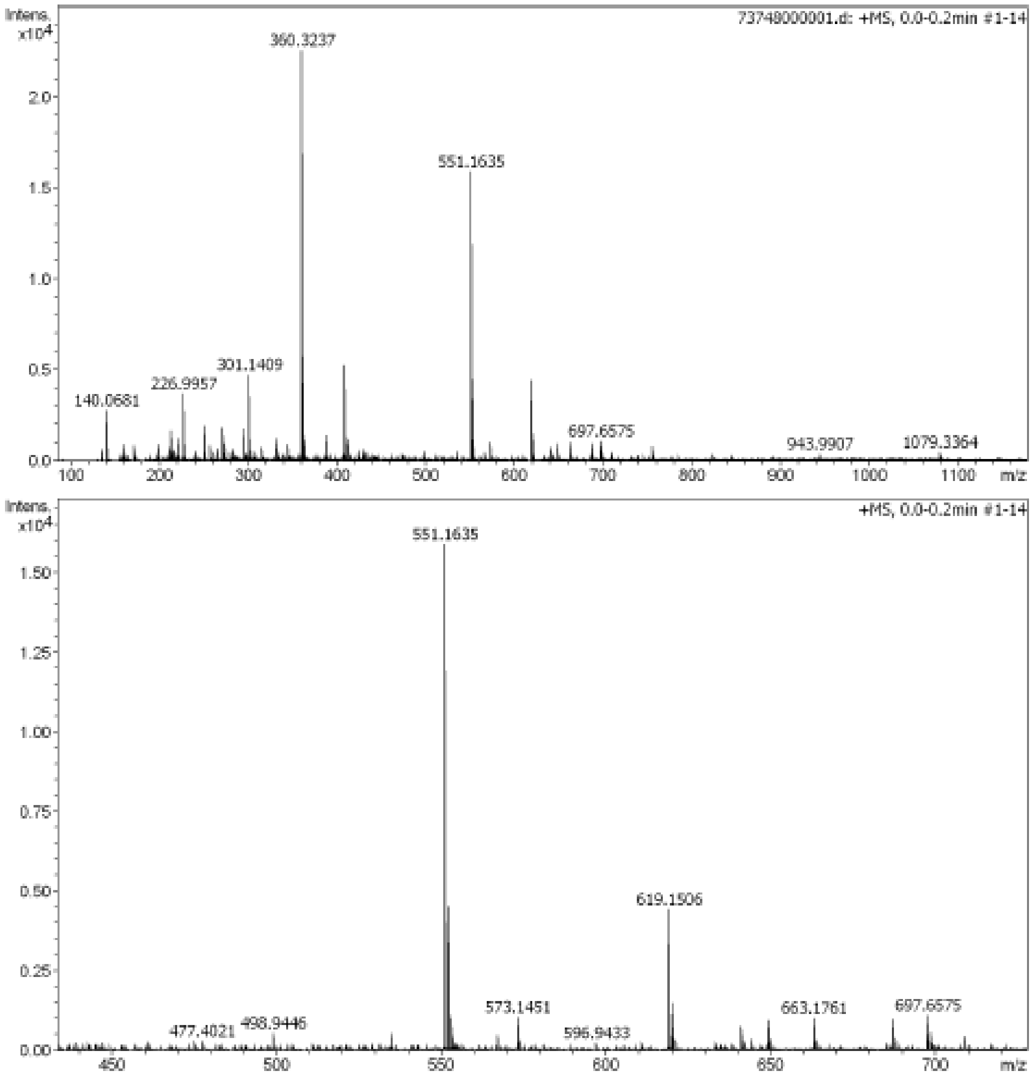

Bruker Compass DataAnalysis 4.1 printed: 10/8/2020 12:12:48 PM by: msc Page 1 of 1

Figure S22. Mass spectrum of 10-hydroxy-3-*epi*-pumiloside (**4**).

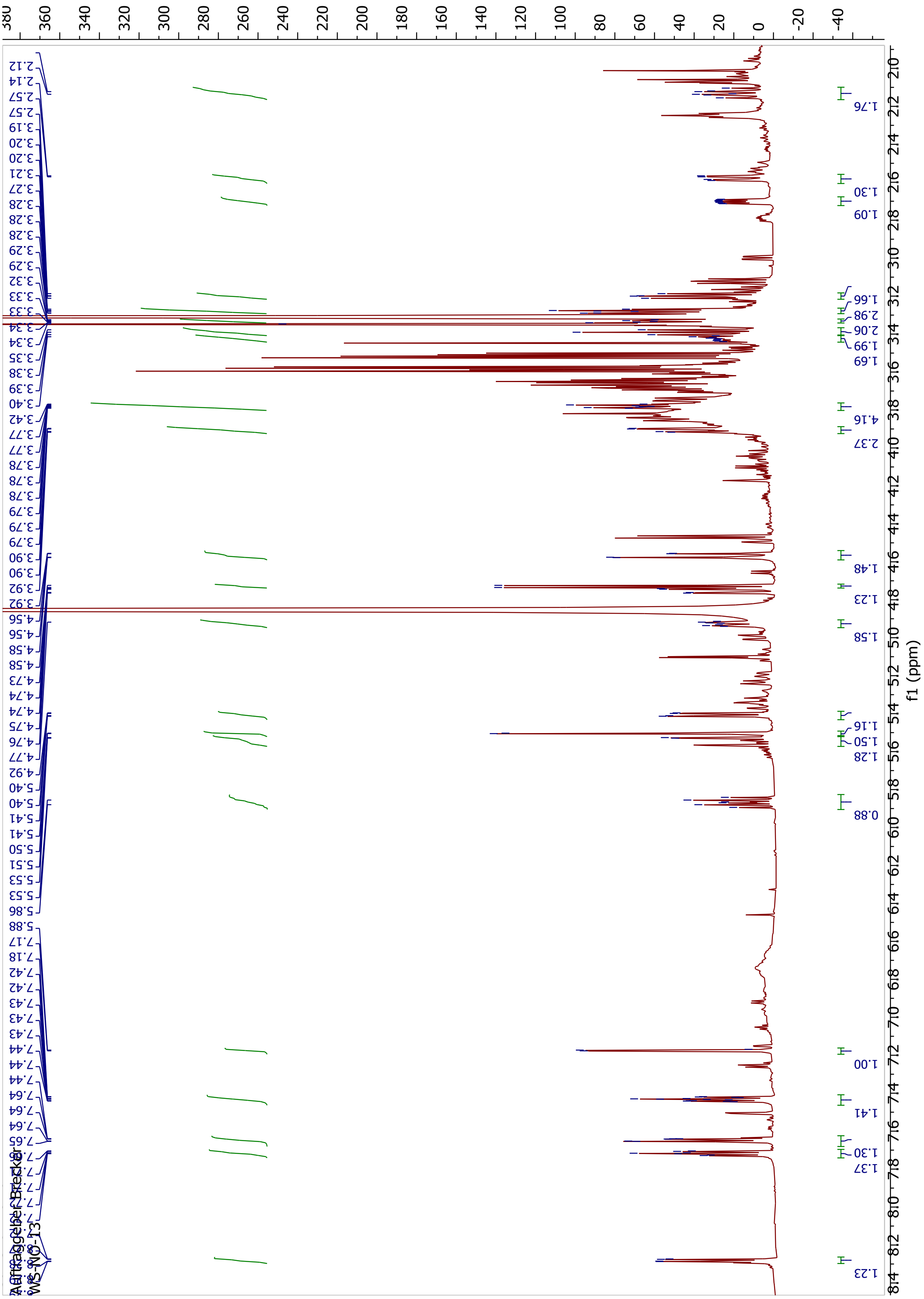

Figure S23. ^{1}H NMR of 3-*epi*-pumiloside (**5**) in CD_3OD.

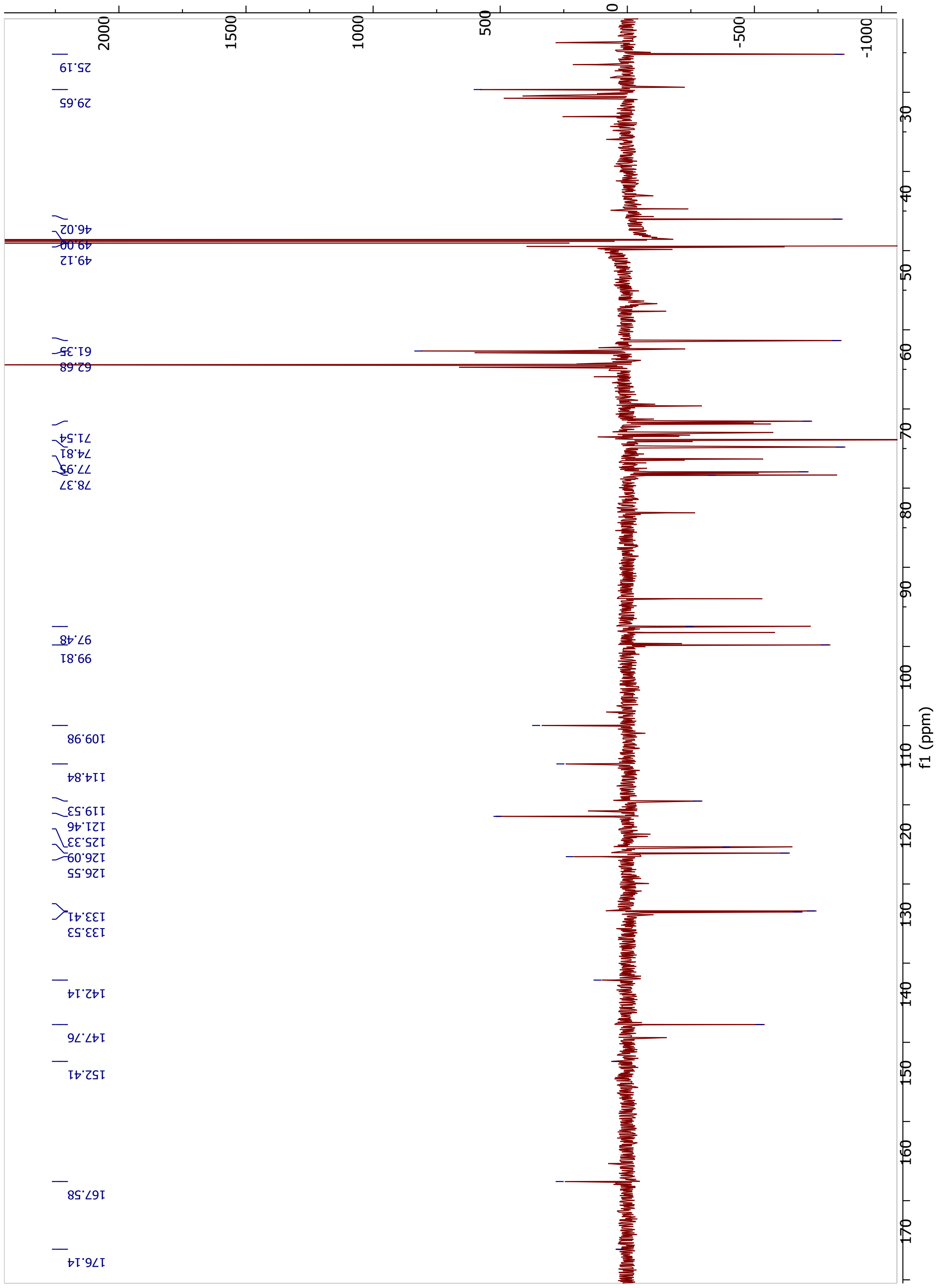

Figure S24. ^{13}C NMR of 3-*epi*-pumiloside (**5**) in CD_3OD.

Mass Spectrum SmartFormula Report

Analysis Info

Analysis Name	E:\Data\MS_MessService\71594000001.d	Acquisition Date	7/13/2020 9:43:01 AM
Method	tune_low_MS_Service_07_20.m	Operator	msc
Sample Name	WS_NO_13	Instrument	maXis 255552.00016
Comment	Sonpoen / OC<br>Ergebnis +/- 5ppm<br>ACN/MeOH + 1 % H2O		

Acquisition Parameter

Source Type	ESI	Ion Polarity	Positive	Set Nebulizer	0.4 Bar
Focus	Not active	Set Capillary	4200 V	Set Dry Heater	180 °C
Scan Begin	50 m/z	Set End Plate Offset	-500 V	Set Dry Gas	4.0 l/min
Scan End	1900 m/z	Set Charging Voltage	0 V	Set Divert Valve	Source
		Set Corona	0 nA	Set APCI Heater	0 °C

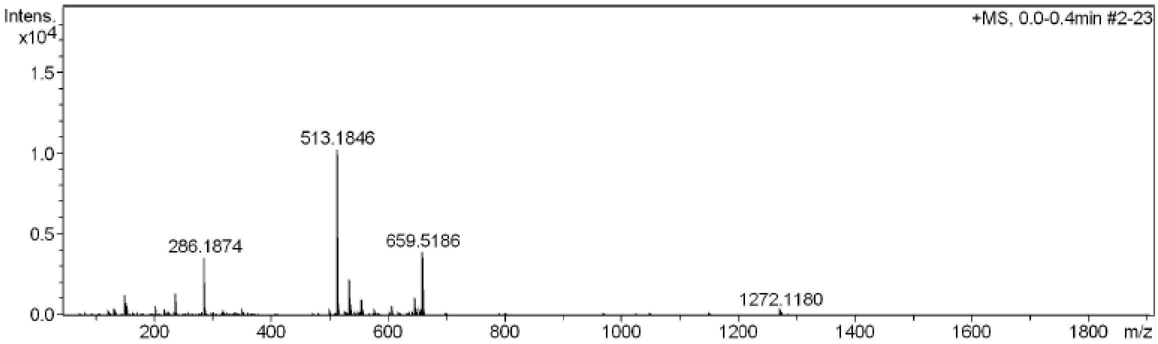

Figure S25. Mass spectrum of 3-*epi*-pumiloside (**5**).

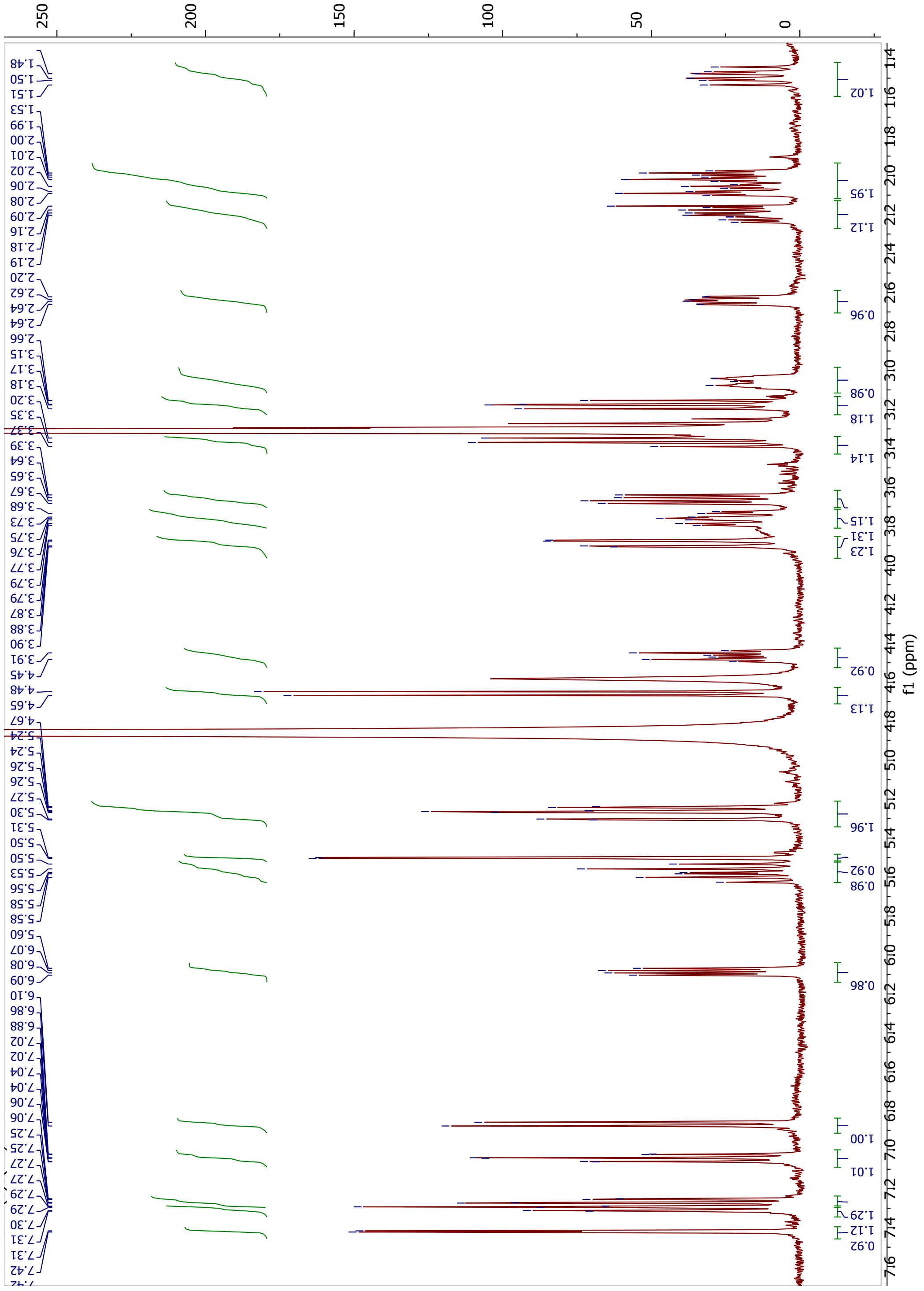

Figure S26. ^{1}H NMR of nauclealomide B (**6**) in CD_3OD.

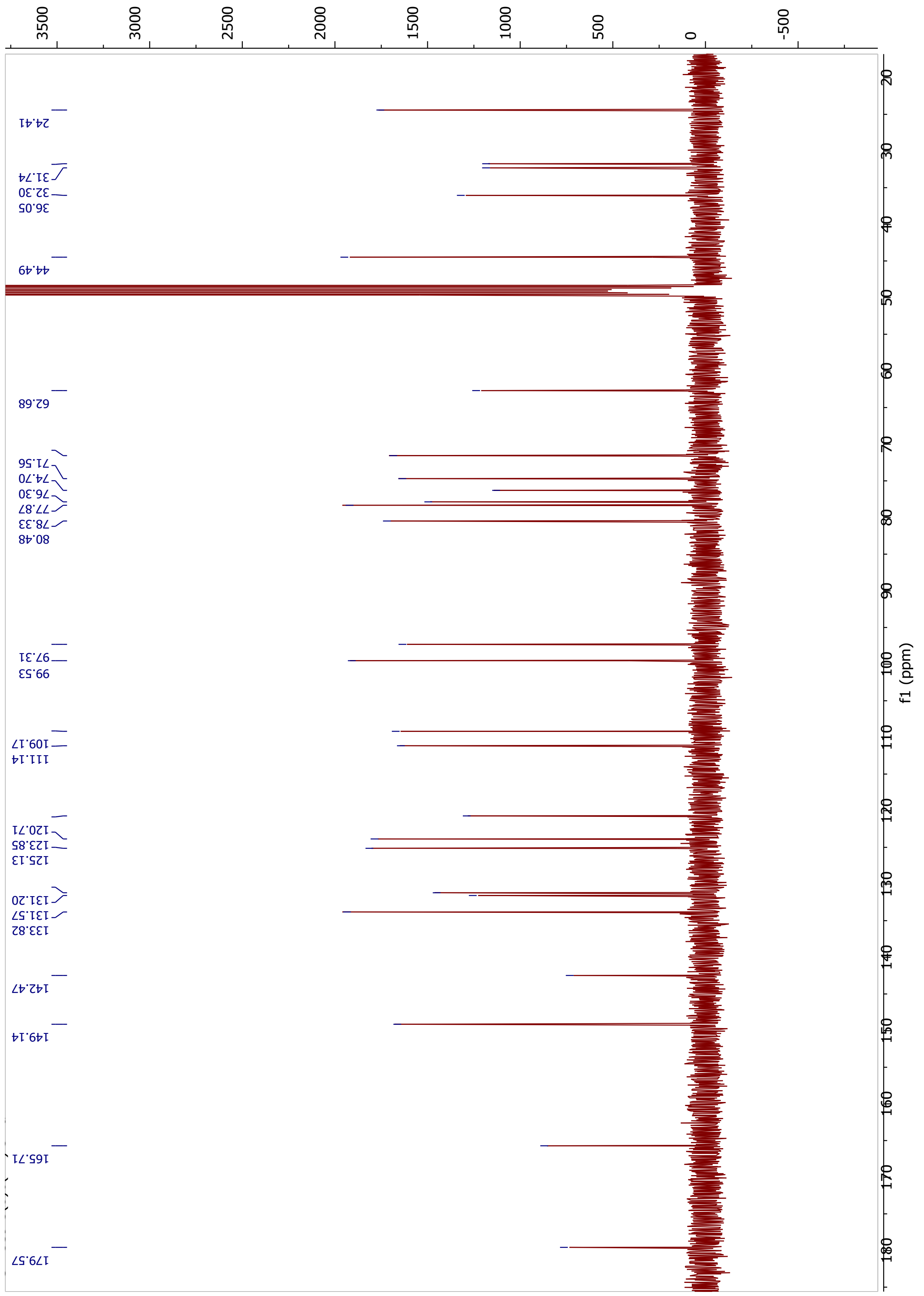

Figure S27. ^{13}C NMR of nauclealomide B (**6**) in CD_3OD.

Generic Display Report

Analysis Info

Analysis Name	E:\Data\MS_MessService\71172000001.d	Acquisition Date	6/26/2020 2:10:44 PM
Method	tune_low_MS_Service_06_20.m	Operator	msc
Sample Name	WS-NO-11	Instrument	maXis
Comment	Schedl/Brecker/Botanik<br>Ergebnis +/- 5 ppm<br>ACN/MeOH + 1% H2O		

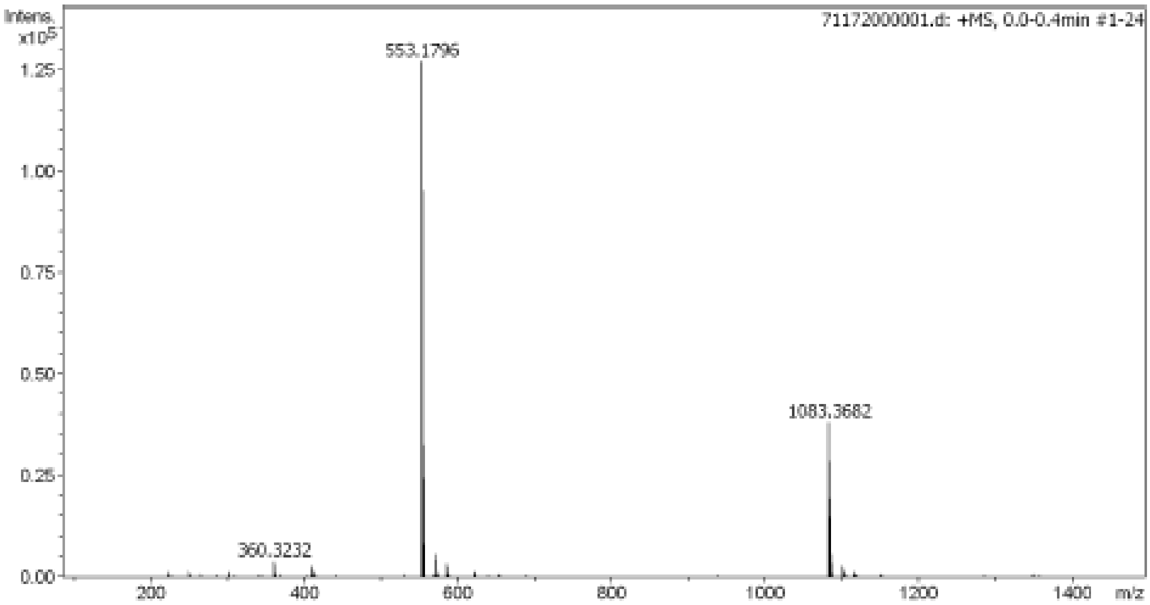

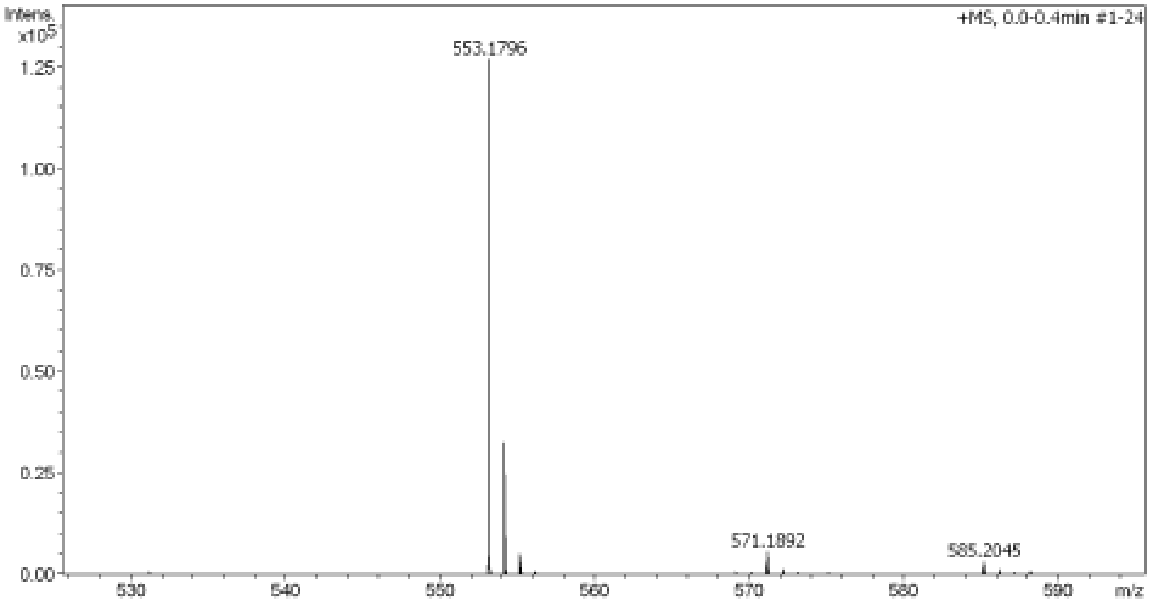

Bruker Compass DataAnalysis 4.1 printed: 6/26/2020 3:57:05 PM by: msc Page 1 of 1

Figure S28. Mass spectrum of nauclealomide B (**6**).

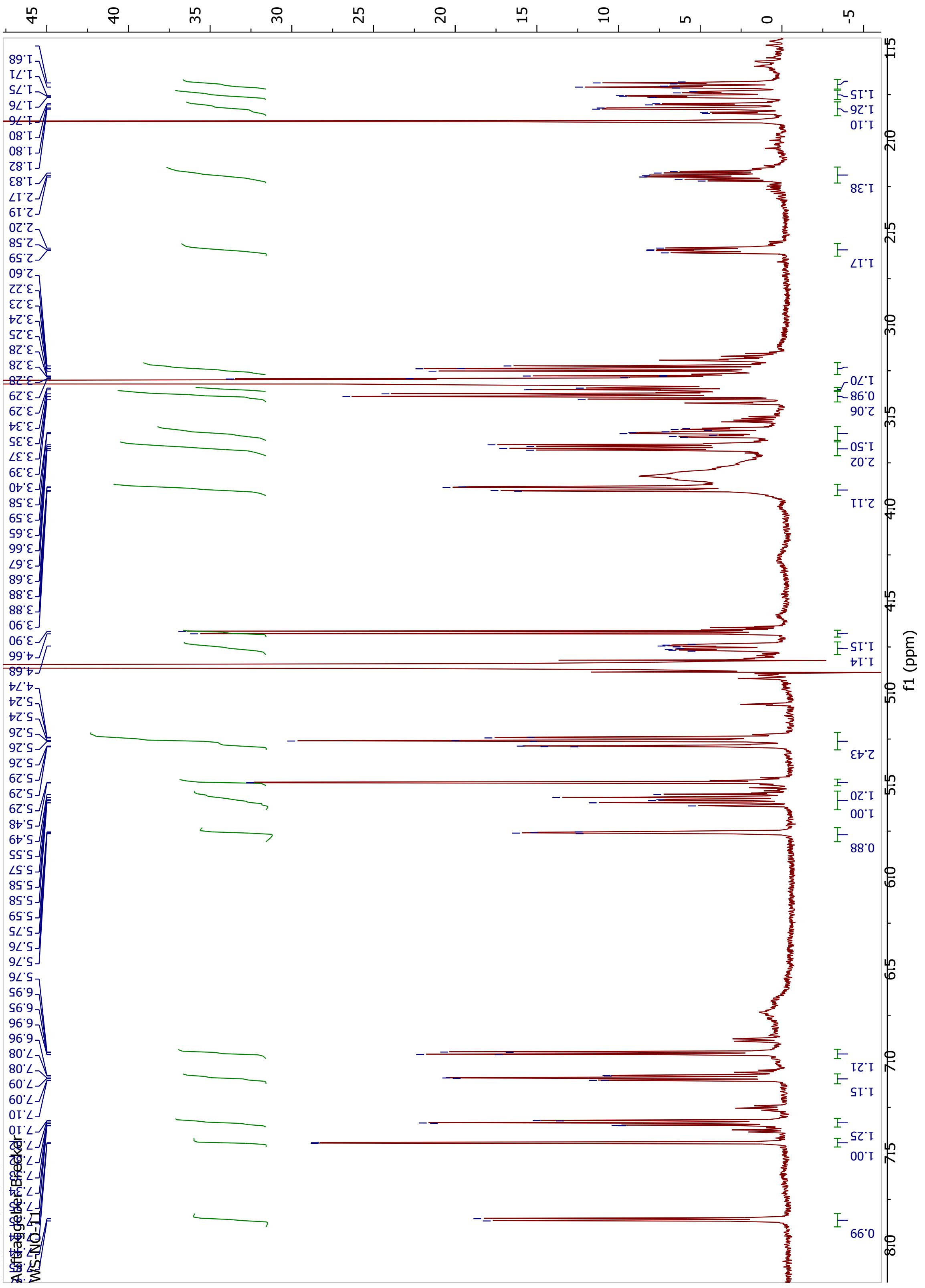

Figure S29. ^{1}H NMR of nauclealomide C (**7**) in CD_3OD.

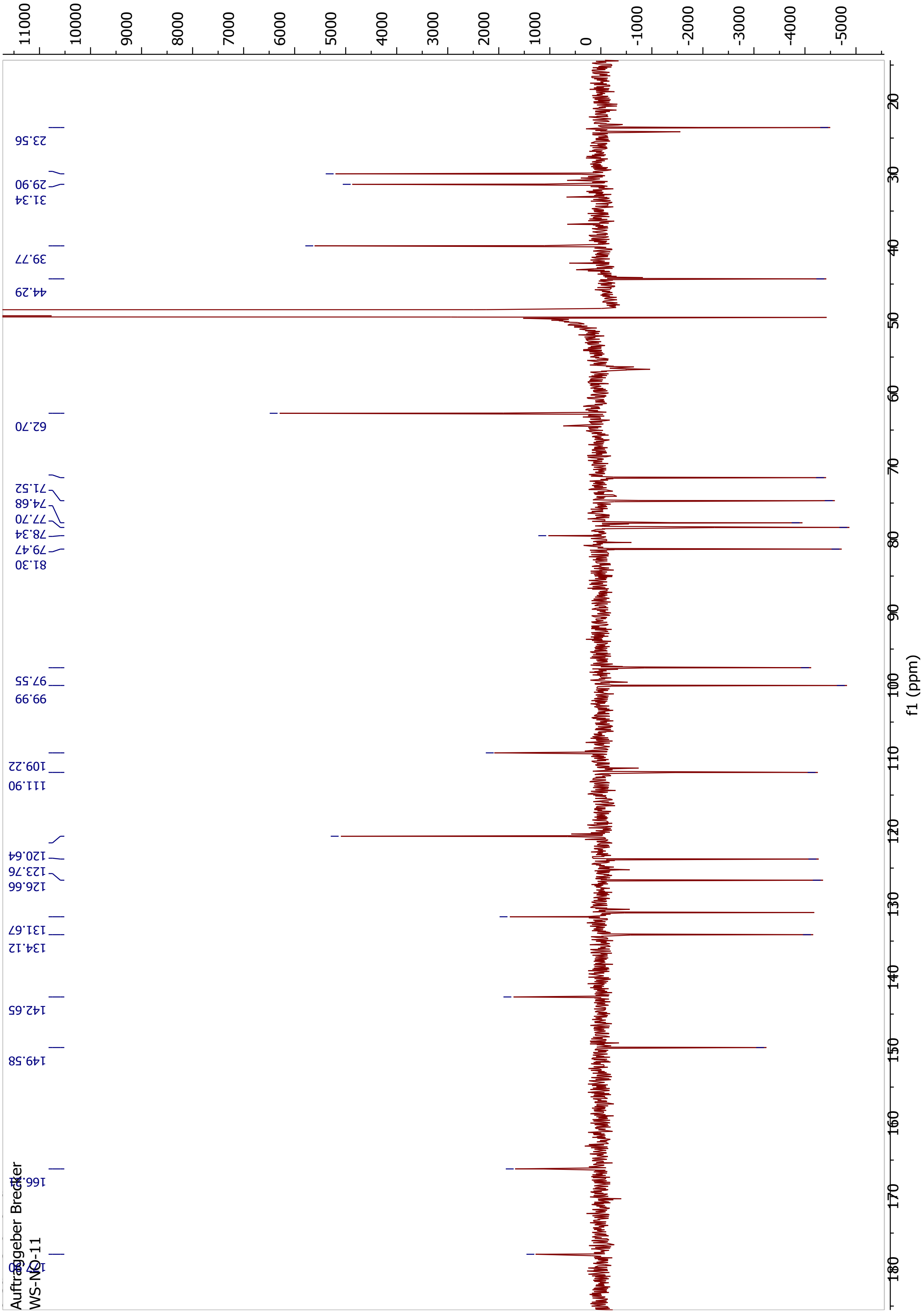

Figure S30. ^{13}C NMR of nauclealomide C (**7**) in CD_3OD.

Generic Display Report

Analysis Info

Analysis Name	E:\Data\MS_MessService\74154000001.d	Acquisition Date	10/21/2020 7:20:56 AM
Method	tune_low_MS_Service_10_20.m	Operator	msc
Sample Name	Naucleomide c	Instrument	maXis
Comment	Weerasak / Brecker		
	Ergebnis +/- 5ppm		
	ACN/MeOH + 1% H2O		

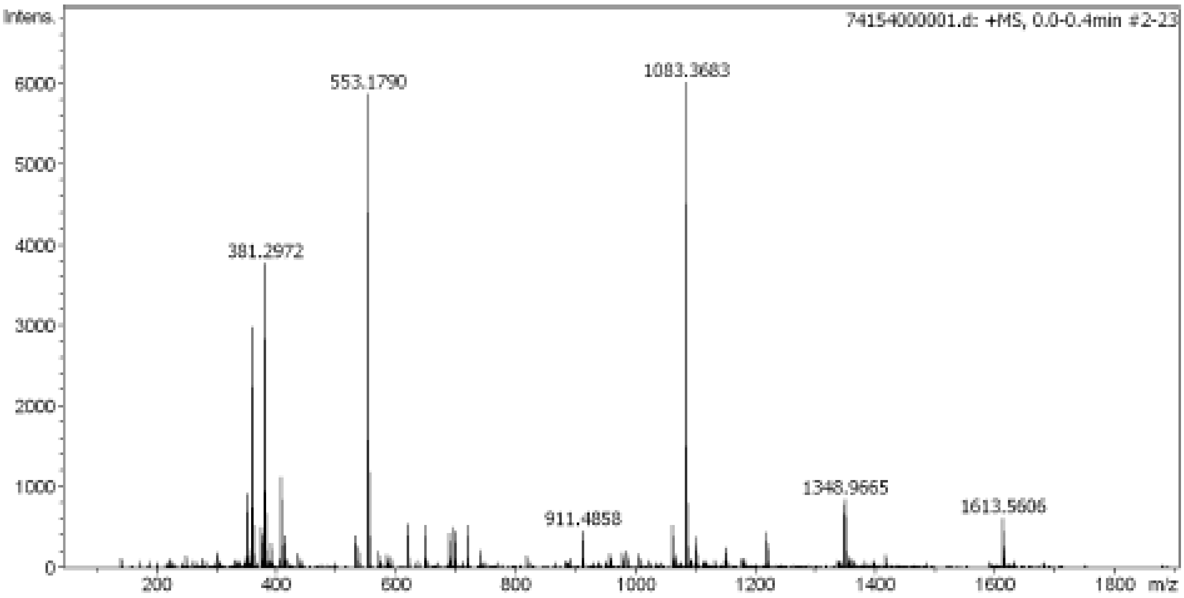

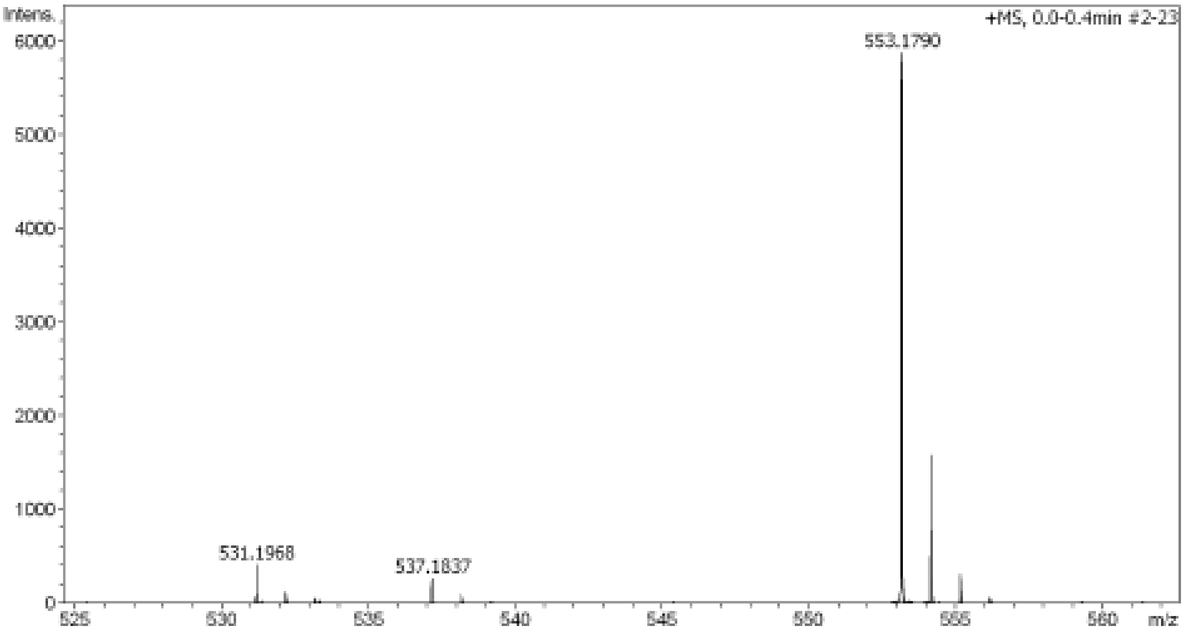

Bruker Compass DataAnalysis 4.1 printed: 10/21/2020 7:23:00 AM by: msc Page 1 of 1

Figure S31. Mass spectrum of nauclealomide C (**7**).

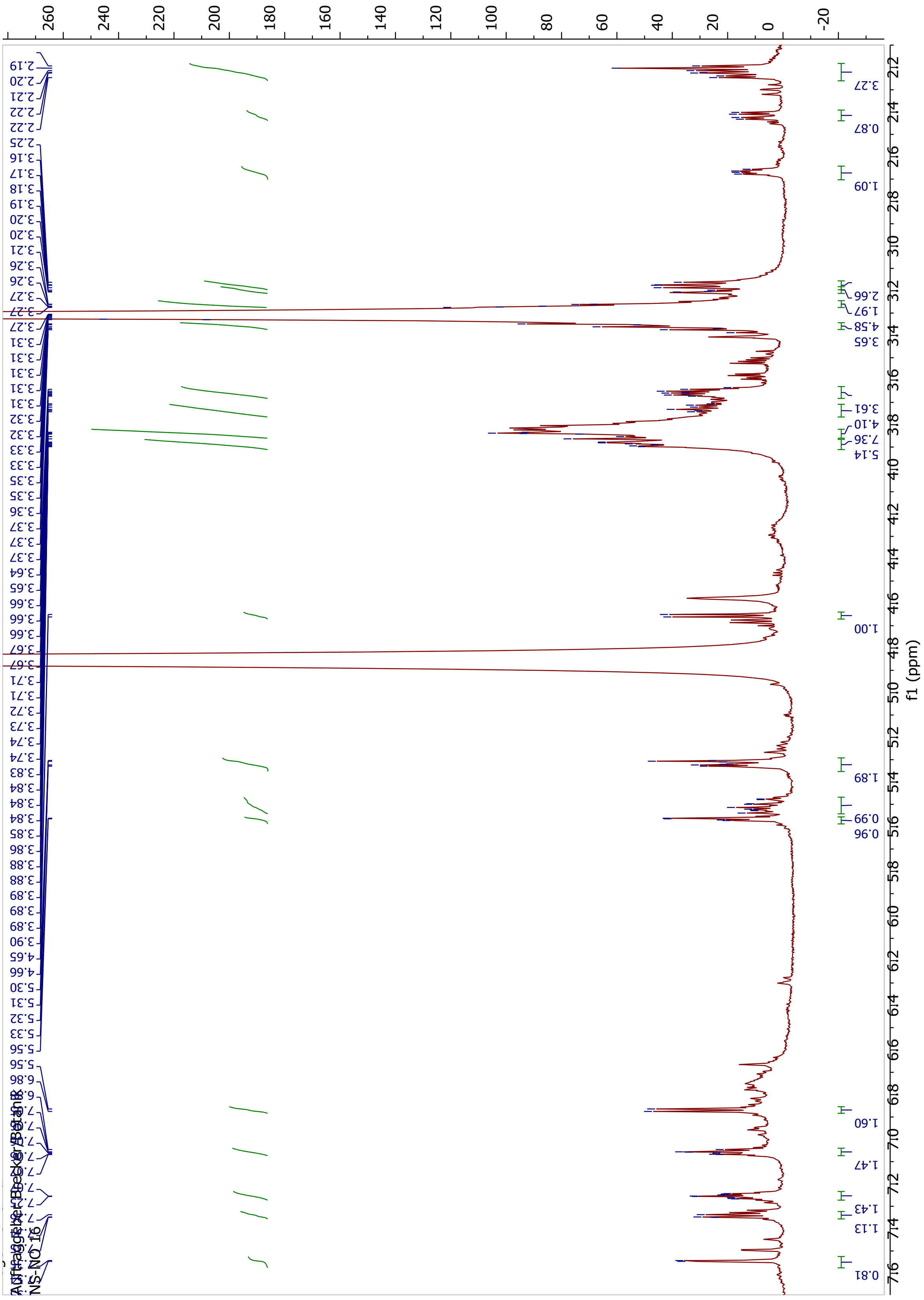

Figure S32. ^{1}H NMR of paratunamide C (**8**) in CD_3OD.

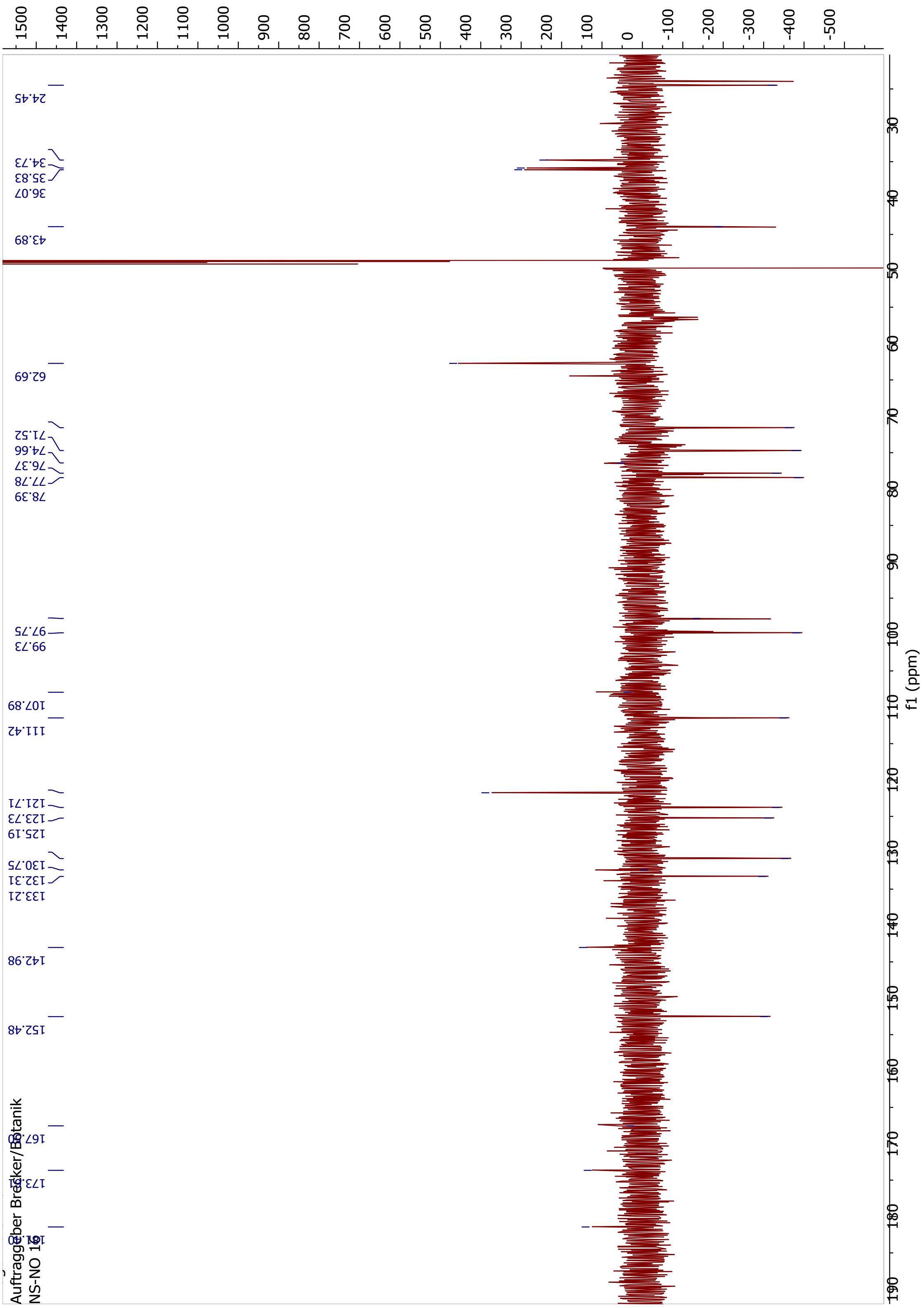

Figure S33. ^{13}C NMR of paratunamide C (**8**) in CD_3OD.

Generic Display Report

Analysis Info

Analysis Name	E:\Data\MS_MessService\72265000001.d	Acquisition Date	8/14/2020 9:39:42 AM
Method	tune_low_MS_Service_08_20.m	Operator	msc
Sample Name	NS-NO-16	Instrument	maXis
Comment	Traxler/Brecker		
	Ergebnis +/- 5ppm		
	ACN / MeOH + 1% H2O		

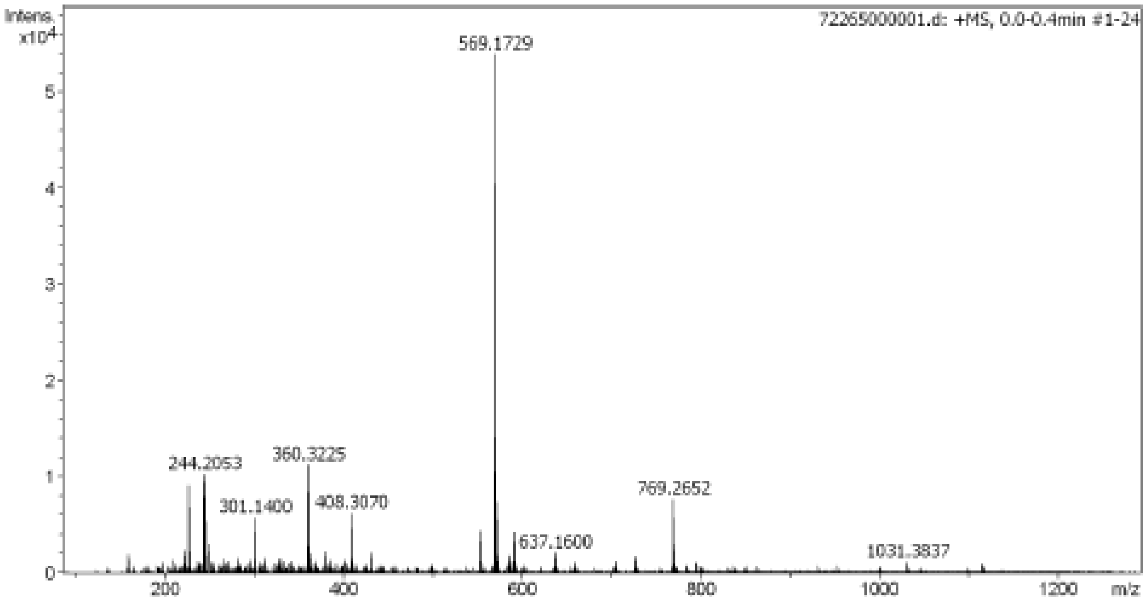

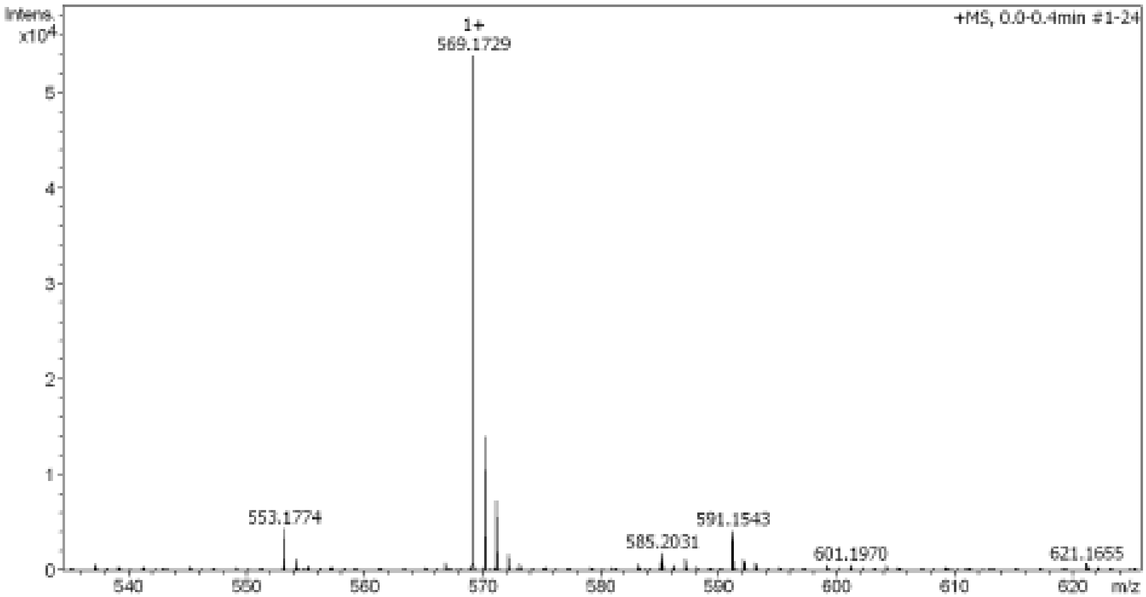

Bruker Compass DataAnalysis 4.1 printed: 8/14/2020 2:18:20 PM by: msc Page 1 of 1

Figure S34. Mass spectrum of paratunamide C (**8**).

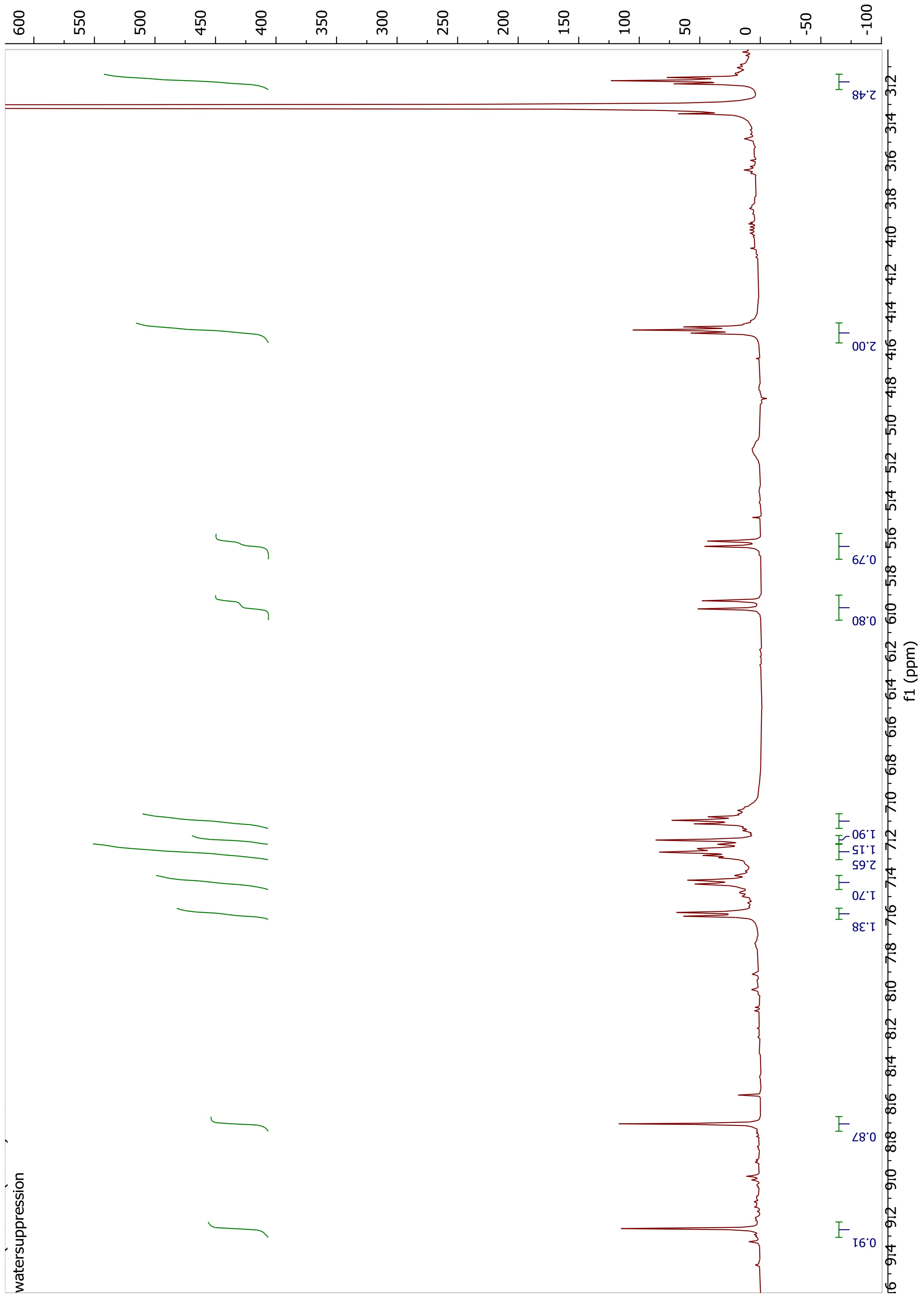

Figure S35. ^{1}H NMR of angustine (**9**) in CD_3OD.

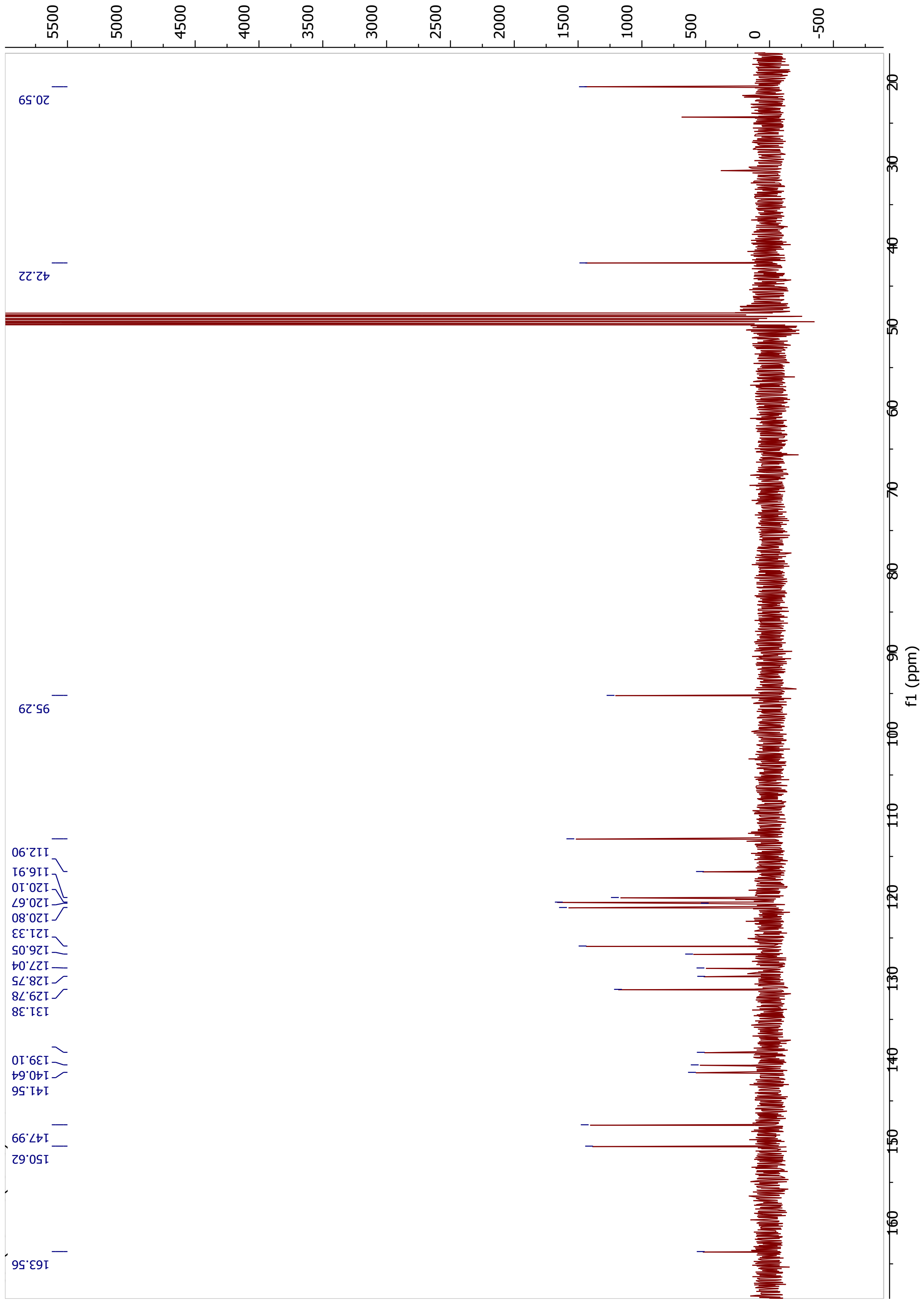

Figure S36. ^{13}C NMR of angustine (**9**) in CD_3OD.

Mass Spectrum SmartFormula Report

Analysis Info

Analysis Name	D:\Data\20181005\WANCHAI000001.d	Acquisition Date	05-Oct-18 1:09:34 PM
Method	APCI_pos_direct probe 50-1000.m	Operator	BDAL@DE
Sample Name	WS91	Instrument	micrOTOF-Q III 8228888.20478
Comment			

Acquisition Parameter

Source Type	APCI	Ion Polarity	Positive	Set Nebulizer	1.6 Bar
Focus	Not active	Set Capillary	4000 V	Set Dry Heater	200 °C
Scan Begin	50 m/z	Set End Plate Offset	-500 V	Set Dry Gas	8.0 l/min
Scan End	600 m/z	Set Collision Cell RF	100.0 Vpp	Set Divert Valve	Source

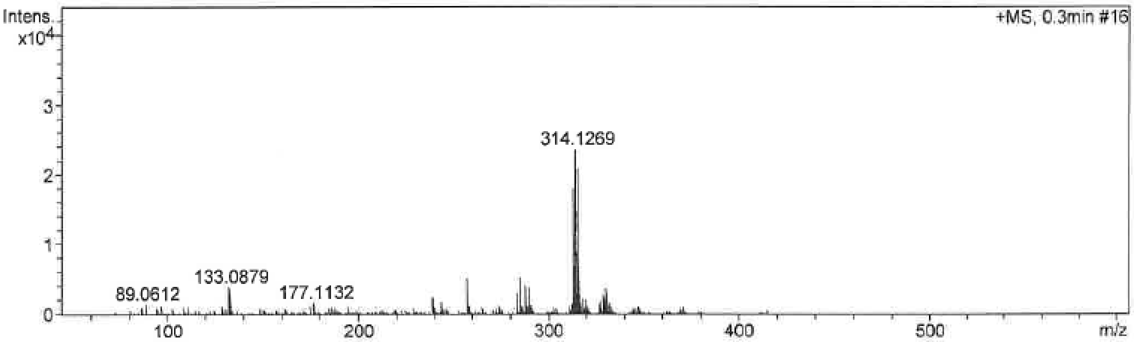

Meas. m/z	#	Ion Formula	m/z	err [mDa]	mSigma	# Sigma	Score	rdb	e^- Conf	N-Rule
314.1269	1	C20H16N3O	314.1288	1.9	413.0	1	100.00	14.5	even	ok

Figure S37. Mass spectrum of angustine (**9**).

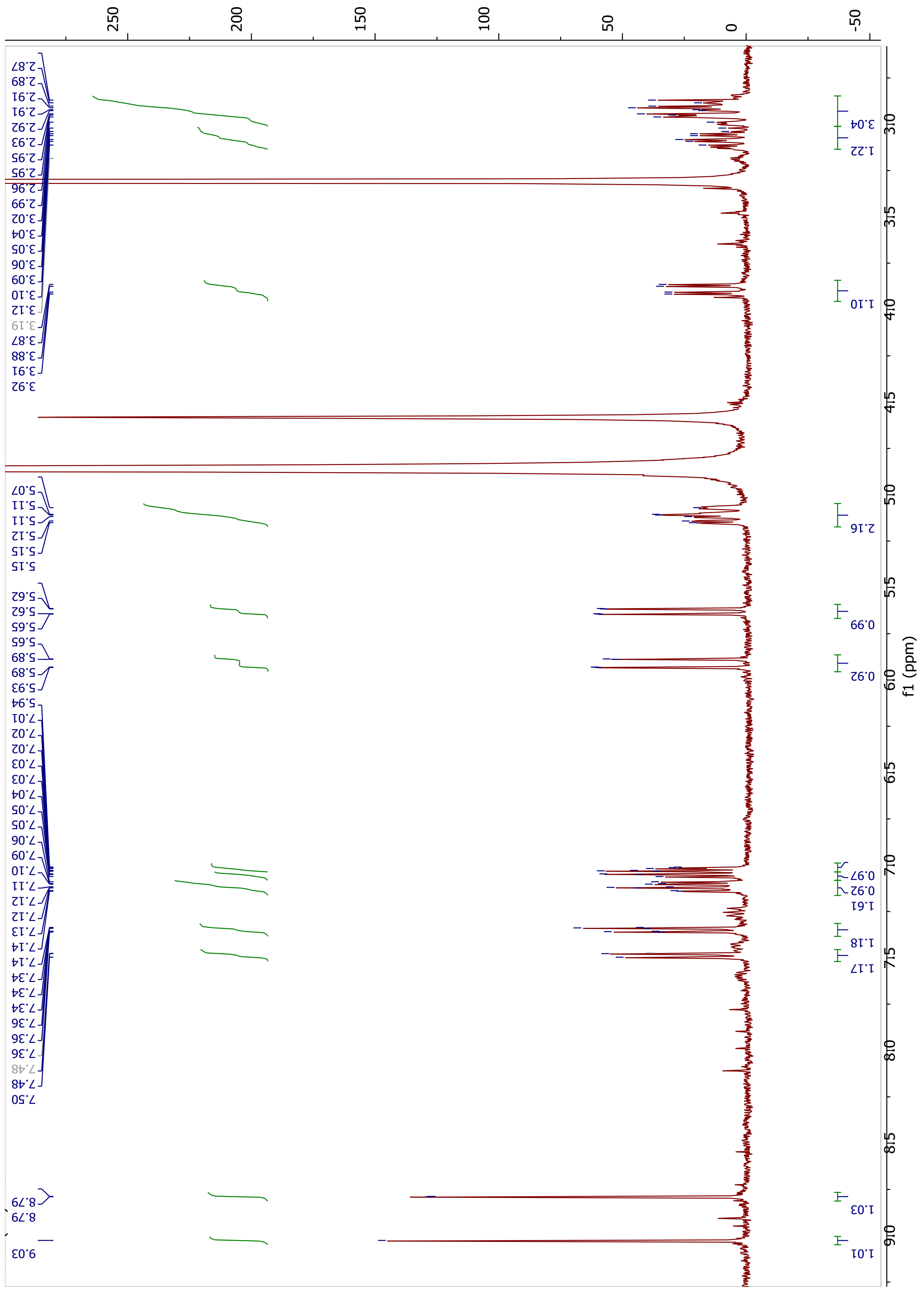

Figure S38. ^{1}H NMR of 3,14-dihydroangustine (**10**) in CD_3OD.

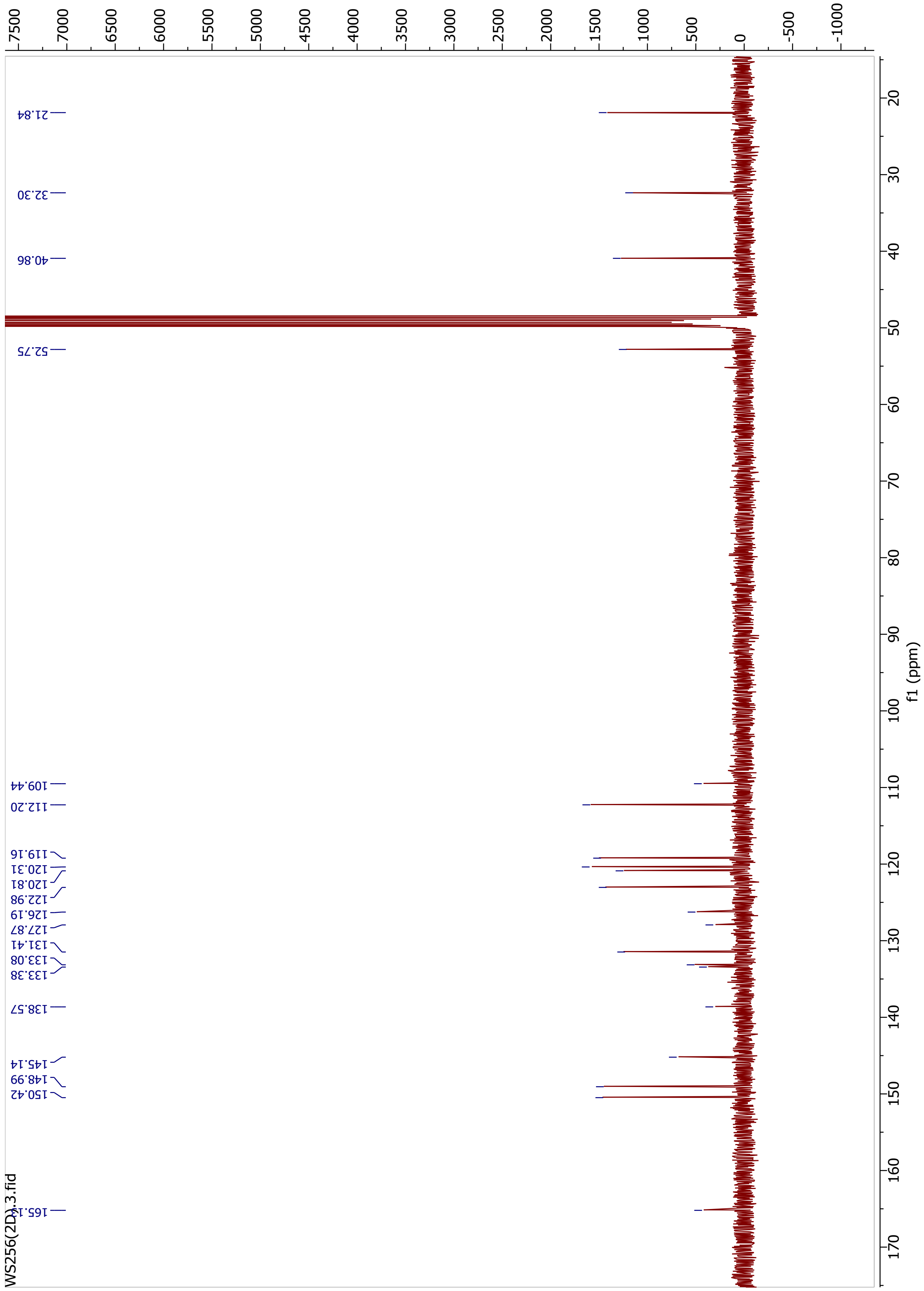

Figure S39. ^{13}C NMR of 3,14-dihydroangustine (**10**) in CD_3OD.

Display Report

Analysis Info

Analysis Name	D:\Data\Wanchai210217\Wanchai000003.d	Acquisition Date	17-Feb-21 10:19:41 AM
Method	tune_low_pos.m	Operator	BDAL@DE
Sample Name	Ws256	Instrument	micrOTOF-Q III 8228888.20478
Comment			

Acquisition Parameter

Source Type	ESI	Ion Polarity	Positive	Set Nebulizer	0.3 Bar
Focus	Not active	Set Capillary	4500 V	Set Dry Heater	180 °C
Scan Begin	50 m/z	Set End Plate Offset	-500 V	Set Dry Gas	4.0 l/min
Scan End	600 m/z	Set Collision Cell RF	100.0 Vpp	Set Divert Valve	Source

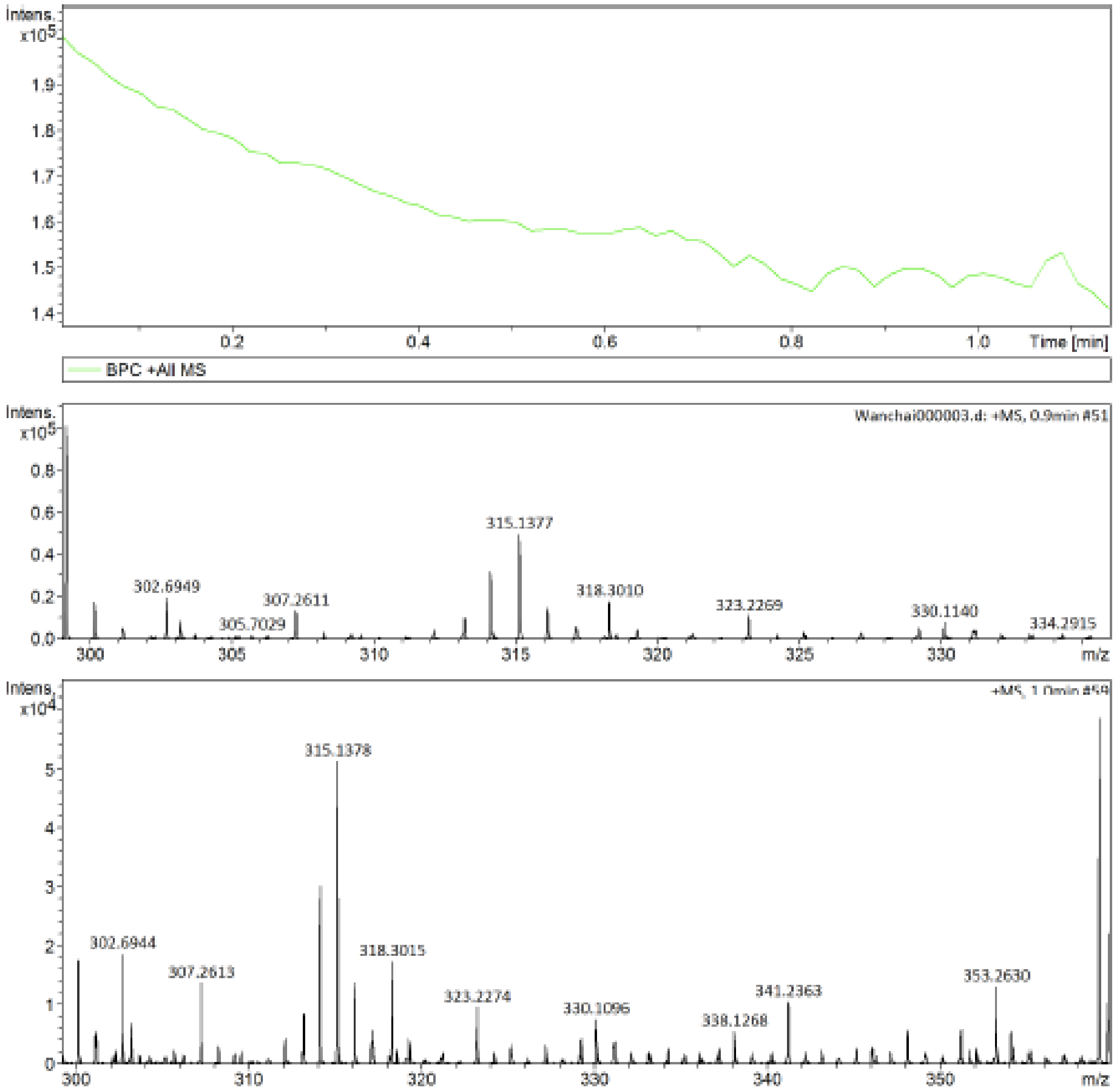

Figure S40. Mass spectrum of 3,14-dihydroangustine (**10**).

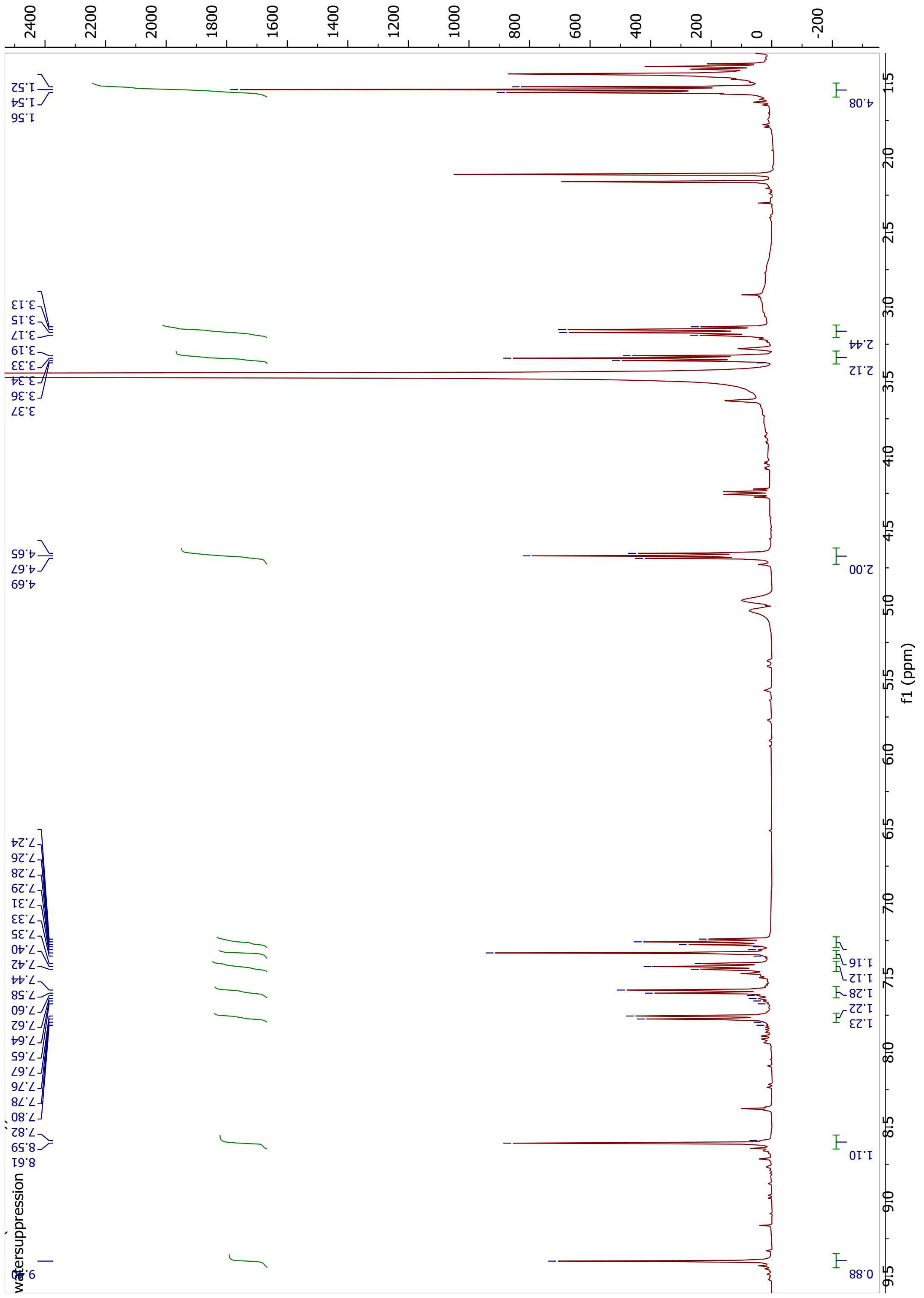

Figure S41. ^{1}H NMR of 18,19-dihydroangustine (**11**) in CD_3OD.

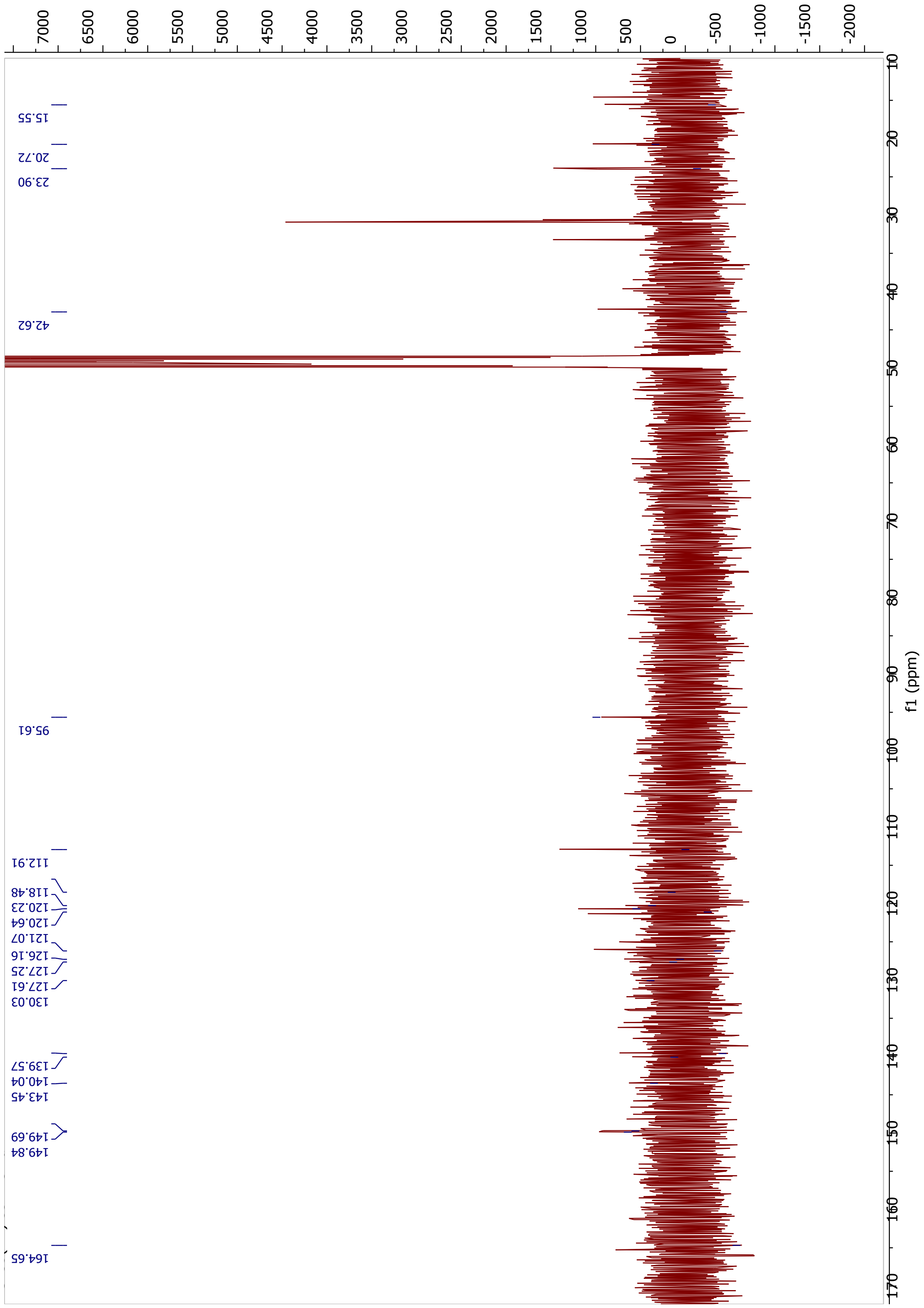

Figure S42. ^{13}C NMR of 18,19-dihydroangustine (**11**) in CD_3OD.

Mass Spectrum List Report

Analysis Info

Analysis Name	D:\Data\20180919\Wanchai000002.d	Acquisition Date	19-Sep-18 10:40:12 AM
Method	APCI_pos_direct probe 50-1000.m	Operator	BDAL@DE
Sample Name	1	Instrument	micrOTOF-Q III 8228888.20478
Comment	MeOH		

Acquisition Parameter

Source Type	APCI	Ion Polarity	Positive	Set Nebulizer	1.6 Bar
Focus	Not active	Set Capillary	4000 V	Set Dry Heater	200 °C
Scan Begin	50 m/z	Set End Plate Offset	-500 V	Set Dry Gas	8.0 l/min
Scan End	500 m/z	Set Collision Cell RF	100.0 Vpp	Set Divert Valve	Source

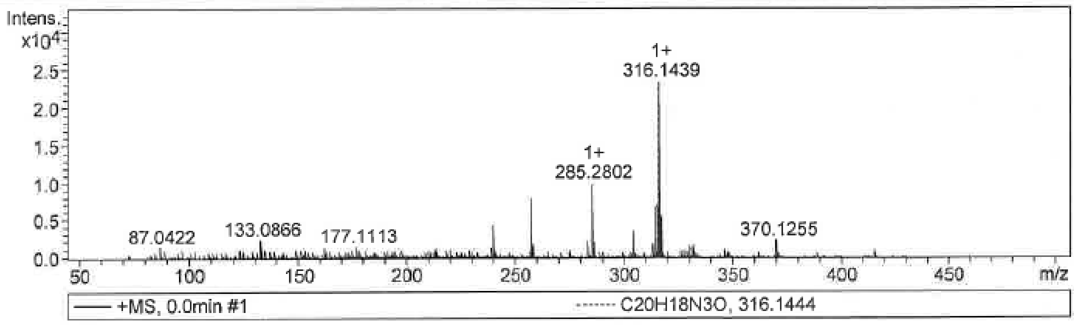

Figure S43. Mass spectrum of 18,19-dihydroangustine (**11**).

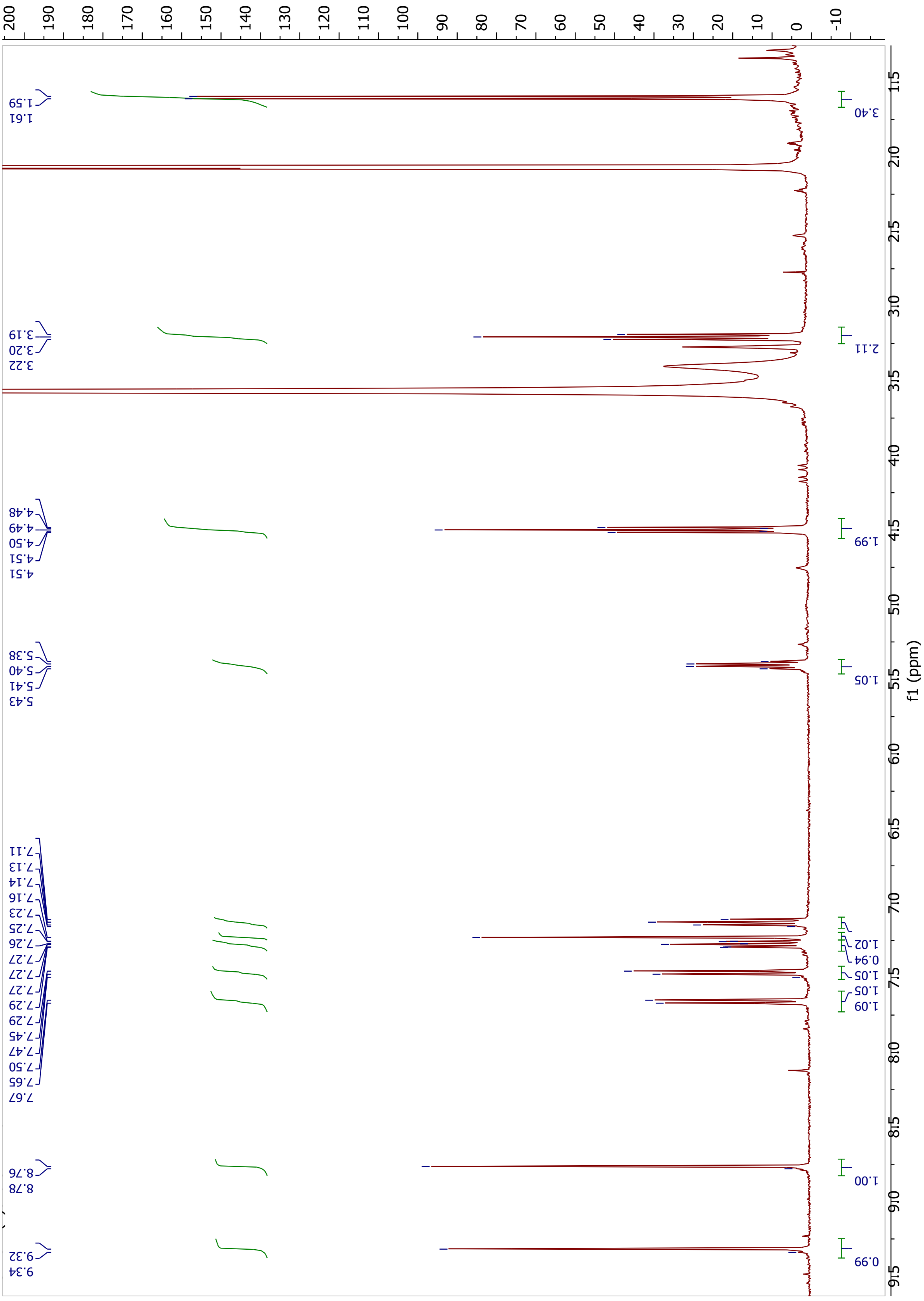

Figure S44. ^{1}H NMR of angustoline (**12**) in CD_3OD.

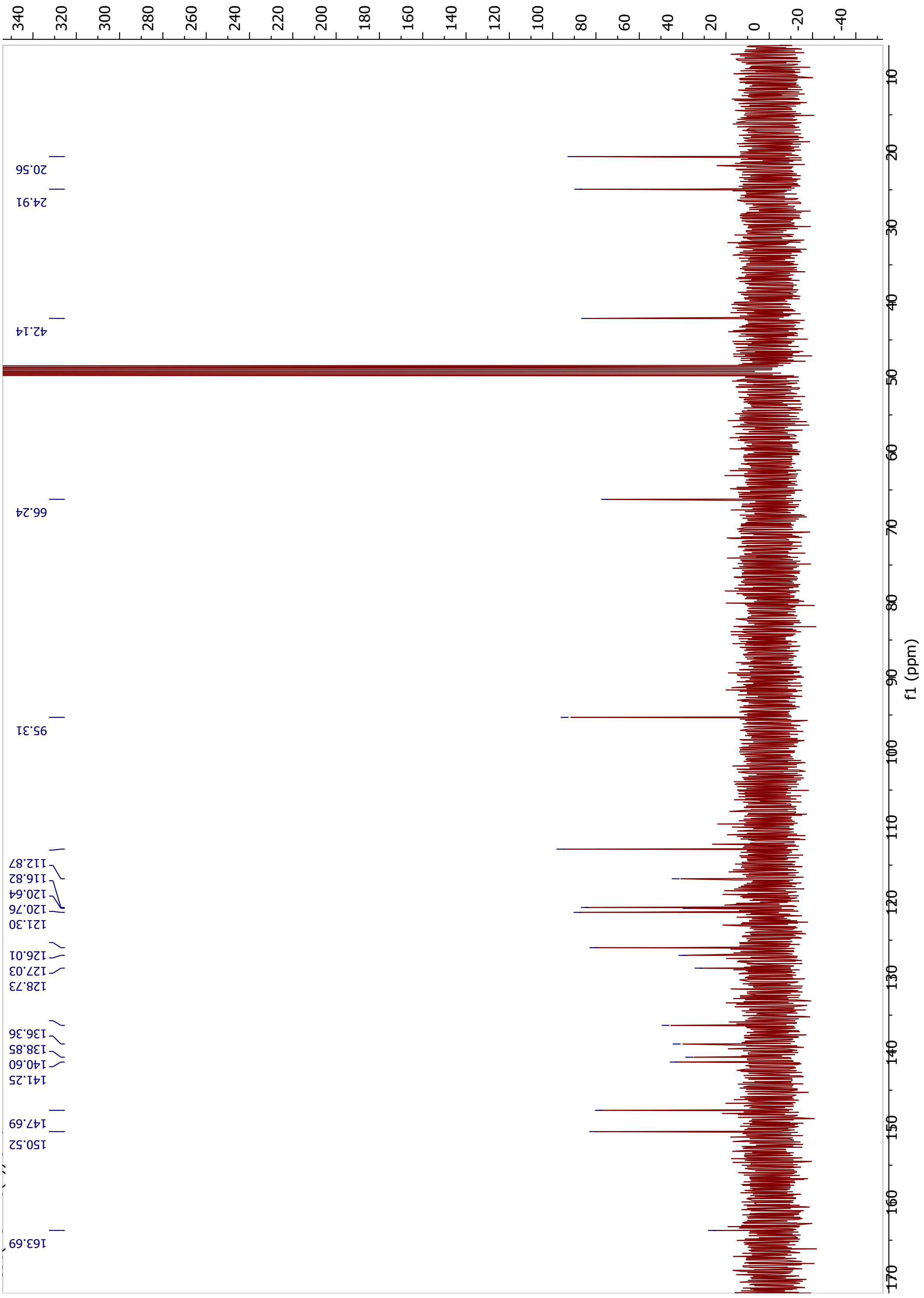

Figure S45. ^{13}C NMR of angustoline (**12**) in CD_3OD.

Generic Display Report

Analysis Info

Analysis Name	E:\Data\MS_MessService\72382000001.d	Acquisition Date	8/17/2020 12:55:14 PM
Method	tune_low_MS_Service_08_20.m	Operator	msc
Sample Name	Angustoline	Instrument	maXis
Comment	Weerasak Songoen/Brecker<br>Ergebnis +/- 5ppm<br>ACN / MeOH + 1% H2O		

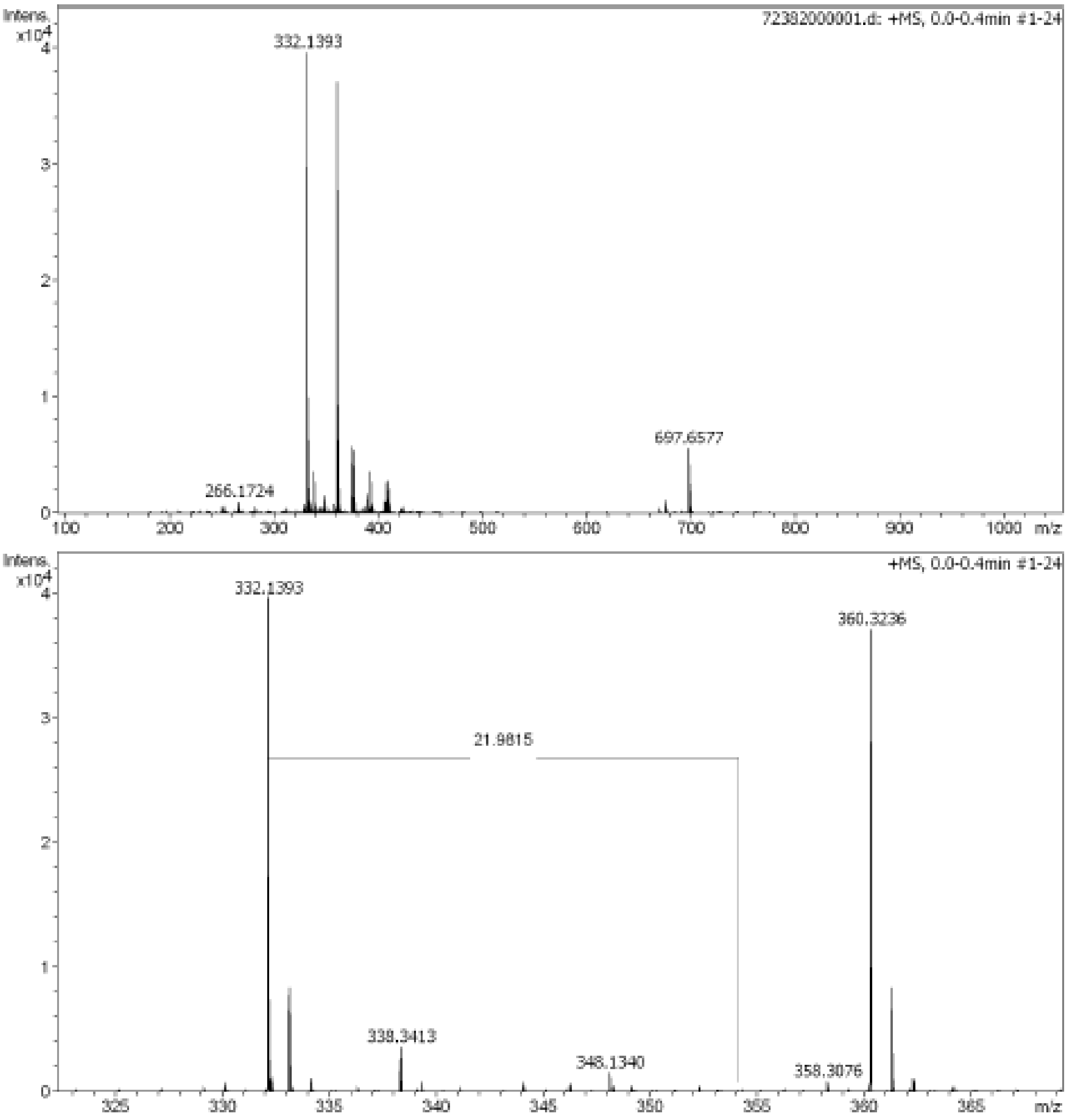

Bruker Compass DataAnalysis 4.1 printed: 8/17/2020 2:19:24 PM by: msc Page 1 of 1

Figure S46. Mass spectrum of angustoline (**12**).

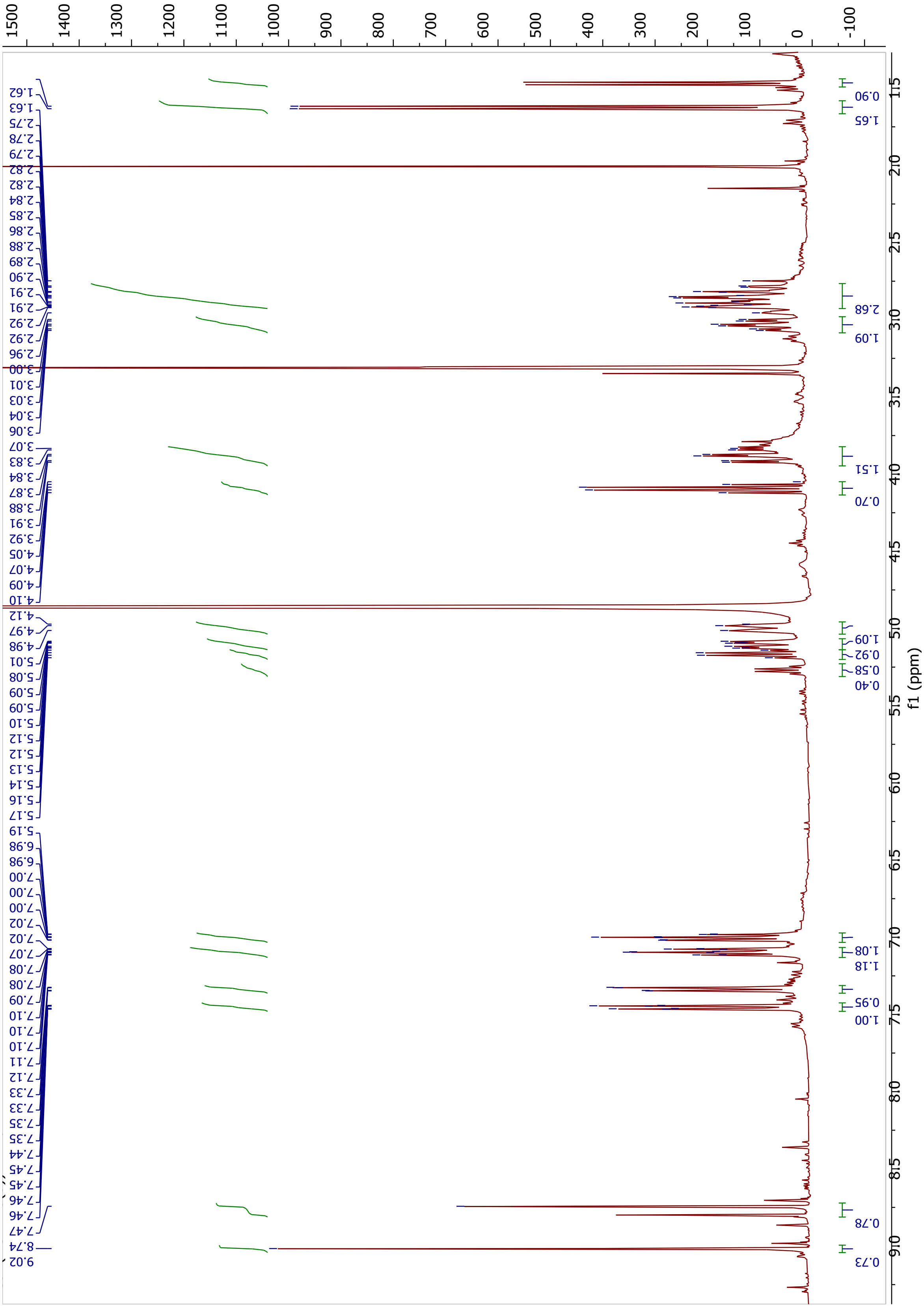

Figure S47. ^{1}H NMR of 3,14-dihydro angustoline (**13**) in CD_3OD.

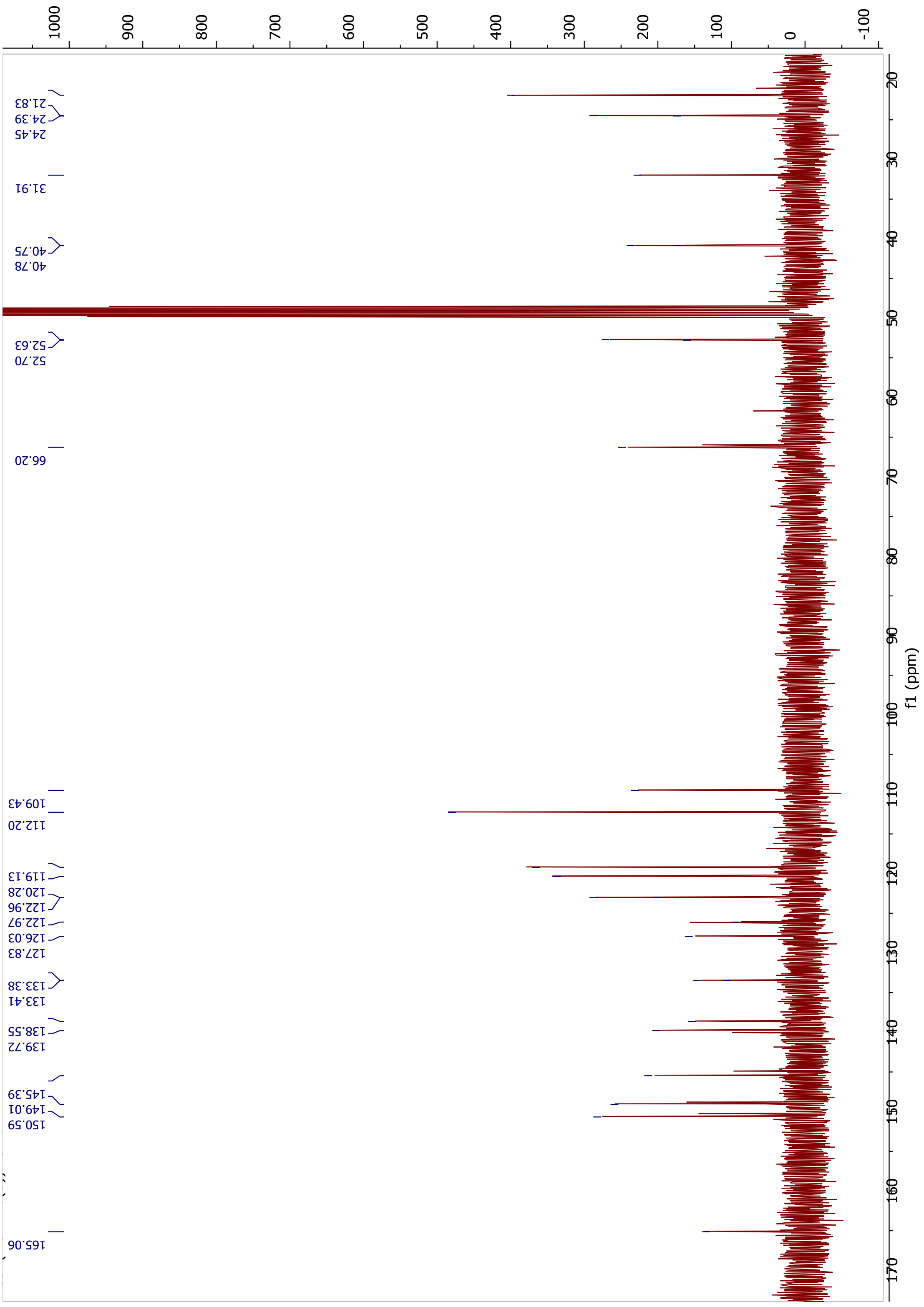

Figure S48. ^{13}C NMR of 3,14-dihydro angustoline (**13**) in CD_3OD.

Generic Display Report

Analysis Info

Analysis Name	E:\Data\MS_MessService\90124000001.d	Acquisition Date	7/7/2022 2:35:17 PM
Method	tune_low_MS_Service_06_22.m	Operator	msc
Sample Name	3,14 Dihydro angustoline	Instrument	maXis
Comment	Traxler / Brecker<br>Ergebnis +/- 5ppm<br>ACN / MeOH + 1% H2O		

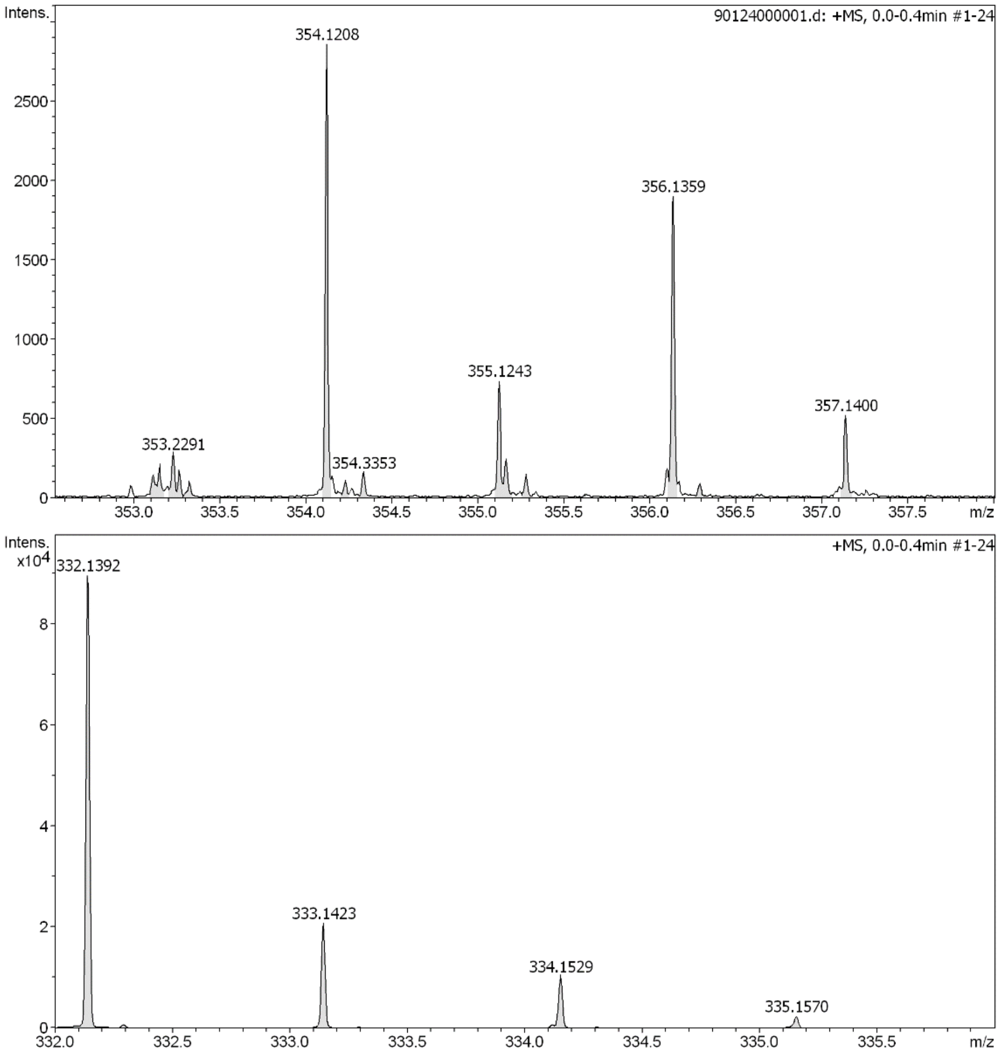

Bruker Compass DataAnalysis 4.1 printed: 7/12/2022 11:54:16 AM by: msc Page 1 of 1

Figure S49. Mass spectrum of 3,14-dihydro angustoline (**13**) here in a mixture next to an angustoline type structure that resulted by dehydrogenation after two years of storage.

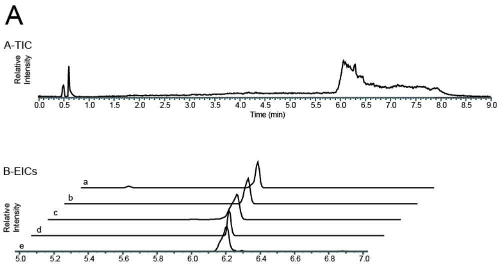

	Compound	RT	m/z (± 5 ppm)
a	10-hydroxy-3-*epi*-pumiloside (**1**)	6.00	529.1817
b	3-*epi*-pumiloside (**2**)	6.05	513.1868
c	10-hydroxy strictosamide * (**4**)	6.07	515.2024
d	angustoline (**12**)	6.11	332.1394
e	strictosamide (**3**)	6.21	499.2075

* and/or 10-hydroxy-3-*epi*-strictosamide (**5**)

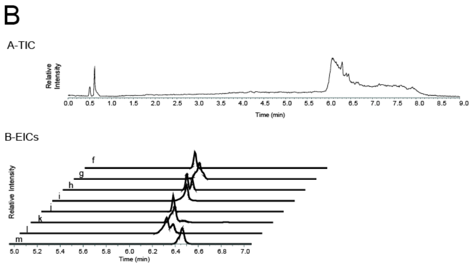

	Compound	RT	m/z (± 5 ppm)
f	5α-carboxystrictosidine	5.92	575.2235
g	3-*epi*-pumiloside (**2**)	6.05	513.1868
h	10-hydroxy strictosamide * (**4**)	6.07	515.2024
i	angustoline (**12**)	6.11	332.1394
j	nauclealomide B/C (**6**/**7**)	6.15	531.1973
k	strictosamide (**3**)	6.21	499.2075
l	3,14-dihydroangustoline (**13**)	6.23	334.1550
m	angustine (**9**)	6.42	314.1288

* and/or 10-hydroxy-3-*epi*-strictosamide (**5**)

Figure S50. LC-MS screening of the crude methanolic extracts from leaves and stem bark of *Nauclea orientalis*. A: Leaves, B: Stembark; TIC: Total ion count (TIC), EIC: Extracted ion chromatograms for *m/z* of authentic standards, RT: Retention time [min].

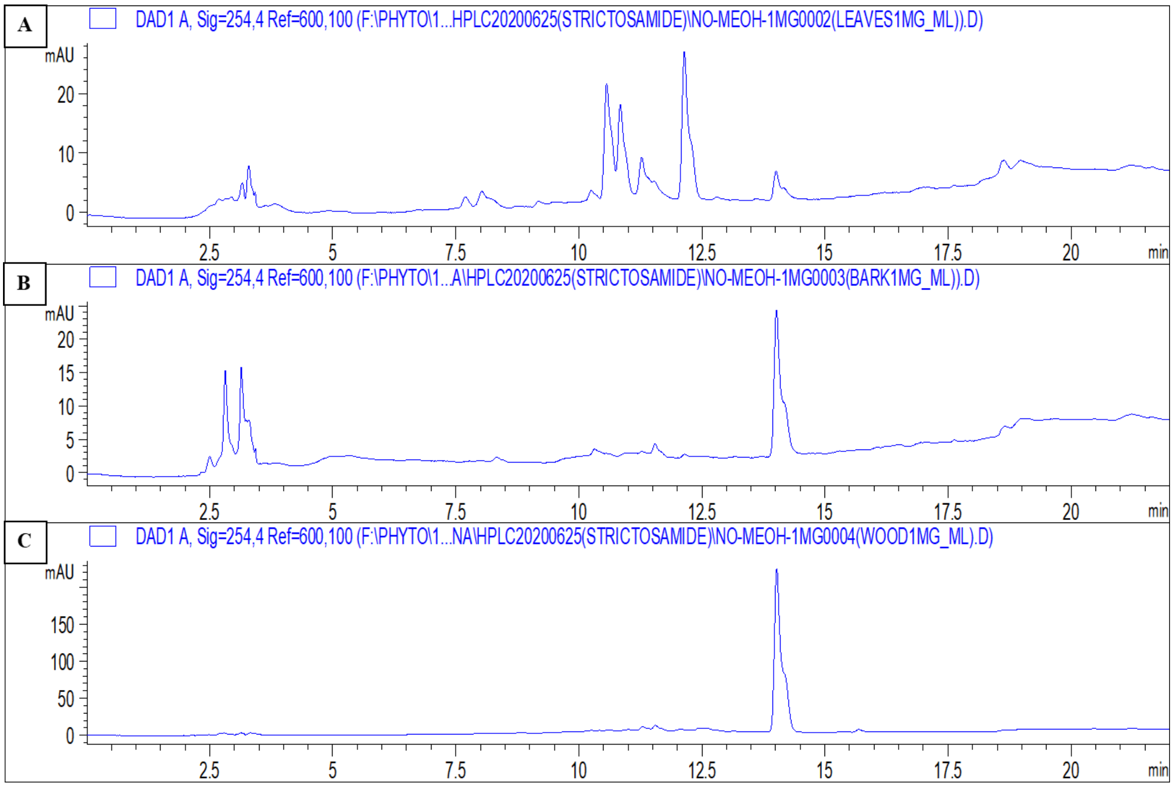

Figure S51. HPLC profile of methanolic extracted of leaves (**A**), bark (**B**) and wood (**C**).

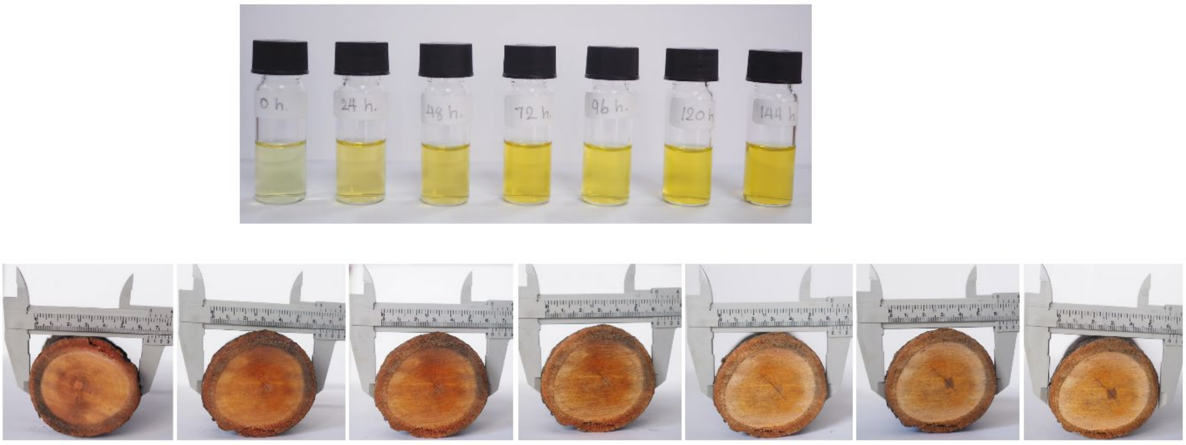

0 h | 24 h | 48 h | 72 h | 96 h | 120 h | 144 h

Figure S52. Above: With methanol rinsed cutting surfaces of *N. orientalis* within one week. Below: Changing colors of the cutting surface of twigs within one week.

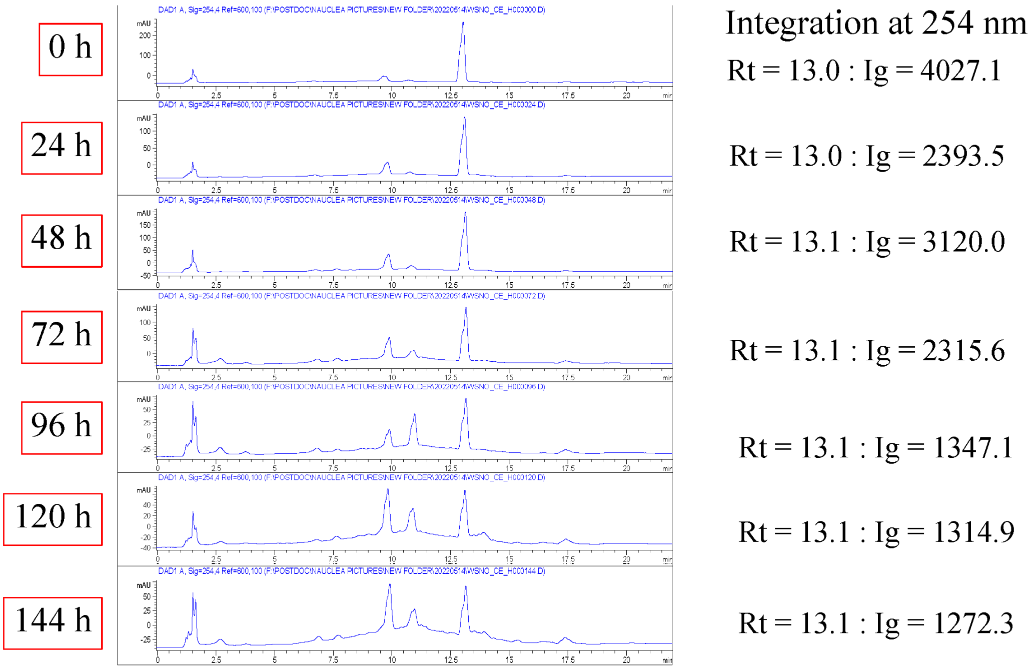

Figure S533. HPLC profiles of the rinsed cutting surfaces of the twigs of *N. orientalis*. The peak at 13.0/13.1 minutes is strictosamide (**1**); Ig indicates the peak area. All samples were injected at the identical concentration. The wavelength of detection was set at 254 nm.

References

S1. Petzelbauer, I.; Splechtna, B.; Nidetzky, B., Galactosyl transfer catalyzed by thermostable β-glycosidases from *Sulfolobus solfataricus* and *Pyrococcus furiosus*: Kinetic studies of the reactions of galactosylated enzyme intermediates with a range of nucleophiles. *J. Biochem.* **2001**, 130(3), 341–349.

S2. Kengen, S. W. M.; Luesink, E. J.; Stams, A. J. M.; Zehnder, A. J. B., Purification and characterization of an extremely thermostable β-glucosidase from the hyperthermophilic archaeon *Pyrococcus furiosus*. *Eur. J. Biochem.* **1993**, 213(1), 305–312.

S3. Brecker, L.; Ribbons, D. W., Biotransformations monitored in situ by proton nuclear magnetic resonance spectroscopy. *Trernds Biotechnol.* **2000**, 18(5), 197–202.

S4. Zhang, Z. Z.; ElSohly, H. N.; Jacob, M. R.; Pasco, D. S.; Walker, L. A.; Clark, A. M., New indole alkaloids from the bark of *Nauclea orientalis*. *J. Nat. Prod.* **2001**, 64(8), 1001–1005.

S5. Itoh, A.; Tanahashi, T.; Nagakura, N., Five tetrahydroisoquinoline-monoterpene glucosides and a tetrahydro-β-carboline-monoterpene glucoside from *Alangium lamarckii*. *J. Nat. Prod.* **1995**, 58(8), 1228–1239.

S6. Kitajima, M.; Yoshida, S.; Yamagata, K.; Nakamura, M.; Takayama, H.; Saito, K.; Seki, H.; Aimi, N., Camptothecin-related alkaloids from hairy roots of *Ophiorrhiza pumila*. *Tetrahedron* **2002**, 58(45), 9169–9178.

S7. Fan, L.; Liao, C. H.; Kang, Q. R.; Zheng, K.; Jiang, Y. C.; He, Z. D., Indole Alkaloids from the leaves of *Nauclea officinalis*. *Molecules* **2016**, 21(8), 968.

S8. Erdelmeier, C. A. J.; Regenass, U.; Rali, T.; Sticher, O., Indole Alkaloids with Invitro Antiproliferative activity from the ammoniacal extract of *Nauclea orientalis*. *Planta Med.* **1992**, 58(1), 43–48.

S9. Sichaem, J.; Surapinit, S.; Siripong, P.; Khumkratok, S.; Jong-Aramruang, J.; Tip-Pyang, S., Two new cytotoxic isomeric indole alkaloids from the roots of *Nauclea orientalis*. *Fitoterapia* **2010**, 81(7), 830–833.
